# Supplementary material for: Fire use practices, knowledge and perceptions in a West African savanna parkland
Source: PLoS One. 2022 May 19;17(5):e0240271. doi: 10.1371/journal.pone.0240271 (PMC9119518; doi:10.1371/journal.pone.0240271)
Supplement: S1 Appendix — (PDF) [file pone.0240271.s001.pdf]

S1 appendix

Questionnaire on the study of people's perceptions and knowledge on the use and the impacts of fire on savanna ecosystems in selected Communities, Northern Region, Ghana.

This questionnaire has been designed to find out reasons why people use fire and to assess their knowledge and perception on the use of fire. Please answer as truthful as possible. You may give your name or not. The information will be treated as anonymously as possible.

Section A - Demographic Characteristics

**District**	**Community**
**Questionnaire No**	**GPS coordinates**
**Name of the Interviewee (Optional)**	**Date of Interview (dd/mm/yy)**
**Gender**	**Ethnic group**
**Age of the Interviewee**	**Household size**
**Marital Status**	**Indigene / settler**
**Occupation**	**Name of Interviewer**

Education (Highest level) ❑ No formal education ❑ Primary ❑ JHS/ Middle school ❑ SHS ❑ Tertiary ❑ Non-Formal ❑ Other (Specify)

Section B - Knowledge and perception on the use of fire

1. **i. Do you use fire in any of the activities below?**

   ❑ **Yes** ❑ **No**

ii. If yes, indicate in which of the following activities you use fire, rank the activities in order of usage of fire in each season and give reasons for the s usage of fire.

**Activity**	**Tick the activities in which you use fire** Wet	Dry	**What are reasons for the use of fire for the activity**
Land preparation			
Hunting			
Pasture management			
Weed control			
Pest control			
Clean farm after harvesting			
Bush clearing around homesteads			
Fire breaks			
Others			

2. **How often do you use fire for the following activities?**

	Once a year	Twice a year	Never
Land preparation for farming			
Land clearing			
Hunting			
Pasture management			
Weed control			
Pest control			
Clean farm after harvesting			
Bush clearing around homesteads			
Charcoal burning			
Fire breaks			
Others			

3. **Are there traditional reasons why fire is used for farming and other activities? Yes No**

ii. **If yes, what are your reasons**

4. **How important are the following to you, when using fire for the activities you ticked above (Question 1 ii)?**

**Fire Attribute**	**Very Important**	**Important**	**Not Important**	**What is/are your reason(s)**
Season of burn				
Intensity of fire				
Duration of burn				
Frequency of fire				
Size of fire				
Pattern of fire				
other				

5. **Are you aware of any effect (s) from the use of fire on the following components of the environment?**

**Component of the Environment**	**Known effect (s)**		**Don't know**
	**Positive**	**Negative**	
Soil			
Vegetation			
Air			
Water			
Animals			
other			

<u>Section C - Fire Management</u>

6. **Do you control fire for the following activities?**

**Activity**	**ALWAYS**	**SOMETIMES**	**NEVER**	**Reason(s) for Control**
Land preparation for farming				
Land clearing				
Hunting				
Pasture management				
Weed control				
Pest control				
Clean farm after harvesting				
Bush clearing around homesteads				

Charcoal burning				
Fire breaks				
Others				

7. **i. Have you ever experienced an incident of fire that was ignited by you and could not be controlled?**

❑ **Yes** ❑ **No**

ii. If yes, how was the fire brought under control?

8. **How is fire managed in this community?**
9. **i. In your opinion is fire good for the Environment?**

❑ **Yes** ❑ **No** ❑ **I do not know**

ii. What are your reasons for your choice above?

10. **Do you agree that we can reduce the use of fire for activities mentioned above?**

❑ Yes ❑ No ❑ Don’t know

11. **If yes, what are the alternative way (s) to using fire in the following activitie**

**Activity**	**Alternative**
Land preparation for farming	
Land clearing	
Hunting	
Pasture management	
Weed control	
Pest control	
Clean farm after harvesting	
Bush clearing around homesteads	
Charcoal burning	

Thank you for your time.
