# Supplementary material for: Fire use practices, knowledge and perceptions in a West African savanna parkland
Source: PLoS One. 2022 May 19;17(5):e0240271. doi: 10.1371/journal.pone.0240271 (PMC9119518; doi:10.1371/journal.pone.0240271)
Supplement: S2 Appendix — (PDF) [file pone.0240271.s002.pdf]

Fire Detections

Ghana

Advanced Fire Information System

Detected Fires between Jan-2017 & Dec-2017

Daily Fire Detection count > 50 are highlighted in orange and Fire detection >=100 are highlighted in red.

Jan-2017		01/01/17	02/01/17	03/01/17	04/01/17	05/01/17	06/01/17	07/01/17	08/01/17	09/01/17	10/01/17	11/01/17	12/01/17	13/01/17	14/01/17	15/01/17	16/01/17	17/01/17	18/01/17	19/01/17	20/01/17	21/01/17	22/01/17	23/01/17	24/01/17	25/01/17	26/01/17	27/01/17	28/01/17	29/01/17	30/01/17	31/01/17	Monthly Total
**Ashanti**	Adansi North	0	0	0	0	0	0	0	0	0	0	0	0	0	0	0	0	0	0	0	0	0	0	0	0	0	1	0	0	0	0	0	**1**
	Afigya Sekyere	0	0	1	0	0	0	0	0	0	0	0	0	0	0	0	0	0	0	0	0	0	0	0	0	0	0	0	0	0	0	3	**4**
	Ahafo Ano North	0	0	1	0	0	0	0	0	0	0	0	0	1	0	0	0	0	0	0	0	0	0	0	0	0	0	0	0	0	0	0	**2**
	Ahafo Ano South	0	0	0	0	0	0	0	0	0	0	0	0	0	0	0	0	0	0	0	0	0	0	0	0	0	1	0	0	0	0	0	**1**
	Amansie East	0	0	0	0	0	0	0	0	0	0	0	0	0	0	0	0	0	0	0	0	0	0	0	2	0	1	0	2	0	0	1	**6**
	Amansie West	0	0	0	0	0	0	0	0	0	0	0	0	0	0	0	0	0	0	0	0	0	1	0	2	0	0	0	0	0	0	0	**3**
	Asante Akim North	1	0	0	3	1	14	0	0	0	0	1	0	3	5	1	1	0	0	0	1	0	0	0	5	0	0	0	5	1	1	5	**48**
	Atwima	0	0	0	0	0	0	0	0	0	0	0	0	0	0	0	0	0	0	0	0	0	0	0	0	0	1	0	0	0	0	0	**1**
	Atwima Mponua	0	0	0	0	0	0	0	0	0	0	0	0	0	0	0	0	0	0	0	0	0	0	0	1	0	0	0	0	0	0	0	**1**
	Bosomtwe-Kwanwoma	0	0	0	0	0	0	0	0	0	0	0	0	0	0	0	0	0	0	0	0	0	1	0	1	0	0	0	0	0	0	0	**2**
	Ejura Sekyedumas	0	2	4	0	0	3	0	0	0	0	0	0	5	1	9	0	0	0	0	0	5	7	0	14	6	19	1	8	1	5	4	**94**
	Kwabre	0	0	0	0	0	0	0	0	0	0	0	0	0	0	0	0	0	0	0	0	0	0	0	1	0	0	0	0	0	0	0	**1**
	Obuasi Municipal	0	0	0	0	0	0	0	0	0	0	0	0	0	0	0	0	0	0	0	0	0	0	0	0	0	1	0	0	0	0	0	**1**
	Offinso	0	0	1	4	0	1	0	0	0	0	3	0	4	0	8	0	0	0	0	0	0	0	0	9	0	3	1	1	1	0	0	**36**
	Sekyere East	7	5	12	50	5	78	5	8	0	1	1	1	75	11	20	12	44	0	5	12	10	49	0	45	0	11	17	42	29	1	15	**571**

Daily Fire Detection count > 50 are highlighted in orange and Fire detection >=100 are highlighted in red.

Jan-2017		01/01/17	02/01/17	03/01/17	04/01/17	05/01/17	06/01/17	07/01/17	08/01/17	09/01/17	10/01/17	11/01/17	12/01/17	13/01/17	14/01/17	15/01/17	16/01/17	17/01/17	18/01/17	19/01/17	20/01/17	21/01/17	22/01/17	23/01/17	24/01/17	25/01/17	26/01/17	27/01/17	28/01/17	29/01/17	30/01/17	31/01/17	Monthly Total
**Ashanti**	Sekyere West	0	3	7	24	1	60	1	0	0	1	2	4	71	9	12	0	3	0	7	9	4	10	0	35	2	61	10	10	9	0	8	**363**
	**Ashanti Total**	8	10	26	81	7	156	6	8	0	2	7	5	159	26	50	13	47	0	12	22	19	68	0	115	8	99	29	68	41	7	36	**1135**
**Brong Ahafo**	Asunafo South	0	0	0	0	0	0	0	0	0	0	0	0	0	0	1	0	0	0	0	0	0	0	0	0	0	0	0	0	0	0	0	**1**
	Asutifi	0	0	0	0	0	1	0	0	0	0	0	0	0	0	0	0	0	0	0	0	0	0	0	6	0	1	3	0	3	0	0	**14**
	Atebubu-Amantin	0	0	5	16	0	13	0	2	0	0	7	0	28	1	5	0	2	0	1	1	8	15	0	32	0	20	6	24	10	0	8	**204**
	Berekum	0	0	0	0	0	1	0	0	0	0	0	0	0	0	0	0	0	0	0	0	0	0	0	1	0	6	0	0	0	0	0	**8**
	Dormaa	0	0	0	0	0	0	0	0	0	0	0	0	0	0	0	0	0	0	0	0	0	0	0	0	0	8	0	0	1	0	2	**11**
	Jaman North	0	1	1	0	0	0	0	0	0	0	0	0	1	0	0	0	0	0	1	0	0	0	0	0	0	0	0	0	3	0	0	**7**
	Kintampo North	21	13	27	22	3	24	3	5	3	2	13	1	33	1	45	0	2	0	0	0	1	22	0	39	0	31	2	8	11	2	10	**344**
	Kintampo South	5	7	26	41	2	19	1	0	0	0	8	5	41	0	55	0	1	0	0	1	5	25	0	34	0	24	1	9	17	2	6	**335**
	Nkoranza	7	15	48	36	1	15	0	2	0	2	6	6	70	4	37	0	3	0	3	3	1	8	0	24	0	24	6	14	6	4	15	**360**
	Pru	10	9	31	63	7	28	0	3	1	0	6	8	41	2	25	1	7	0	1	0	4	14	0	19	0	14	3	4	8	1	8	**318**
	Sene	20	1	31	63	5	119	22	29	5	13	14	16	46	11	44	8	40	0	9	14	15	50	0	70	11	21	7	32	21	18	16	**771**
	Sunyani	0	0	0	0	0	0	0	0	0	0	0	0	0	0	0	0	0	0	0	0	0	0	0	0	0	0	0	0	1	2	6	**9**
	Tain	16	14	27	38	0	18	2	5	7	4	12	1	26	3	17	0	1	2	0	2	0	17	0	18	2	20	6	15	13	8	18	**312**
	Tano North	0	0	0	1	0	0	0	0	0	0	0	0	0	0	1	0	0	0	0	0	0	0	0	4	0	0	0	0	0	0	0	**6**
	Tano South	0	0	0	0	0	0	0	0	0	0	0	0	0	0	0	0	0	0	0	0	0	0	0	1	0	2	4	1	2	0	0	**10**
	Techiman	1	0	0	4	2	2	0	0	0	0	0	0	3	0	4	0	0	0	0	0	0	4	0	3	0	16	1	0	3	0	0	**43**
	**Brong Ahafo Total**	80	60	196	284	20	240	28	46	16	21	66	37	289	22	234	9	56	2	15	21	34	155	0	251	13	187	39	107	99	37	89	**2753**

Daily Fire Detection count > 50 are highlighted in orange and Fire detection >=100 are highlighted in red.

Jan-2017		01/01/17	02/01/17	03/01/17	04/01/17	05/01/17	06/01/17	07/01/17	08/01/17	09/01/17	10/01/17	11/01/17	12/01/17	13/01/17	14/01/17	15/01/17	16/01/17	17/01/17	18/01/17	19/01/17	20/01/17	21/01/17	22/01/17	23/01/17	24/01/17	25/01/17	26/01/17	27/01/17	28/01/17	29/01/17	30/01/17	31/01/17	Monthly Total
**Central**	Abura-Asebu-Kwamankese	0	0	0	0	0	0	0	0	0	0	0	0	1	0	0	0	0	0	0	0	0	0	0	0	0	0	0	0	0	0	0	**1**
	Ajumako-Enyan-Esiam	0	0	0	0	0	1	0	0	0	0	0	0	0	0	0	0	0	0	0	0	0	0	0	0	0	0	0	0	0	0	0	**1**
	Mfantsiman	0	0	0	0	0	0	0	0	0	0	0	0	0	0	0	0	0	0	0	0	0	0	0	0	0	1	0	0	0	0	0	**1**
	Upper Denkyira	0	0	0	0	0	0	0	0	0	0	0	0	0	0	0	0	0	0	0	0	0	0	0	1	0	1	0	0	1	0	1	**4**
	**Central Total**	0	0	0	0	0	1	0	0	0	0	0	0	1	0	0	0	0	0	0	0	0	0	0	1	0	2	0	0	1	0	1	**7**
**Eastern**	Afram Plains	4	6	1	36	7	59	3	9	0	0	4	9	23	7	16	1	5	0	1	5	9	4	0	12	1	6	1	20	9	0	3	**261**
	Akwapim South	0	0	0	0	0	0	0	0	0	0	0	0	0	0	0	0	1	0	0	0	0	0	0	0	0	0	0	2	0	0	0	**3**
	Asuogyaman	0	0	0	0	0	1	0	0	0	0	0	0	0	5	1	0	0	0	1	0	0	0	0	0	0	0	1	0	2	0	0	**11**
	Fanteakwa	0	0	0	5	1	2	0	2	0	0	0	0	4	0	0	0	2	0	0	0	1	0	0	2	0	1	0	1	3	0	0	**24**
	Kwahu South	0	0	0	5	0	13	0	1	0	0	0	0	5	3	0	0	0	0	1	0	0	0	0	2	0	0	1	0	1	0	1	**33**
	Manya Krobo	2	0	0	0	0	2	0	0	0	0	0	0	3	0	0	0	1	0	0	0	0	0	0	2	0	0	0	9	5	0	0	**24**
	Yilo Krobo	0	0	0	0	0	3	0	0	0	1	0	0	0	2	0	0	0	0	4	1	0	0	0	0	0	0	0	1	0	0	1	**13**
	**Eastern Total**	6	6	1	46	8	80	3	12	0	1	4	9	35	17	17	1	9	0	7	6	10	4	0	18	1	7	3	33	20	0	5	**369**
**Greater Accra**	Dangbe East	0	0	0	0	0	4	0	0	0	0	0	0	1	0	0	0	0	0	0	0	0	0	0	0	0	0	0	1	0	0	0	**6**
	Tema	0	0	0	0	0	0	0	0	0	0	0	0	0	0	0	0	0	0	0	0	2	0	0	0	0	0	0	0	0	0	0	**2**
	**Greater Accra Total**	0	0	0	0	0	4	0	0	0	0	0	0	1	0	0	0	0	0	0	0	2	0	0	0	0	0	0	1	0	0	0	**8**
**Northern**	Bole	11	3	23	21	5	14	2	3	0	2	14	0	13	0	18	0	1	0	1	0	5	7	0	14	0	13	0	1	9	4	3	**187**
	Bunkpurugu Yunyoo	4	2	0	2	4	6	3	0	0	3	0	2	1	0	2	0	0	0	0	0	1	3	0	1	0	0	0	0	3	0	2	**39**
	Central Gonja	33	8	41	36	2	54	0	18	1	0	14	3	37	8	73	0	3	0	1	5	8	39	0	52	1	22	7	20	12	1	19	**518**
	East Gonja	31	22	22	39	19	90	7	9	0	3	17	19	46	1	62	0	19	0	13	7	28	22	0	99	2	44	12	8	16	12	13	**682**

Daily Fire Detection count > 50 are highlighted in orange and Fire detection >=100 are highlighted in red.

Jan-2017		01/01/17	02/01/17	03/01/17	04/01/17	05/01/17	06/01/17	07/01/17	08/01/17	09/01/17	10/01/17	11/01/17	12/01/17	13/01/17	14/01/17	15/01/17	16/01/17	17/01/17	18/01/17	19/01/17	20/01/17	21/01/17	22/01/17	23/01/17	24/01/17	25/01/17	26/01/17	27/01/17	28/01/17	29/01/17	30/01/17	31/01/17	Monthly Total
**Northern**	East Mamprusi	2	3	2	2	4	8	0	1	0	2	0	0	0	0	0	0	2	0	0	1	0	4	0	4	0	3	1	1	0	2	7	**49**
	Gushiegu	30	17	19	13	14	23	5	11	1	2	15	9	3	5	15	1	3	0	5	9	9	23	3	71	0	4	11	12	24	37	16	**410**
	Karaga	13	4	2	0	11	26	1	11	2	2	7	6	4	7	17	0	9	0	3	8	12	19	0	23	0	4	2	7	16	0	4	**220**
	Nanumba North	10	0	2	7	21	14	3	8	1	2	7	5	7	6	12	0	10	0	4	4	18	9	0	43	0	14	0	19	14	10	8	**258**
	Nanumba South	0	2	1	1	4	7	4	2	0	3	6	0	4	3	7	0	14	0	0	0	29	9	1	31	0	15	1	5	11	2	5	**167**
	Saboba Chereponi	15	10	5	6	3	12	5	14	5	19	12	4	3	2	41	0	2	0	13	19	9	15	0	13	7	4	1	9	8	10	18	**284**
	Savelugu Nanton	8	4	0	8	0	7	0	1	0	0	11	2	5	0	14	0	1	0	0	3	0	11	0	11	1	2	0	0	0	0	6	**95**
	Sawa-Tuna-Kalba	2	1	4	9	1	7	0	3	0	0	1	0	5	0	6	4	1	0	0	0	2	4	0	0	0	2	0	1	2	0	3	**58**
	Tamale	1	0	0	12	0	2	0	1	0	0	2	4	1	0	5	0	0	0	0	0	0	0	0	5	0	0	0	0	0	0	0	**33**
	Tolon-Kumbungu	12	5	18	15	0	6	0	1	0	1	10	0	3	0	6	0	0	0	0	0	0	9	0	8	0	3	0	0	2	0	1	**100**
	West Gonja	39	29	37	43	9	26	1	18	8	4	34	9	30	0	32	0	24	3	2	6	10	41	0	63	1	35	15	14	18	1	29	**581**
	West Mamprusi	5	0	12	8	1	12	1	2	0	1	3	1	5	1	5	0	1	0	0	2	6	4	0	10	0	2	0	6	6	1	12	**107**
	Yendi	29	2	12	4	7	29	1	13	0	8	33	11	15	5	42	0	14	0	2	3	21	40	0	45	1	12	1	30	28	6	23	**437**
	Zabzugu Tatale	5	0	3	4	7	13	14	12	2	3	5	6	9	6	22	0	6	0	2	4	4	9	1	5	0	19	3	5	3	3	12	**187**
	**Northern Total**	250	112	203	230	112	356	47	128	20	55	191	81	191	44	379	5	110	3	46	71	162	268	5	498	13	198	54	138	172	89	181	**4412**
**Upper East**	Bawku Municipal	0	0	0	0	0	0	0	0	0	0	2	0	0	0	0	0	1	0	0	0	0	0	0	0	0	0	0	2	0	0	0	**5**
	Bawku West	3	0	0	0	0	2	1	0	0	0	2	0	0	0	0	0	0	0	0	2	0	0	0	0	0	0	0	0	0	0	0	**10**
	Bolgatanga	0	0	0	0	0	0	0	2	0	0	0	0	0	0	0	0	1	0	0	0	0	0	0	0	0	0	0	1	0	0	1	**5**
	Bongo	0	0	0	0	0	2	0	0	0	0	0	0	0	0	1	0	0	0	0	0	0	0	0	0	0	0	0	0	0	0	0	**3**
	Builsa	3	2	1	0	0	1	0	1	0	2	0	0	0	0	1	0	0	0	0	1	0	2	0	2	0	0	2	1	0	0	0	**19**

Daily Fire Detection count > 50 are highlighted in orange and Fire detection >=100 are highlighted in red.

Jan-2017		01/01/17	02/01/17	03/01/17	04/01/17	05/01/17	06/01/17	07/01/17	08/01/17	09/01/17	10/01/17	11/01/17	12/01/17	13/01/17	14/01/17	15/01/17	16/01/17	17/01/17	18/01/17	19/01/17	20/01/17	21/01/17	22/01/17	23/01/17	24/01/17	25/01/17	26/01/17	27/01/17	28/01/17	29/01/17	30/01/17	31/01/17	Monthly Total
**Upper East**	Garu Tempane	2	0	0	0	2	2	0	0	0	0	0	0	0	0	0	0	0	0	0	0	0	0	0	2	0	0	0	0	0	0	0	**8**
	Kassena Nankana	3	0	2	1	0	2	0	1	0	0	0	0	0	0	0	0	1	0	0	0	0	0	0	1	0	0	0	0	1	0	5	**17**
	Talensi Nabdam	0	0	2	1	1	3	0	0	0	0	0	3	3	0	2	0	2	0	0	0	0	0	0	0	0	0	0	2	0	0	0	**19**
	**Upper East Total**	11	2	5	2	3	12	1	4	0	2	4	3	3	0	4	0	5	0	0	3	0	2	0	5	0	0	2	6	1	0	6	**86**
**Upper West**	Jirapa Lambussie	1	0	1	0	0	3	0	1	0	0	0	0	1	0	1	0	0	0	0	0	0	0	0	0	0	1	1	2	0	0	0	**12**
	Lawra	0	0	4	0	0	1	0	2	0	0	0	0	0	0	1	0	1	0	0	0	0	0	0	0	0	0	0	0	0	0	0	**9**
	Nadowli	0	1	2	2	1	1	0	0	0	0	3	0	0	0	4	0	1	0	0	0	0	1	0	0	0	1	0	0	0	0	0	**17**
	Sissala East	5	0	18	6	2	4	0	4	3	4	4	0	2	0	6	0	1	0	0	5	1	8	0	6	0	2	4	1	0	0	1	**87**
	Sissala West	7	2	0	4	1	2	0	6	0	0	0	0	2	0	3	0	2	0	0	3	0	1	0	0	0	1	0	0	2	0	2	**38**
	Wa	1	0	1	0	0	3	0	3	0	0	0	0	0	0	1	0	0	0	0	0	0	2	0	3	0	1	0	1	0	0	7	**23**
	Wa East	7	2	6	7	0	2	0	2	2	2	7	0	0	0	11	0	1	0	0	2	2	2	0	5	0	4	0	2	5	2	5	**78**
	Wa West	0	0	0	2	0	1	0	0	0	0	0	0	0	0	1	0	0	0	0	0	0	2	0	0	0	0	0	0	0	0	0	**6**
	**Upper West Total**	21	5	32	21	4	17	0	18	5	6	14	0	5	0	28	0	6	0	0	10	3	16	0	14	0	10	5	6	7	2	15	**270**
**Volta**	Adaklu Anyigbe	3	1	1	5	1	3	0	6	0	1	0	0	0	0	0	0	1	0	0	0	3	2	0	6	0	2	2	18	5	0	0	**60**
	Akatsi	0	0	0	0	0	3	0	0	0	0	0	0	0	0	0	0	0	0	0	0	0	1	0	0	0	0	0	0	2	0	1	**7**
	Ho	2	0	1	1	0	4	1	0	0	3	0	0	0	0	1	0	2	0	0	0	0	3	0	0	0	2	3	8	1	0	1	**33**
	Hohoe	0	0	0	5	0	2	2	0	0	0	0	0	0	0	0	2	3	0	0	0	1	0	0	0	0	0	0	1	3	0	3	**22**
	Jasikan	5	0	2	0	3	5	3	0	0	0	0	0	0	3	5	1	0	0	0	0	0	2	1	2	2	0	0	7	6	0	3	**50**
	Kadjebi	0	0	0	0	0	0	0	0	0	0	0	1	0	0	0	0	0	0	0	0	0	0	0	0	0	0	0	0	0	0	1	**2**
	Keta	0	0	0	0	0	0	0	0	0	0	0	0	0	0	0	0	0	0	0	0	1	0	0	0	0	0	0	0	0	0	0	**1**

Daily Fire Detection count > 50 are highlighted in orange and Fire detection >=100 are highlighted in red.

Jan-2017		01/01/17	02/01/17	03/01/17	04/01/17	05/01/17	06/01/17	07/01/17	08/01/17	09/01/17	10/01/17	11/01/17	12/01/17	13/01/17	14/01/17	15/01/17	16/01/17	17/01/17	18/01/17	19/01/17	20/01/17	21/01/17	22/01/17	23/01/17	24/01/17	25/01/17	26/01/17	27/01/17	28/01/17	29/01/17	30/01/17	31/01/17	Monthly Total
**Volta**	Ketu	0	0	0	0	0	0	0	0	0	0	0	0	0	0	0	0	0	0	0	0	0	0	0	0	0	0	0	1	0	0	0	**1**
	Kpandu	0	0	1	0	0	2	0	0	0	0	0	0	0	0	0	0	2	0	0	0	1	1	0	1	0	0	0	0	4	0	5	**17**
	Krachi	11	0	15	12	3	27	1	1	0	0	8	5	9	2	23	0	7	0	0	2	3	5	0	25	0	5	0	4	4	0	10	**182**
	Krachi East	0	4	0	7	0	9	0	0	0	0	0	0	4	5	11	0	0	0	1	1	3	7	2	8	0	12	0	5	7	3	4	**93**
	Nkwanta	5	0	4	13	10	44	8	1	3	5	9	4	32	9	24	0	9	0	9	9	7	5	2	30	0	28	11	8	21	17	7	**334**
	North Tongu	0	0	0	2	0	2	0	0	0	1	0	0	0	0	0	0	0	0	0	0	0	0	0	0	0	0	0	1	0	0	4	**10**
	South Dayi	0	0	0	0	0	0	0	0	0	0	0	0	0	0	0	0	0	0	0	0	0	0	0	0	0	0	0	3	2	0	0	**5**
	**Volta Total**	26	5	24	45	17	101	15	8	3	10	17	10	45	19	64	3	24	0	10	12	19	26	5	72	2	49	16	56	55	20	39	**817**
**Western**	Aowin-Suaman	0	0	0	0	0	0	0	0	0	0	0	0	0	0	0	0	0	0	0	0	0	0	0	0	0	1	0	0	0	0	1	**2**
	Mpohor Wassa East	0	0	0	0	0	0	0	0	0	0	0	0	0	0	0	0	0	0	0	0	0	1	0	0	0	0	0	0	0	0	0	**1**
	Nzema East	0	0	0	0	0	0	0	0	0	0	0	0	0	0	0	0	0	0	0	0	0	0	0	0	0	0	0	0	0	0	2	**2**
	Sefwi Wiawso	0	0	0	0	0	0	0	0	0	0	0	0	0	0	0	0	0	0	0	0	0	0	0	0	0	0	1	0	0	0	0	**1**
	Wasa Amenfi East	0	0	0	0	0	0	0	0	0	0	0	0	0	0	0	0	0	0	0	0	0	0	0	0	0	1	0	0	0	0	1	**2**
	Wasa Amenfi West	0	0	0	0	0	0	0	0	0	0	0	0	0	0	0	0	0	0	0	0	0	0	0	0	0	0	0	0	0	0	2	**2**
	Wassa West	0	0	0	0	0	0	0	0	0	0	0	0	0	0	0	0	0	0	0	0	0	0	0	0	0	1	0	0	0	0	1	**2**
	**Western Total**	0	0	0	0	0	0	0	0	0	0	0	0	0	0	0	0	0	0	0	0	0	1	0	0	0	3	1	0	0	0	7	**12**
**Provincial Total**		**402**	**200**	**487**	**709**	**171**	**967**	**100**	**224**	**44**	**97**	**303**	**145**	**729**	**128**	**776**	**31**	**257**	**5**	**90**	**145**	**249**	**540**	**10**	**974**	**37**	**555**	**149**	**415**	**396**	**155**	**379**	**9869**

Daily Fire Detection count > 50 are highlighted in orange and Fire detection >=100 are highlighted in red.

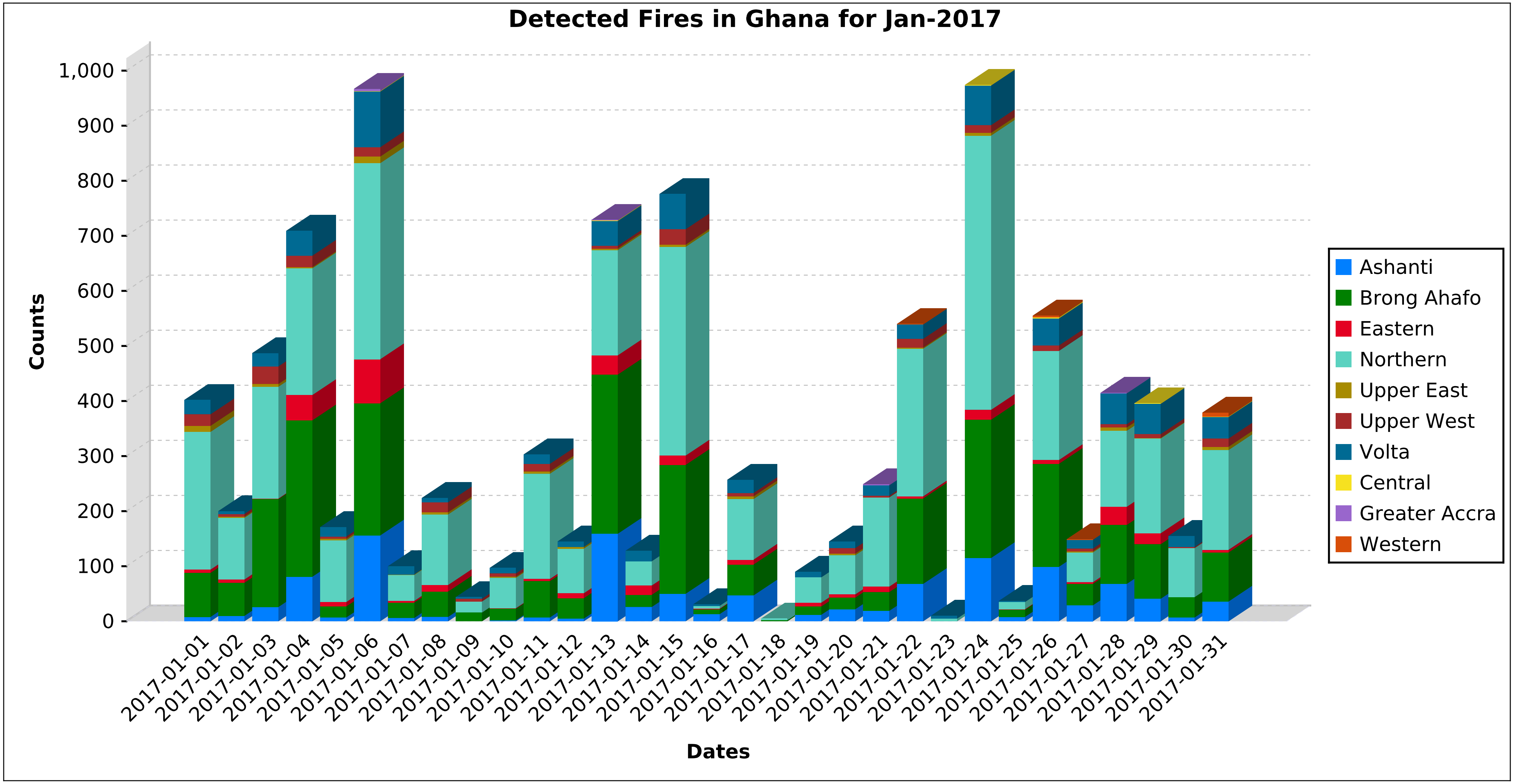

Daily Fire Detection count > 50 are highlighted in orange and Fire detection >=100 are highlighted in red.

Feb-2017		01/02/17	02/02/17	03/02/17	04/02/17	05/02/17	06/02/17	07/02/17	08/02/17	09/02/17	10/02/17	11/02/17	12/02/17	13/02/17	14/02/17	15/02/17	16/02/17	17/02/17	18/02/17	19/02/17	20/02/17	21/02/17	22/02/17	23/02/17	24/02/17	25/02/17	27/02/17	28/02/17	Monthly Total
**Ashanti**	Adansi North	0	1	0	0	0	0	0	0	0	0	0	0	0	0	0	0	0	0	0	0	0	0	0	0	0	0	0	**1**
	Adansi South	0	0	0	0	0	0	0	0	0	0	3	0	0	1	0	0	0	1	2	0	0	0	0	0	0	0	0	**7**
	Afigya Sekyere	0	0	0	0	0	0	1	0	0	0	0	0	0	0	0	0	0	1	0	0	0	0	0	0	0	0	0	**2**
	Ahafo Ano North	0	0	0	0	0	0	0	0	0	0	1	0	0	0	0	0	0	0	0	0	0	0	0	0	0	0	0	**1**
	Ahafo Ano South	0	0	0	0	0	0	0	0	0	0	0	0	0	0	0	0	0	0	0	0	0	0	1	0	0	0	0	**1**
	Amansie Central	0	0	0	0	0	0	2	0	0	0	0	0	0	0	0	0	0	0	0	0	0	0	0	0	0	0	0	**2**
	Amansie East	0	0	0	0	0	0	1	0	0	0	0	0	0	1	0	1	0	0	0	0	0	0	0	0	0	0	0	**3**
	Amansie West	0	2	0	0	0	0	0	0	0	0	0	0	0	0	0	0	0	1	0	0	0	0	0	0	0	0	0	**3**
	Asante Akim North	0	0	0	3	7	0	13	2	3	0	2	0	0	0	0	0	2	1	0	1	0	0	0	0	2	0	0	**36**
	Asante Akim South	0	0	0	0	0	0	0	0	0	0	1	0	0	0	0	0	0	0	0	0	0	0	0	0	0	0	0	**1**
	Atwima	0	1	0	0	0	0	0	0	0	0	0	0	0	1	0	0	0	2	0	0	0	0	0	0	0	0	0	**4**
	Bosomtwe-Kwanwoma	0	1	0	1	0	0	0	0	1	0	1	0	0	0	0	0	0	0	0	0	0	0	0	0	0	0	0	**4**
	Ejisu-Juabeng	0	0	0	0	0	0	1	0	1	0	0	0	0	0	0	0	0	0	0	0	0	0	0	0	0	0	0	**2**
	Ejura Sekyedumas	0	0	0	7	4	2	3	0	0	0	4	0	0	0	0	0	4	4	0	0	0	0	0	0	0	0	0	**28**
	Kwabre	0	0	0	1	0	0	1	0	0	0	0	0	0	0	0	0	0	0	0	1	0	0	0	0	1	0	0	**4**
	Offinso	0	1	0	2	0	0	1	0	6	0	6	0	0	0	0	0	1	6	2	0	0	0	1	0	1	0	0	**27**
	Sekyere East	0	0	0	4	5	0	19	4	9	0	9	0	1	6	0	3	0	15	0	0	2	2	0	0	2	0	0	**81**
	Sekyere West	0	0	0	7	2	1	14	0	14	2	11	0	0	0	0	0	0	8	5	0	0	0	1	0	4	0	0	**69**
	**Ashanti Total**	0	6	0	25	18	3	56	6	34	2	38	0	1	9	0	4	7	39	9	2	2	2	3	0	10	0	0	**276**

Daily Fire Detection count > 50 are highlighted in orange and Fire detection >=100 are highlighted in red.

Feb-2017		01/02/17	02/02/17	03/02/17	04/02/17	05/02/17	06/02/17	07/02/17	08/02/17	09/02/17	10/02/17	11/02/17	12/02/17	13/02/17	14/02/17	15/02/17	16/02/17	17/02/17	18/02/17	19/02/17	20/02/17	21/02/17	22/02/17	23/02/17	24/02/17	25/02/17	27/02/17	28/02/17	**Monthly Total**
**Brong Ahafo**	Asunafo South	0	0	0	0	0	0	0	0	1	0	0	0	0	0	0	0	0	0	0	0	0	0	0	0	0	0	0	**1**
	Asutifi	0	0	0	0	0	0	4	0	0	0	1	1	0	1	0	0	0	1	0	0	0	0	0	0	0	0	0	**8**
	Atebubu-Amantin	0	0	0	3	0	0	12	1	5	0	6	2	0	0	0	1	0	3	0	0	0	0	0	0	0	0	0	**33**
	Berekum	0	0	0	0	0	2	4	0	10	2	0	0	0	1	4	4	0	25	19	0	3	0	0	0	0	0	0	**74**
	Dormaa	0	0	0	0	1	0	0	0	2	0	0	0	0	0	0	0	0	0	0	0	1	0	0	0	0	0	0	**4**
	Jaman North	0	0	0	0	0	0	0	0	0	0	0	0	0	0	0	0	0	1	0	0	0	0	0	0	0	0	0	**1**
	Kintampo North	0	2	2	0	1	0	4	0	6	0	7	0	0	0	0	3	0	3	0	0	2	0	0	0	1	0	0	**31**
	Kintampo South	0	2	0	0	0	2	8	2	3	0	6	0	0	1	0	0	0	12	0	0	4	0	0	0	0	0	0	**40**
	Nkoranza	0	1	0	2	6	2	11	0	9	0	10	0	2	4	0	1	0	11	2	0	0	0	0	0	0	0	0	**61**
	Pru	0	4	2	4	0	0	9	0	3	0	9	0	0	0	0	0	0	2	0	0	2	0	0	0	0	0	0	**35**
	Sene	0	2	0	4	4	2	32	0	7	0	26	0	1	14	0	0	0	14	1	5	0	0	2	0	1	3	0	**118**
	Sunyani	0	1	0	0	0	0	0	0	1	0	1	0	2	0	0	0	0	6	12	5	1	0	2	0	0	0	0	**31**
	Tain	0	4	0	4	4	0	9	0	7	0	7	2	0	2	0	1	0	8	2	0	3	0	4	0	2	0	0	**59**
	Tano North	0	0	0	0	0	0	0	0	0	0	0	0	0	0	0	0	0	0	3	0	0	0	0	0	0	0	0	**3**
	Tano South	0	1	0	0	0	0	2	0	1	0	1	0	0	0	0	0	0	0	0	0	1	0	0	0	0	0	0	**6**
	Techiman	0	1	0	0	0	0	0	0	2	0	2	0	0	0	1	1	0	4	0	0	0	0	0	0	1	0	0	**12**
	**Brong Ahafo Total**	0	18	4	17	16	8	95	3	57	2	76	5	5	23	5	11	0	90	39	10	17	0	8	0	5	3	0	**517**
**Central**	Agona	0	0	0	0	0	0	0	0	1	0	0	0	0	0	0	0	0	0	0	0	0	0	0	0	0	0	0	**1**
	Asikuma Odoben	0	1	0	0	0	0	0	0	0	0	0	0	0	0	0	0	0	0	0	0	0	0	0	0	0	0	0	**1**
	Assin North	0	0	0	0	0	0	2	0	1	0	0	0	0	0	0	0	0	0	0	0	0	0	0	0	0	0	0	**3**

Daily Fire Detection count > 50 are highlighted in orange and Fire detection >=100 are highlighted in red.

Feb-2017		01/02/17	02/02/17	03/02/17	04/02/17	05/02/17	06/02/17	07/02/17	08/02/17	09/02/17	10/02/17	11/02/17	12/02/17	13/02/17	14/02/17	15/02/17	16/02/17	17/02/17	18/02/17	19/02/17	20/02/17	21/02/17	22/02/17	23/02/17	24/02/17	25/02/17	27/02/17	28/02/17	Monthly Total
**Central**	Gomoa	0	1	0	0	0	0	0	0	1	0	0	0	0	0	0	0	0	0	0	0	0	0	0	0	0	0	0	**2**
	Komenda-Edina-Eguafo-	0	2	0	0	0	0	0	0	0	0	0	0	0	0	0	0	0	0	0	0	0	0	0	0	0	0	0	**2**
	Lower Denkyira	0	0	0	0	0	0	0	0	0	0	1	0	0	0	0	0	0	0	0	0	0	0	0	0	0	0	0	**1**
	Upper Denkyira	0	0	0	0	0	0	3	0	0	0	0	0	0	0	0	0	0	0	0	0	0	0	0	0	0	0	0	**3**
	**Central Total**	0	4	0	0	0	0	5	0	3	0	1	0	0	0	0	0	0	0	0	0	0	0	0	0	0	0	0	**13**
**Eastern**	Afram Plains	0	3	0	6	4	3	12	0	7	0	5	1	0	0	0	1	0	2	0	1	3	0	1	0	2	0	0	**51**
	Akwapim North	0	0	0	0	0	0	0	0	0	0	2	0	0	0	0	0	0	0	0	0	0	0	0	0	0	0	0	**2**
	Akwapim South	0	0	0	0	0	0	0	0	0	0	3	0	0	0	0	0	0	1	0	0	0	0	0	0	0	0	0	**4**
	Asuogyaman	0	1	0	0	0	1	4	0	0	0	3	0	0	0	2	0	0	0	0	0	0	0	0	0	0	0	0	**11**
	Atiwa	0	0	0	0	0	0	0	0	1	0	2	0	0	0	0	0	0	1	0	0	0	0	0	0	0	0	0	**4**
	Birim North	0	0	0	0	0	0	0	0	2	0	2	0	0	0	0	0	0	0	0	0	1	0	0	1	0	0	0	**6**
	Birim South	0	0	0	0	0	0	1	0	0	0	1	0	0	0	0	1	0	0	0	0	0	0	0	0	3	0	0	**6**
	East Akim	0	0	0	0	0	0	2	0	0	0	0	0	0	0	0	0	0	0	0	0	0	0	0	0	0	0	0	**2**
	Fanteakwa	0	0	0	2	0	0	5	0	3	0	7	0	0	0	0	0	0	1	0	0	0	0	0	0	0	0	0	**18**
	Kwabibirem	0	0	0	0	0	0	1	0	1	0	1	0	0	0	0	0	0	0	0	0	0	0	0	0	0	0	0	**3**
	Kwahu South	0	0	0	1	0	0	2	0	6	0	4	0	0	0	0	0	0	0	0	0	0	0	0	0	1	0	0	**14**
	Kwahu West	0	0	0	0	0	0	0	0	3	0	3	0	0	0	0	0	0	0	0	0	0	0	0	0	0	0	0	**6**
	Manya Krobo	0	0	0	3	0	0	4	0	2	0	7	0	0	2	0	0	0	1	0	0	0	0	0	0	0	0	0	**19**
	Suhum Kraboa	0	1	0	0	0	0	0	0	1	0	1	0	0	0	0	0	0	0	0	0	0	0	0	0	0	0	0	**3**

Daily Fire Detection count > 50 are highlighted in orange and Fire detection >=100 are highlighted in red.

Feb-2017		01/02/17	02/02/17	03/02/17	04/02/17	05/02/17	06/02/17	07/02/17	08/02/17	09/02/17	10/02/17	11/02/17	12/02/17	13/02/17	14/02/17	15/02/17	16/02/17	17/02/17	18/02/17	19/02/17	20/02/17	21/02/17	22/02/17	23/02/17	24/02/17	25/02/17	27/02/17	28/02/17	Monthly Total
**Eastern**	Yilo Krobo	0	2	0	2	0	0	1	0	2	0	1	0	0	0	0	0	0	0	0	0	0	0	0	0	0	0	0	**8**
	**Eastern Total**	0	7	0	14	4	4	32	0	28	0	42	1	0	2	2	2	0	6	0	1	4	0	1	1	6	0	0	**157**
**Greater Accra**	Dangbe East	0	0	0	1	0	0	3	0	0	0	0	0	0	0	0	0	0	0	0	0	0	0	0	0	0	0	0	**4**
	Dangbe West	0	0	0	1	0	0	0	0	0	0	0	0	0	0	0	0	0	0	0	0	0	0	0	0	0	0	0	**1**
	**Greater Accra Total**	0	0	0	2	0	0	3	0	0	0	0	0	0	0	0	0	0	0	0	0	0	0	0	0	0	0	0	**5**
**Northern**	Bole	0	0	0	0	0	0	5	0	2	0	2	3	0	0	0	4	0	0	0	1	0	0	0	0	0	0	0	**17**
	Bunkpurugu Yunyoo	0	0	0	6	0	0	0	0	0	0	3	1	0	0	0	0	0	0	0	0	0	0	0	0	0	0	0	**10**
	Central Gonja	0	7	0	11	3	0	14	0	9	0	11	0	1	4	0	4	0	4	0	0	0	0	0	0	1	0	2	**71**
	East Gonja	0	6	1	17	7	0	26	2	19	0	16	0	1	13	6	7	0	16	0	4	9	2	1	0	9	3	3	**168**
	East Mamprusi	0	0	0	0	0	0	1	0	0	0	6	0	0	0	0	1	0	0	0	0	0	0	0	0	0	0	0	**8**
	Gushiegu	3	8	1	5	11	1	3	0	11	0	6	0	0	0	1	5	7	9	0	0	1	0	0	0	0	0	0	**72**
	Karaga	0	1	0	11	5	1	2	1	3	0	1	0	1	0	0	0	0	1	0	0	0	0	0	0	0	0	0	**27**
	Nanumba North	0	3	0	4	1	2	7	0	6	0	4	0	8	1	2	7	0	7	0	0	3	0	4	0	4	1	1	**65**
	Nanumba South	0	0	0	17	2	4	6	0	10	0	8	0	3	2	0	1	0	1	0	1	0	4	4	0	0	0	0	**63**
	Saboba Chereponi	7	2	0	19	12	6	14	0	10	0	7	2	5	4	8	4	1	4	0	2	5	0	0	0	9	2	0	**123**
	Savelugu Nanton	0	1	0	1	0	0	0	0	0	0	0	0	0	0	0	0	0	0	0	0	1	0	0	0	0	0	0	**3**
	Sawa-Tuna-Kalba	0	1	0	0	0	0	4	0	3	0	3	0	0	1	0	1	0	0	0	0	0	3	0	0	3	0	0	**19**
	Tamale	0	0	0	0	1	0	0	0	0	0	0	1	0	0	0	0	0	0	0	0	0	0	0	0	0	0	0	**2**
	Tolon-Kumbungu	0	0	0	0	0	0	0	0	1	2	9	0	1	1	0	0	0	0	0	0	1	0	0	0	0	0	0	**15**
	West Gonja	0	3	0	12	8	1	25	0	22	0	22	0	1	9	2	13	0	19	8	4	5	3	0	0	3	0	0	**160**

Daily Fire Detection count > 50 are highlighted in orange and Fire detection >=100 are highlighted in red.

Feb-2017		01/02/17	02/02/17	03/02/17	04/02/17	05/02/17	06/02/17	07/02/17	08/02/17	09/02/17	10/02/17	11/02/17	12/02/17	13/02/17	14/02/17	15/02/17	16/02/17	17/02/17	18/02/17	19/02/17	20/02/17	21/02/17	22/02/17	23/02/17	24/02/17	25/02/17	27/02/17	28/02/17	Monthly Total
**Northern**	West Mamprusi	0	0	0	5	0	0	4	4	2	0	7	0	0	0	2	1	0	1	0	0	0	0	0	0	0	0	0	**26**
	Yendi	1	18	4	30	12	3	10	15	20	0	30	1	9	14	7	10	0	4	0	4	3	2	2	0	4	0	0	**203**
	Zabzugu Tatale	0	1	0	12	2	2	6	0	12	0	19	1	1	0	2	6	0	2	0	0	0	0	1	0	3	0	0	**70**
	**Northern Total**	11	51	6	150	64	20	127	22	130	2	154	9	31	49	30	64	8	68	8	16	28	14	12	0	36	6	6	**1122**
**Upper East**	Bawku West	0	0	0	0	0	0	0	0	2	0	0	0	0	0	0	0	0	0	0	0	0	0	0	0	1	0	0	**3**
	Bolgatanga	0	0	0	1	0	0	0	0	0	0	0	0	0	0	0	0	0	0	0	0	0	0	0	0	0	0	0	**1**
	Builsa	0	4	0	2	0	0	1	0	6	0	1	0	0	0	0	0	0	1	0	0	0	0	0	0	0	0	0	**15**
	Kassena Nankana	0	0	0	0	0	0	0	0	1	0	2	0	0	0	0	0	0	1	0	0	0	0	0	0	0	0	0	**4**
	Talensi Nabdam	0	0	0	2	0	0	0	0	0	0	0	0	0	3	0	0	0	1	0	0	0	0	0	0	0	0	0	**6**
	**Upper East Total**	0	4	0	5	0	0	1	0	9	0	3	0	0	3	0	0	0	3	0	0	0	0	0	0	1	0	0	**29**
**Upper West**	Jirapa Lambussie	0	0	0	0	0	0	0	0	1	0	0	0	0	0	0	0	0	0	0	0	0	0	0	0	0	0	0	**1**
	Lawra	0	0	0	0	3	0	0	0	0	0	0	0	0	0	0	0	0	0	1	0	0	0	0	0	0	0	0	**4**
	Nadowli	0	0	0	0	0	0	0	0	0	0	0	0	0	0	0	0	0	0	0	0	0	0	0	0	0	0	3	**3**
	Sissala East	0	0	0	2	2	1	2	0	3	0	1	0	0	0	0	1	0	0	2	0	1	0	0	0	0	0	0	**15**
	Sissala West	0	0	0	0	0	0	1	0	2	0	0	0	0	1	0	1	0	0	0	0	0	0	1	0	0	0	0	**6**
	Wa	0	0	0	0	0	0	0	0	1	0	0	0	0	2	0	0	0	0	0	0	0	0	0	0	0	0	0	**3**
	Wa East	0	0	0	2	2	0	1	0	3	0	5	0	0	3	0	2	0	3	0	0	0	0	0	0	1	0	0	**22**
	Wa West	0	0	0	0	0	0	0	0	1	0	0	0	0	0	0	0	0	0	0	0	0	0	0	0	0	0	0	**1**
	**Upper West Total**	0	0	0	4	7	1	4	0	11	0	6	0	0	6	0	4	0	3	3	0	1	0	1	0	1	0	3	**55**

Daily Fire Detection count > 50 are highlighted in orange and Fire detection >=100 are highlighted in red.

Feb-2017		01/02/17	02/02/17	03/02/17	04/02/17	05/02/17	06/02/17	07/02/17	08/02/17	09/02/17	10/02/17	11/02/17	12/02/17	13/02/17	14/02/17	15/02/17	16/02/17	17/02/17	18/02/17	19/02/17	20/02/17	21/02/17	22/02/17	23/02/17	24/02/17	25/02/17	27/02/17	28/02/17	Monthly Total
**Volta**	Adaklu Anyigbe	0	8	0	2	0	0	2	0	3	0	7	0	0	0	0	0	0	1	0	0	0	2	0	0	0	0	0	**25**
	Akatsi	0	1	0	0	0	0	0	0	0	0	0	0	0	0	0	0	0	0	0	0	0	0	0	0	0	0	0	**1**
	Ho	0	2	0	2	0	0	6	0	1	0	11	0	0	1	0	0	0	3	0	0	0	0	2	0	0	0	0	**28**
	Hohoe	0	0	0	4	1	0	2	0	2	0	2	0	0	0	0	0	0	15	0	0	0	0	0	0	0	0	0	**26**
	Jasikan	0	0	0	5	1	2	18	0	8	0	2	0	1	0	0	1	2	27	0	2	0	0	0	0	1	0	0	**70**
	Kadjebi	0	0	0	1	0	0	7	0	0	0	5	0	0	0	2	0	0	13	0	7	0	0	1	0	3	1	0	**40**
	Keta	0	0	0	0	0	0	0	0	2	0	1	0	0	0	0	0	0	0	0	0	0	0	0	0	0	0	0	**3**
	Ketu	0	0	0	2	0	0	0	0	0	0	0	0	0	0	0	0	0	0	0	0	0	0	0	0	0	0	0	**2**
	Kpandu	0	1	0	1	1	0	1	0	3	0	1	0	0	2	0	0	0	4	0	3	0	0	0	0	0	0	0	**17**
	Krachi	0	7	1	3	1	0	2	3	2	0	3	0	1	0	0	0	0	5	0	0	0	0	0	0	3	0	0	**31**
	Krachi East	0	1	0	6	0	1	16	1	5	0	17	0	2	2	6	1	1	39	0	0	0	0	0	2	1	0	0	**101**
	Nkwanta	0	4	0	17	3	2	40	5	18	0	35	1	0	5	1	0	1	34	0	4	2	0	0	0	8	4	0	**184**
	North Tongu	0	2	0	0	0	0	0	0	0	0	2	0	0	0	0	0	0	0	0	0	0	0	0	0	0	0	0	**4**
	South Dayi	0	1	0	2	0	0	4	0	1	0	2	0	1	0	0	0	0	1	0	0	0	0	1	0	0	0	0	**13**
	South Tongu	0	0	0	0	0	0	0	0	0	0	1	0	0	0	0	0	0	0	0	0	0	0	0	0	0	0	0	**1**
	**Volta Total**	0	27	1	45	7	5	98	9	45	0	89	1	5	10	9	2	4	142	0	16	2	2	4	2	16	5	0	**546**
**Western**	Aowin-Suaman	0	0	0	0	0	0	2	0	0	0	0	0	0	0	0	0	0	0	0	0	0	0	0	0	0	0	0	**2**
	Bia	0	0	0	0	0	0	0	0	0	0	0	0	0	0	0	0	0	0	0	0	0	0	2	0	0	0	0	**2**
	Bibiani Anhwiaso	0	0	0	0	0	0	0	0	0	0	1	0	0	0	0	0	0	0	0	0	0	0	0	0	0	0	0	**1**
	Juabeso	0	0	0	0	0	0	1	0	0	0	0	0	0	0	0	0	0	0	0	0	2	0	0	0	0	0	0	**3**

Daily Fire Detection count > 50 are highlighted in orange and Fire detection >=100 are highlighted in red.

Feb-2017		01/02/17	02/02/17	03/02/17	04/02/17	05/02/17	06/02/17	07/02/17	08/02/17	09/02/17	10/02/17	11/02/17	12/02/17	13/02/17	14/02/17	15/02/17	16/02/17	17/02/17	18/02/17	19/02/17	20/02/17	21/02/17	22/02/17	23/02/17	24/02/17	25/02/17	27/02/17	28/02/17	Monthly Total
**Western**	Mpohor Wassa East	0	2	0	0	0	0	0	0	0	0	0	0	0	0	0	0	0	0	0	0	0	0	0	0	0	0	0	**2**
	Sefwi Wiawso	0	0	0	0	0	0	0	0	2	0	0	1	0	0	0	0	0	0	0	0	0	0	0	0	0	0	1	**4**
	Wasa Amenfi East	0	0	0	0	0	0	0	0	0	0	2	0	0	0	0	0	0	0	0	0	0	0	0	0	1	0	0	**3**
	Wasa Amenfi West	0	1	0	0	0	0	0	0	0	0	0	0	0	0	0	0	0	0	0	0	0	0	0	0	0	0	0	**1**
	Wassa West	0	1	0	0	1	0	1	0	0	0	0	0	0	0	0	0	0	0	0	0	0	0	0	0	0	0	0	**3**
	**Western Total**	0	4	0	0	1	0	4	0	2	0	3	1	0	0	0	0	0	0	0	0	2	0	2	0	1	0	1	**21**
**Provincial Total**		**11**	**121**	**11**	**262**	**117**	**41**	**425**	**40**	**319**	**6**	**412**	**17**	**42**	**102**	**46**	**87**	**19**	**351**	**59**	**45**	**56**	**18**	**31**	**3**	**76**	**14**	**10**	**2741**

Daily Fire Detection count > 50 are highlighted in orange and Fire detection >=100 are highlighted in red.

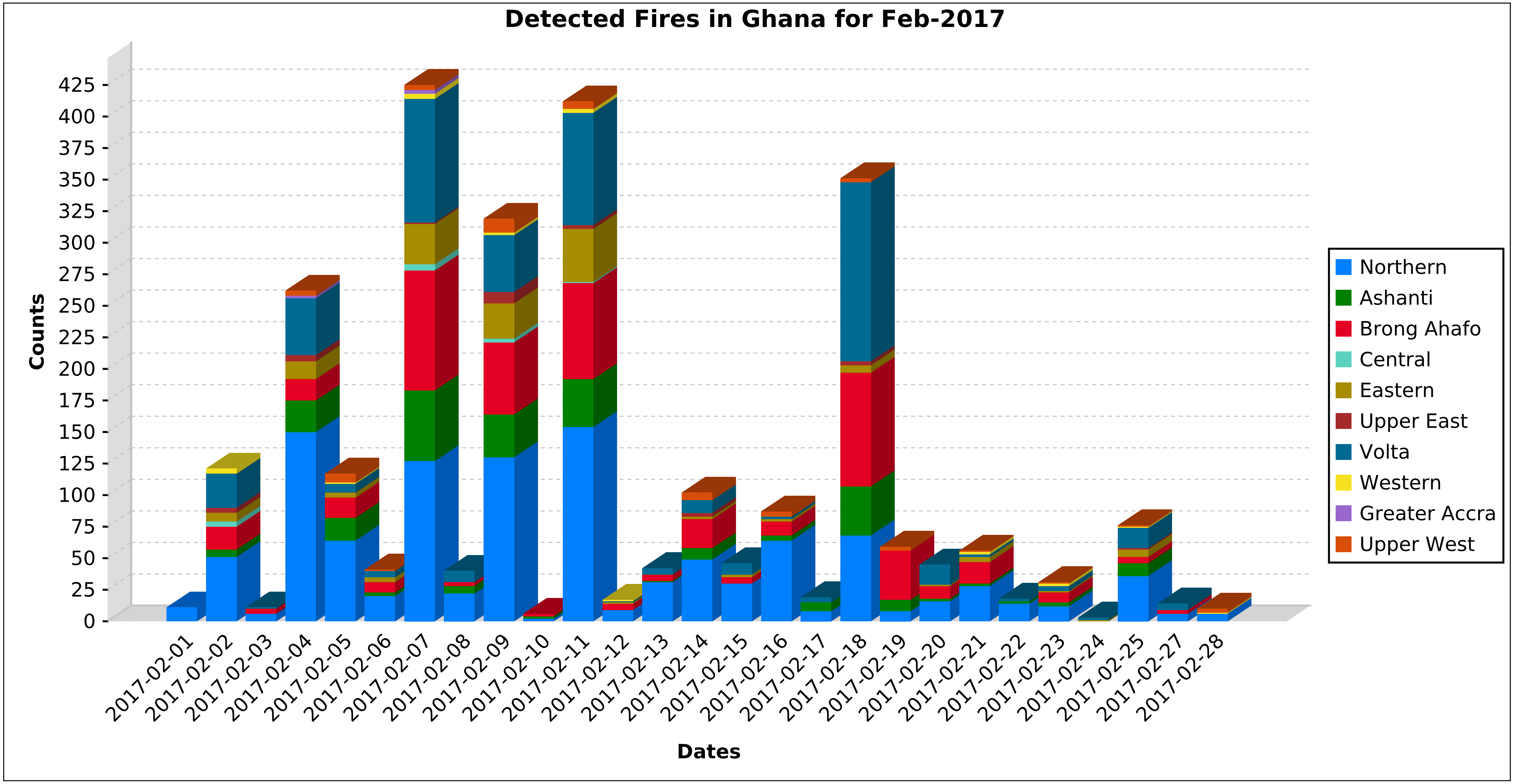

Daily Fire Detection count > 50 are highlighted in orange and Fire detection >=100 are highlighted in red.

Mar-2017		01/03/17	02/03/17	03/03/17	04/03/17	06/03/17	08/03/17	09/03/17	10/03/17	11/03/17	12/03/17	13/03/17	14/03/17	15/03/17	16/03/17	18/03/17	20/03/17	22/03/17	24/03/17	25/03/17	26/03/17	27/03/17	Monthly Total
**Ashanti**	Adansi North	0	0	0	0	0	0	0	0	0	0	0	0	0	0	0	0	0	3	0	0	0	**3**
	Adansi South	0	0	0	0	0	0	0	0	0	0	0	0	0	0	0	0	2	0	0	0	0	**2**
	Ahafo Ano South	0	0	0	4	0	0	0	0	0	0	0	0	0	0	0	0	1	0	0	0	0	**5**
	Amansie Central	0	0	0	1	1	0	1	0	0	0	0	0	0	0	0	0	1	0	0	0	0	**4**
	Amansie East	0	0	0	0	0	0	0	0	0	0	0	0	0	0	0	0	1	0	0	0	0	**1**
	Asante Akim North	0	0	0	1	3	1	1	0	0	0	0	0	1	0	0	0	0	0	0	0	0	**7**
	Atwima	0	0	0	3	0	0	0	0	0	0	0	0	0	0	0	0	0	0	0	0	0	**3**
	Atwima Mponua	0	0	0	2	0	0	0	0	0	0	0	0	0	0	0	0	0	0	0	0	0	**2**
	Bosomtwe-Kwanwoma	0	0	0	0	2	0	0	0	0	0	0	0	0	0	0	0	1	0	0	0	0	**3**
	Ejisu-Juabeng	0	0	0	0	1	0	0	0	0	0	0	0	0	0	0	0	1	0	0	0	0	**2**
	Ejura Sekyedumas	0	0	0	0	0	3	0	0	0	0	0	0	0	0	0	0	0	0	0	0	0	**3**
	Kumasi	0	0	0	0	1	1	0	0	0	0	0	0	0	0	0	0	0	0	0	0	0	**2**
	Kwabre	0	0	0	2	1	0	0	0	0	0	0	0	0	0	0	0	2	0	0	0	0	**5**
	Obuasi Municipal	0	0	0	0	0	0	0	0	0	0	0	0	0	0	0	0	0	3	0	0	0	**3**
	Offinso	0	0	0	3	1	2	0	0	0	0	0	0	0	0	0	0	2	0	0	0	0	**8**
	Sekyere East	0	0	0	3	8	0	0	0	0	0	4	0	1	0	7	0	1	0	0	0	0	**24**
	Sekyere West	0	0	0	2	0	0	0	0	0	0	1	0	0	0	0	0	0	0	0	0	0	**3**
	**Ashanti Total**	0	0	0	21	18	7	2	0	0	0	5	0	2	0	7	0	12	6	0	0	0	**80**

Daily Fire Detection count > 50 are highlighted in orange and Fire detection >=100 are highlighted in red.

Mar-2017		01/03/17	02/03/17	03/03/17	04/03/17	06/03/17	08/03/17	09/03/17	10/03/17	11/03/17	12/03/17	13/03/17	14/03/17	15/03/17	16/03/17	18/03/17	20/03/17	22/03/17	24/03/17	25/03/17	26/03/17	27/03/17	Monthly Total
**Brong Ahafo**	Asutifi	0	0	0	3	0	0	0	0	0	0	0	0	0	0	1	0	0	0	0	0	0	**4**
	Berekum	0	0	0	2	0	1	3	0	0	0	0	3	0	0	0	0	0	0	1	0	0	**10**
	Dormaa	0	0	0	0	0	0	0	0	0	0	0	0	0	0	0	0	0	0	4	0	0	**4**
	Jaman South	0	0	0	0	0	0	0	0	0	0	0	0	0	1	0	0	0	0	0	0	1	**2**
	Kintampo North	0	0	0	0	0	0	1	0	0	0	0	0	0	0	0	0	0	0	0	0	0	**1**
	Kintampo South	0	0	0	2	0	0	2	0	0	0	2	0	2	0	0	0	0	0	1	0	0	**9**
	Nkoranza	0	0	0	1	1	0	0	0	0	0	0	0	0	0	0	0	1	0	0	0	0	**3**
	Pru	0	0	0	3	0	0	0	0	0	0	0	0	2	0	0	0	0	0	0	0	0	**5**
	Sene	0	4	3	5	3	3	0	0	1	0	0	0	0	6	1	0	1	2	0	0	1	**30**
	Sunyani	0	1	0	2	0	1	6	2	0	0	0	0	0	0	0	0	0	0	0	0	0	**12**
	Tain	0	0	0	1	0	3	2	1	0	0	1	0	0	2	0	0	0	0	0	0	0	**10**
	Techiman	0	2	0	0	0	0	0	0	0	0	0	0	0	0	1	0	0	0	0	0	0	**3**
	**Brong Ahafo Total**	0	7	3	19	4	8	14	3	1	0	3	3	4	9	3	0	2	2	6	0	2	**93**
**Central**	Agona	0	0	0	0	0	0	0	0	0	0	0	0	0	0	0	0	1	0	0	0	0	**1**
	Assin North	0	0	0	0	0	0	0	0	0	0	0	0	0	0	0	0	3	0	0	0	0	**3**
	Assin South	0	0	0	0	0	0	0	0	0	0	0	0	0	0	0	0	2	0	0	0	0	**2**
	Awutu Efutu Senya	0	0	0	0	0	0	0	0	0	0	0	0	0	0	0	0	1	0	0	0	0	**1**
	Gomoa	0	0	0	0	0	0	0	0	0	0	0	0	0	0	0	0	3	3	2	0	0	**8**
	Komenda-Edina-Eguafo-	0	0	0	1	0	0	0	0	0	0	0	0	0	0	0	0	0	0	0	0	0	**1**
	Lower Denkyira	0	0	0	0	0	0	0	0	0	0	0	0	0	0	0	0	4	0	0	0	0	**4**

Daily Fire Detection count > 50 are highlighted in orange and Fire detection >=100 are highlighted in red.

Mar-2017		01/03/17	02/03/17	03/03/17	04/03/17	06/03/17	08/03/17	09/03/17	10/03/17	11/03/17	12/03/17	13/03/17	14/03/17	15/03/17	16/03/17	18/03/17	20/03/17	22/03/17	24/03/17	25/03/17	26/03/17	27/03/17	Monthly Total
**Central**	Mfantsiman	0	0	0	0	0	0	0	0	0	0	0	0	0	0	0	0	0	1	0	0	0	**1**
	**Central Total**	0	0	0	1	0	0	0	0	0	0	0	0	0	0	0	0	14	4	2	0	0	**21**
**Eastern**	Afram Plains	0	0	0	1	2	0	0	1	0	0	0	0	0	2	0	0	3	1	1	0	0	**11**
	Akwapim North	0	0	0	0	0	0	0	0	0	0	0	0	0	0	0	0	1	2	0	0	0	**3**
	Akwapim South	0	0	0	0	0	0	0	0	0	0	0	0	0	0	0	0	1	1	0	0	0	**2**
	Asuogyaman	0	0	0	0	1	0	0	0	0	0	0	0	1	0	1	0	2	0	1	0	0	**6**
	Atiwa	0	0	0	1	1	0	0	0	0	0	0	0	0	0	1	0	0	0	0	0	0	**3**
	Birim North	0	0	0	1	1	0	0	0	0	0	0	0	0	0	2	0	4	0	0	0	0	**8**
	Birim South	0	0	0	0	1	0	0	0	0	0	0	0	0	2	0	0	3	0	0	0	0	**6**
	East Akim	0	0	0	0	0	0	0	0	0	0	0	0	0	0	0	0	1	0	0	0	0	**1**
	Kwabibirem	0	0	0	0	2	0	0	0	0	0	1	0	0	0	0	0	1	0	0	0	0	**4**
	Kwahu South	0	0	0	2	1	0	0	0	0	0	0	0	0	0	0	0	1	0	0	0	0	**4**
	Kwahu West	0	0	0	1	1	0	0	0	0	0	0	0	0	0	0	0	0	2	0	0	0	**4**
	Manya Krobo	0	0	0	0	3	0	0	0	0	0	1	0	0	0	3	0	1	1	1	0	0	**10**
	Suhum Kraboa	0	0	0	0	0	0	0	0	0	0	0	0	0	0	0	0	2	0	0	0	0	**2**
	Yilo Krobo	0	0	0	1	0	0	0	0	0	0	0	0	0	0	0	0	1	1	0	0	0	**3**
	**Eastern Total**	0	0	0	7	13	0	0	1	0	0	2	0	1	4	7	0	21	8	3	0	0	**67**
**Greater Accra**	Dangbe East	0	0	0	2	0	0	0	0	0	0	0	0	0	0	0	0	1	0	0	0	0	**3**
	Dangbe West	0	0	0	0	0	0	0	0	0	0	0	0	3	0	0	0	0	0	0	0	0	**3**

Daily Fire Detection count > 50 are highlighted in orange and Fire detection >=100 are highlighted in red.

Mar-2017		01/03/17	02/03/17	03/03/17	04/03/17	06/03/17	08/03/17	09/03/17	10/03/17	11/03/17	12/03/17	13/03/17	14/03/17	15/03/17	16/03/17	18/03/17	20/03/17	22/03/17	24/03/17	25/03/17	26/03/17	27/03/17	Monthly Total
**Greater Accra**	Tema	0	0	0	0	0	0	0	0	0	0	1	0	0	0	0	0	0	0	0	0	0	**1**
	**Greater Accra Total**	0	0	0	2	0	0	0	0	0	0	1	0	3	0	0	0	1	0	0	0	0	**7**
**Northern**	Bole	0	0	0	0	0	0	0	0	0	0	0	0	0	0	0	0	1	0	0	0	0	**1**
	Bunkpurugu Yunyoo	0	0	0	0	0	0	0	0	0	0	1	0	0	0	0	0	0	0	0	0	0	**1**
	Central Gonja	0	0	0	3	1	2	0	0	0	0	0	0	0	1	1	0	2	0	0	0	0	**10**
	East Gonja	1	0	0	2	5	0	2	0	0	0	4	0	1	6	2	0	0	1	0	1	1	**26**
	East Mamprusi	0	0	0	0	0	0	0	0	0	0	2	0	0	0	0	0	0	0	0	0	0	**2**
	Gushiegu	0	0	0	0	0	0	1	0	0	0	0	0	0	0	1	0	0	1	0	0	0	**3**
	Karaga	0	0	0	0	0	0	0	0	0	0	0	0	1	0	0	0	0	0	0	0	0	**1**
	Nanumba North	0	0	0	4	3	6	5	0	2	0	1	0	0	2	0	0	0	0	1	0	0	**24**
	Nanumba South	0	0	0	8	2	6	1	0	0	0	4	0	0	1	0	0	0	0	0	0	0	**22**
	Saboba Chereponi	8	0	0	0	0	0	0	0	0	0	0	0	5	0	2	0	0	0	0	0	0	**15**
	Sawa-Tuna-Kalba	0	0	0	1	0	0	0	0	2	0	0	0	0	0	1	0	0	0	0	0	0	**4**
	Tolon-Kumbungu	0	0	0	1	0	0	0	0	0	0	0	0	0	0	0	0	1	0	0	0	0	**2**
	West Gonja	2	0	2	6	4	0	1	0	7	0	3	0	6	0	2	0	1	0	0	0	0	**34**
	West Mamprusi	0	0	0	2	3	1	0	0	1	0	0	0	1	0	1	0	0	0	0	0	0	**9**
	Yendi	0	1	0	13	3	0	2	0	0	0	0	0	0	0	1	0	0	0	0	0	0	**20**
	Zabzugu Tatale	2	0	0	0	0	0	0	0	2	0	3	0	3	0	1	0	0	0	0	0	1	**12**
	**Northern Total**	13	1	2	40	21	15	12	0	14	0	18	0	17	10	12	0	5	2	1	1	2	**186**

Daily Fire Detection count > 50 are highlighted in orange and Fire detection >=100 are highlighted in red.

Mar-2017		01/03/17	02/03/17	03/03/17	04/03/17	06/03/17	08/03/17	09/03/17	10/03/17	11/03/17	12/03/17	13/03/17	14/03/17	15/03/17	16/03/17	18/03/17	20/03/17	22/03/17	24/03/17	25/03/17	26/03/17	27/03/17	Monthly Total
**Upper East**	Bawku Municipal	0	0	0	0	1	0	0	0	0	0	0	0	0	0	0	0	0	0	0	0	0	**1**
	Bawku West	0	0	0	0	1	0	0	0	0	0	0	0	0	0	0	0	0	0	0	0	0	**1**
	Bolgatanga	0	0	0	1	0	0	0	0	2	0	0	0	0	0	0	0	0	0	0	0	0	**3**
	Garu Tempane	0	0	0	0	0	0	3	0	0	0	0	0	0	0	0	0	0	0	0	0	0	**3**
	Kassena Nankana	0	0	0	1	0	0	0	0	1	0	0	0	0	0	0	2	0	0	0	0	0	**4**
	**Upper East Total**	0	0	0	2	2	0	3	0	3	0	0	0	0	0	0	2	0	0	0	0	0	**12**
**Upper West**	Nadowli	0	0	0	1	0	0	0	0	0	0	0	0	0	0	0	0	0	0	0	0	0	**1**
	Sissala East	0	0	0	2	2	0	0	0	0	0	0	0	0	0	0	0	0	0	1	0	0	**5**
	Sissala West	0	0	0	2	2	0	0	0	0	0	0	0	0	0	2	0	0	0	0	0	0	**6**
	Wa	0	0	0	1	0	0	0	0	0	0	0	0	0	0	0	0	0	0	0	0	0	**1**
	Wa East	0	0	0	0	3	0	1	0	0	0	0	0	0	0	0	0	0	0	0	0	0	**4**
	**Upper West Total**	0	0	0	6	7	0	1	0	0	0	0	0	0	0	2	0	0	0	1	0	0	**17**
**Volta**	Akatsi	0	2	0	0	0	0	0	0	0	0	0	0	0	0	0	0	2	0	0	0	0	**4**
	Ho	0	0	0	0	1	0	0	0	0	0	1	0	0	0	0	0	3	0	0	0	0	**5**
	Hohoe	0	0	0	0	0	0	0	0	0	0	0	0	0	0	0	0	1	0	0	0	0	**1**
	Jasikan	0	0	0	0	1	0	0	0	0	0	0	0	1	0	1	0	2	0	0	0	0	**5**
	Kadjebi	0	0	0	0	1	0	0	0	0	0	0	0	2	0	1	0	2	0	0	0	0	**6**
	Keta	0	0	0	0	0	0	0	0	1	0	0	0	0	0	0	0	0	0	0	0	0	**1**
	Ketu	0	0	0	0	0	0	0	0	0	0	0	0	0	0	0	0	2	0	0	0	0	**2**
	Kpandu	0	0	0	0	0	0	0	0	0	0	0	0	0	0	1	0	1	0	0	0	0	**2**

Mar-2017		01/03/17	02/03/17	03/03/17	04/03/17	06/03/17	08/03/17	09/03/17	10/03/17	11/03/17	12/03/17	13/03/17	14/03/17	15/03/17	16/03/17	18/03/17	20/03/17	22/03/17	24/03/17	25/03/17	26/03/17	27/03/17	Monthly Total
**Volta**	Krachi	0	1	0	0	1	0	0	0	0	0	0	0	0	0	1	0	0	0	0	0	0	**3**
	Krachi East	0	0	0	0	0	0	1	0	0	2	1	0	0	0	1	0	0	0	0	0	0	**5**
	Nkwanta	0	0	1	8	9	1	0	0	0	0	1	0	2	0	3	0	4	0	0	0	0	**29**
	North Tongu	0	0	0	0	0	0	0	0	0	0	0	0	0	0	0	0	3	0	0	0	0	**3**
	South Dayi	0	0	0	0	1	0	0	0	0	0	0	0	0	0	0	0	1	0	0	0	0	**2**
	**Volta Total**	0	3	1	8	14	1	1	0	1	2	3	0	5	0	8	0	21	0	0	0	0	**68**
**Western**	Aowin-Suaman	0	0	0	4	0	0	0	0	0	0	0	0	0	0	1	0	4	0	0	0	0	**9**
	Bia	0	1	0	0	0	0	0	0	0	0	0	0	0	0	0	0	1	0	0	0	0	**2**
	Jomoro	0	0	0	2	0	0	0	0	0	0	0	0	0	0	0	0	2	0	0	0	0	**4**
	Juabeso	0	0	0	1	0	0	0	0	0	0	0	0	0	0	1	0	2	0	0	0	0	**4**
	Mpohor Wassa East	0	0	0	2	1	0	0	0	0	0	0	0	0	0	2	0	5	0	0	0	0	**10**
	Nzema East	0	0	0	2	0	0	0	0	0	0	0	0	0	0	0	0	1	1	1	0	0	**5**
	Sefwi Wiawso	0	0	0	5	1	0	0	0	0	0	0	0	0	0	0	0	2	0	0	0	0	**8**
	Shama Ahanta East	0	0	0	1	0	0	0	0	0	0	0	0	0	0	0	0	3	0	0	0	0	**4**
	Wasa Amenfi East	0	0	0	4	1	0	0	0	0	0	0	0	0	0	0	0	2	0	0	0	0	**7**
	Wasa Amenfi West	0	0	0	0	2	0	0	0	0	0	0	0	0	0	2	0	2	1	0	0	0	**7**
	Wassa West	0	0	0	1	0	0	0	0	0	0	0	0	0	0	0	0	10	0	0	0	0	**11**
	**Western Total**	0	1	0	22	5	0	0	0	0	0	0	0	0	0	6	0	34	2	1	0	0	**71**
**Provincial Total**		**13**	**12**	**6**	**128**	**84**	**31**	**33**	**4**	**19**	**2**	**32**	**3**	**32**	**23**	**45**	**2**	**110**	**24**	**14**	**1**	**4**	**622**

Daily Fire Detection count > 50 are highlighted in orange and Fire detection >=100 are highlighted in red.

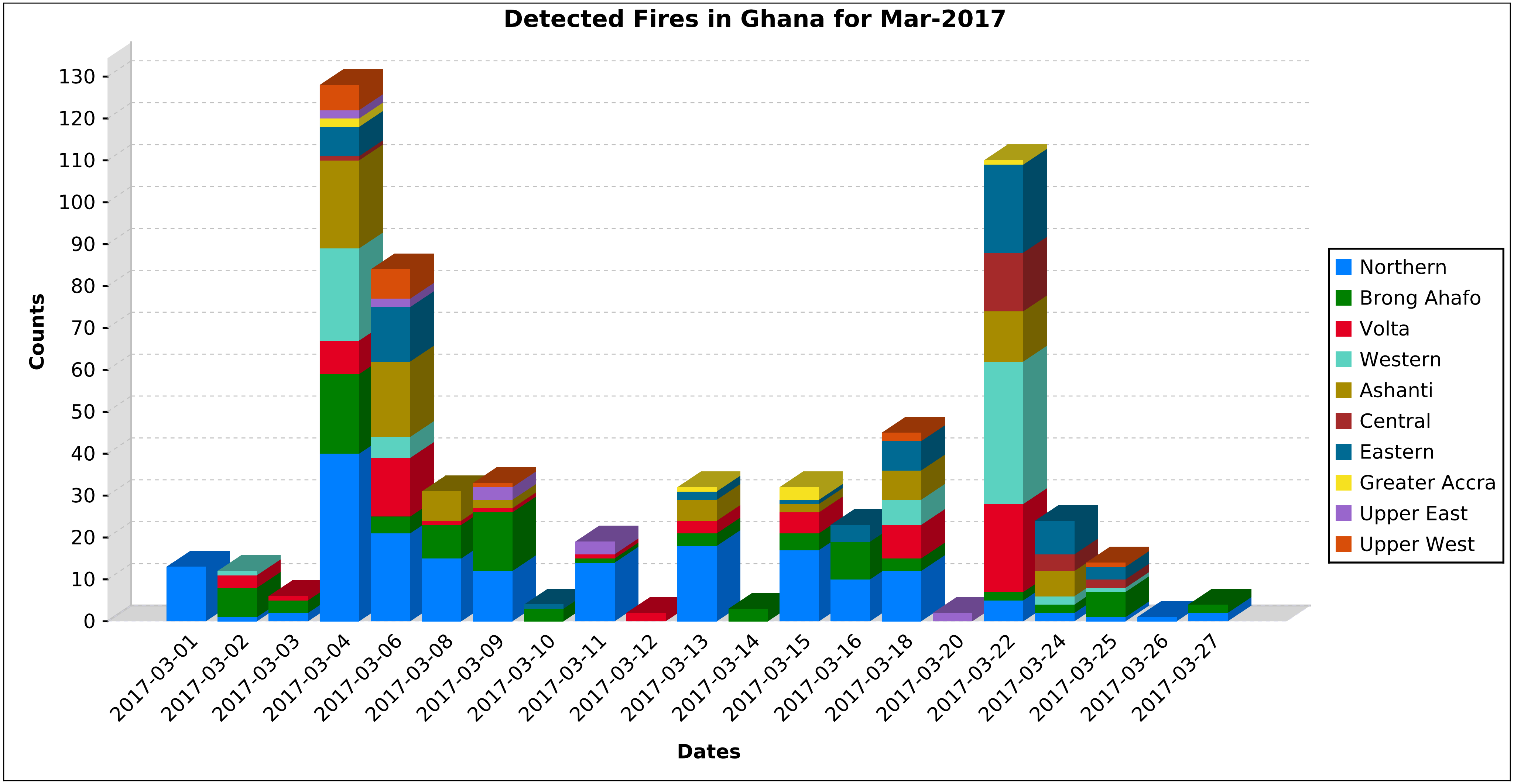

May-2017		07/05/17	09/05/17	14/05/17	16/05/17	19/05/17	22/05/17	23/05/17	28/05/17	Monthly Total
**Ashanti**	Ahafo Ano South	1	0	0	0	0	0	0	0	**1**
	Kumasi	0	0	0	0	0	2	0	0	**2**
	**Ashanti Total**	1	0	0	0	0	2	0	0	**3**
**Brong Ahafo**	Berekum	0	0	0	0	0	0	1	0	**1**
	Dormaa	0	0	0	2	0	0	0	0	**2**
	**Brong Ahafo Total**	0	0	0	2	0	0	1	0	**3**
**Northern**	East Gonja	0	0	2	0	1	0	0	1	**4**
	Gushiegu	0	1	0	0	0	0	0	0	**1**
	Nanumba North	0	0	0	0	0	0	1	0	**1**
	Nanumba South	0	0	0	1	0	0	0	0	**1**
	Saboba Chereponi	2	0	0	0	1	0	0	0	**3**
	West Gonja	0	0	0	0	0	0	1	0	**1**
	Zabzugu Tatale	0	0	0	1	0	0	0	0	**1**
	**Northern Total**	2	1	2	2	2	0	2	1	**12**
**Upper East**	Bawku West	0	1	0	0	0	0	0	0	**1**
	**Upper East Total**	0	1	0	0	0	0	0	0	**1**
**Volta**	Hohoe	0	0	0	0	0	0	0	2	**2**
	Jasikan	0	0	0	0	0	0	1	0	**1**
	**Volta Total**	0	0	0	0	0	0	1	2	**3**

Daily Fire Detection count > 50 are highlighted in orange and Fire detection >=100 are highlighted in red.

May-2017		07/05/17	09/05/17	14/05/17	16/05/17	19/05/17	22/05/17	23/05/17	28/05/17	Monthly Total
**Western**	Wassa West	0	0	0	0	0	0	1	0	**1**
	**Western Total**	0	0	0	0	0	0	1	0	**1**
**Provincial Total**		**3**	**2**	**2**	**4**	**2**	**2**	**5**	**3**	**23**

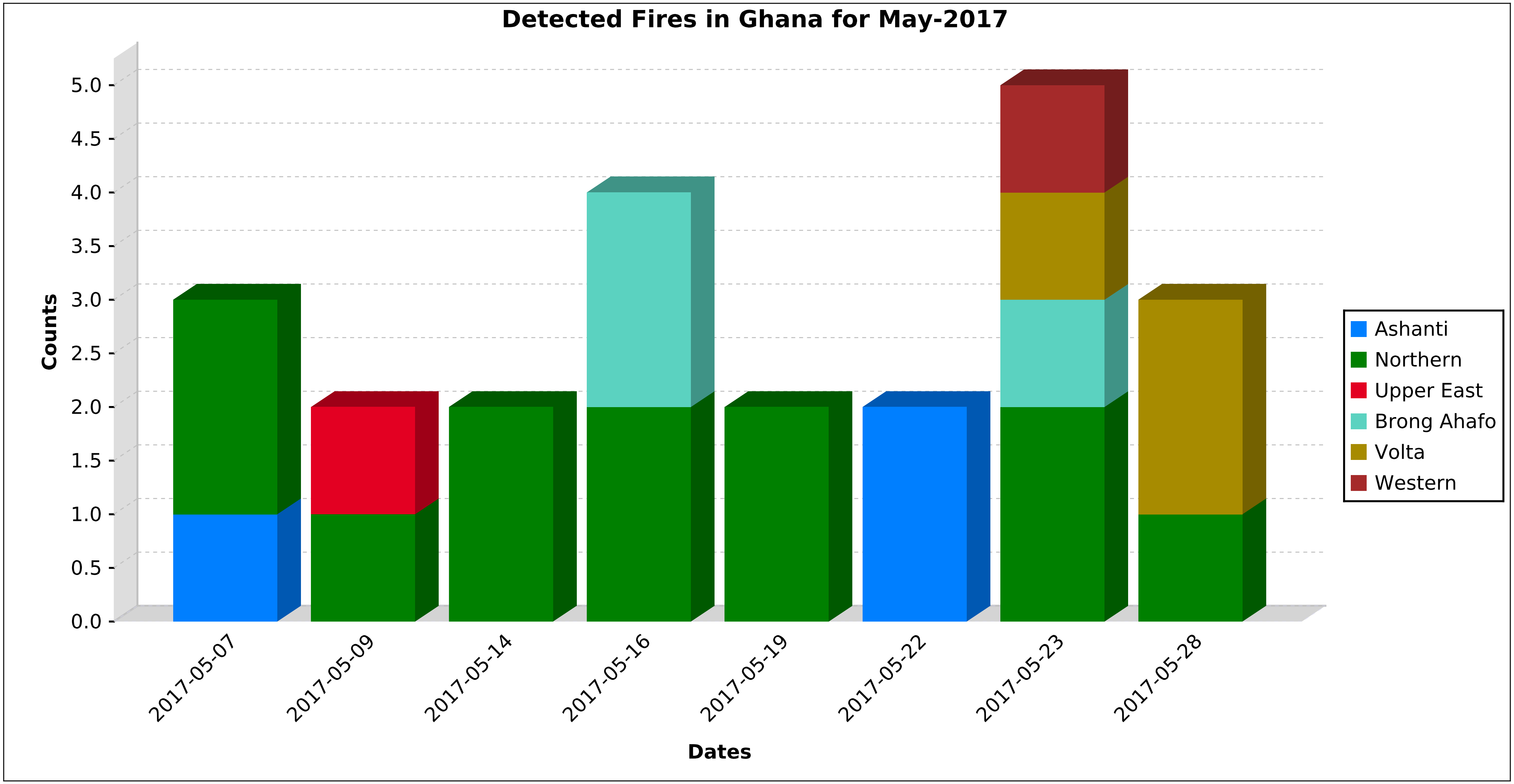

Daily Fire Detection count > 50 are highlighted in orange and Fire detection >=100 are highlighted in red.

Aug-2017		11/08/17	29/08/17	Monthly Total
**Brong Ahafo**	Kintampo South	1	0	**1**
	Nkoranza	0	1	**1**
	**Brong Ahafo Total**	1	1	**2**
**Provincial Total**		**1**	**1**	**2**

Daily Fire Detection count > 50 are highlighted in orange and Fire detection >=100 are highlighted in red.

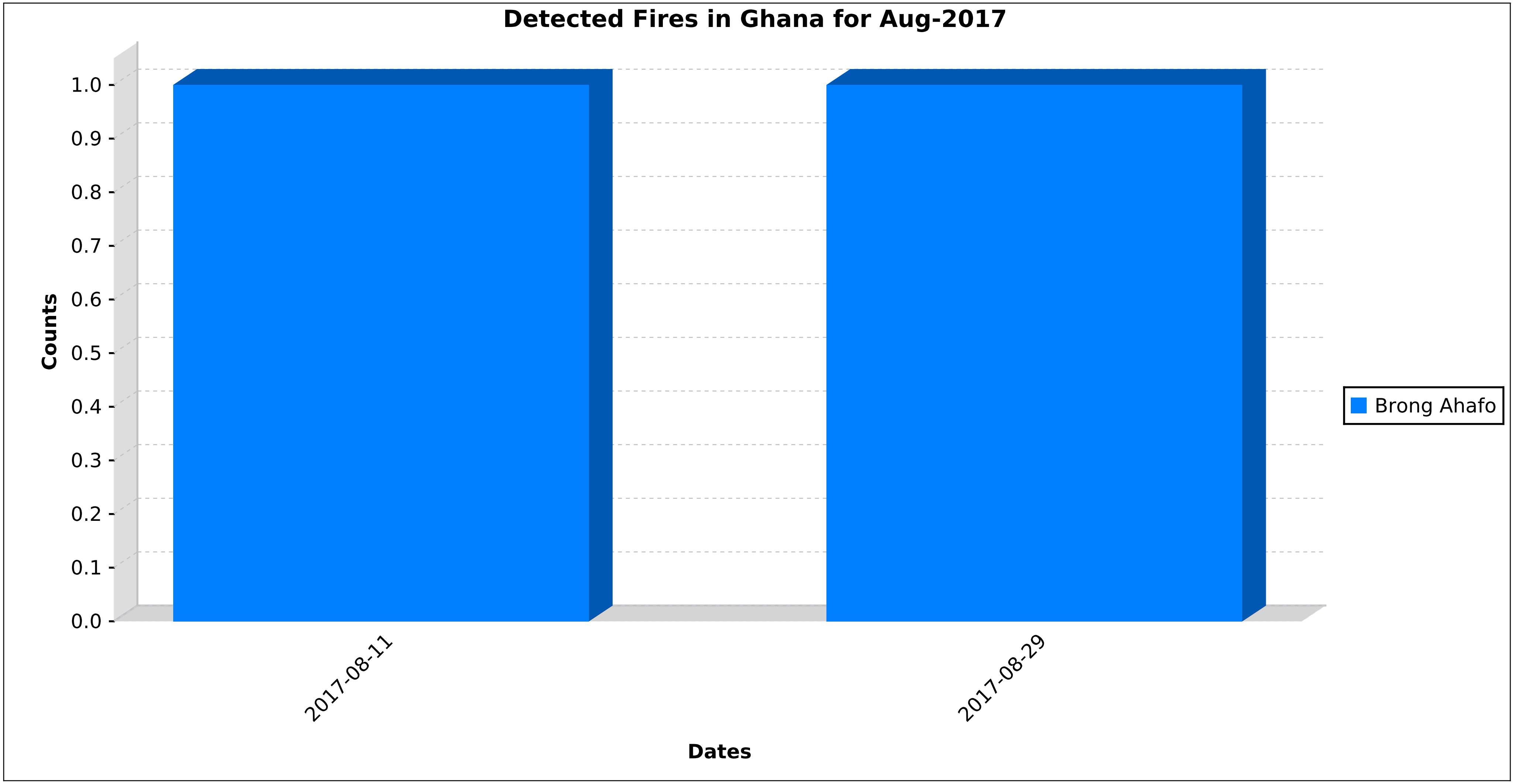

Daily Fire Detection count > 50 are highlighted in orange and Fire detection >=100 are highlighted in red.

Sep-2017		05/09/17	18/09/17	30/09/17	Monthly Total
**Central**	Gomoa	1	0	0	**1**
	**Central Total**	1	0	0	**1**
**Eastern**	Asuogyaman	1	0	0	**1**
	**Eastern Total**	1	0	0	**1**
**Greater Accra**	Dangbe East	0	1	0	**1**
	**Greater Accra Total**	0	1	0	**1**
**Volta**	Nkwanta	0	0	1	**1**
	**Volta Total**	0	0	1	**1**
**Provincial Total**		**2**	**1**	**1**	**4**

Daily Fire Detection count > 50 are highlighted in orange and Fire detection >=100 are highlighted in red.

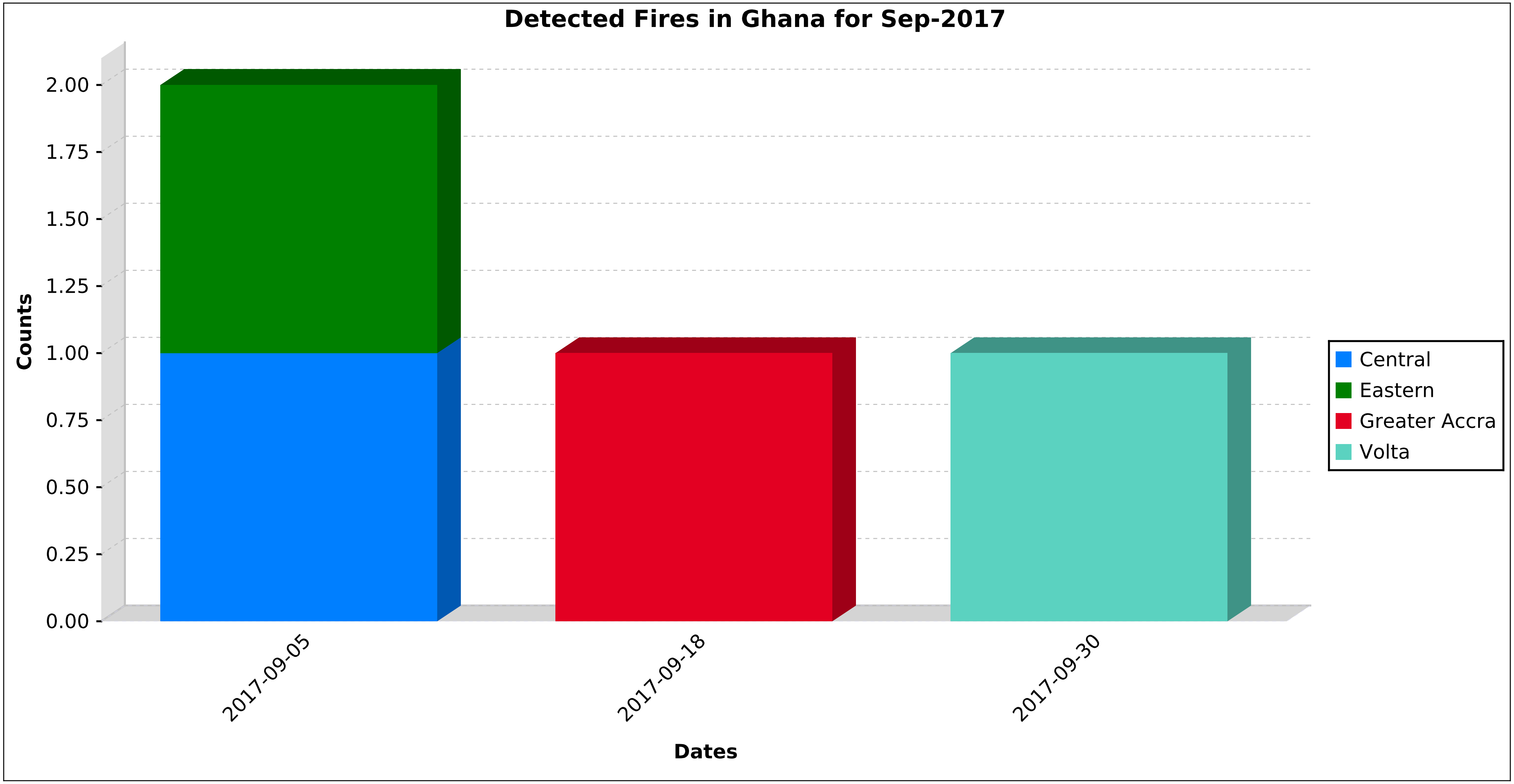

Daily Fire Detection count > 50 are highlighted in orange and Fire detection >=100 are highlighted in red.

Oct-2017		02/10/17	12/10/17	14/10/17	18/10/17	20/10/17	21/10/17	23/10/17	25/10/17	26/10/17	29/10/17	30/10/17	Monthly Total
**Brong Ahafo**	Kintampo North	0	0	0	0	0	0	0	1	0	0	0	**1**
	Kintampo South	0	0	0	0	0	0	0	0	0	0	2	**2**
	Techiman	0	0	0	0	0	0	0	1	0	0	0	**1**
	**Brong Ahafo Total**	0	0	0	0	0	0	0	2	0	0	2	**4**
**Eastern**	Akwapim North	1	0	0	0	0	0	0	0	0	0	0	**1**
	New Juaben	0	0	0	0	0	0	1	0	0	0	0	**1**
	**Eastern Total**	1	0	0	0	0	0	1	0	0	0	0	**2**
**Northern**	Bunkpurugu Yunyoo	0	0	0	0	2	0	0	0	0	0	0	**2**
	Central Gonja	0	0	0	0	0	0	1	0	0	0	3	**4**
	East Gonja	0	0	0	0	0	1	1	3	0	0	1	**6**
	East Mamprusi	0	0	0	0	0	0	0	0	1	1	0	**2**
	Gushiegu	0	0	0	0	0	0	1	5	2	0	2	**10**
	Karaga	0	0	0	0	0	0	0	0	0	0	1	**1**
	Nanumba North	0	0	0	0	0	0	2	5	0	0	3	**10**
	Nanumba South	0	0	1	0	0	2	1	1	0	0	0	**5**
	Saboba Chereponi	0	0	0	0	0	0	1	6	0	0	0	**7**
	Sawa-Tuna-Kalba	0	0	0	0	0	1	0	0	0	0	0	**1**
	West Mamprusi	0	0	0	0	0	2	0	0	3	0	3	**8**
	Yendi	0	0	1	1	0	0	0	3	0	0	1	**6**

Daily Fire Detection count > 50 are highlighted in orange and Fire detection >=100 are highlighted in red.

Oct-2017		02/10/17	12/10/17	14/10/17	18/10/17	20/10/17	21/10/17	23/10/17	25/10/17	26/10/17	29/10/17	30/10/17	Monthly Total
**Northern**	Zabzugu Tatale	0	0	0	0	0	0	2	0	0	0	0	**2**
	**Northern Total**	0	0	2	1	2	6	9	23	6	1	14	**64**
**Upper East**	Bawku West	0	0	0	0	0	0	0	0	0	0	1	**1**
	Bolgatanga	0	0	0	0	0	1	0	0	0	0	0	**1**
	Builsa	0	1	0	0	0	2	0	0	0	0	6	**9**
	Kassena Nankana	0	0	0	0	0	0	0	0	0	0	3	**3**
	Talensi Nabdam	0	0	0	0	0	0	0	1	0	2	1	**4**
	**Upper East Total**	0	1	0	0	0	3	0	1	0	2	11	**18**
**Upper West**	Lawra	0	0	0	0	0	1	0	2	1	0	2	**6**
	Sissala East	0	0	0	0	0	0	0	3	0	0	2	**5**
	Sissala West	0	0	0	0	0	0	0	0	0	0	2	**2**
	Wa West	0	0	0	0	0	0	0	0	0	0	1	**1**
	**Upper West Total**	0	0	0	0	0	1	0	5	1	0	7	**14**
**Volta**	Hohoe	0	0	0	0	0	0	0	1	0	0	0	**1**
	Krachi	0	0	0	0	0	1	1	2	0	0	0	**4**
	Krachi East	0	0	0	0	1	0	0	0	0	0	0	**1**
	Nkwanta	0	0	0	0	0	0	2	4	0	0	1	**7**
	**Volta Total**	0	0	0	0	1	1	3	7	0	0	1	**13**
**Provincial Total**		**1**	**1**	**2**	**1**	**3**	**11**	**13**	**38**	**7**	**3**	**35**	**115**

Daily Fire Detection count > 50 are highlighted in orange and Fire detection >=100 are highlighted in red.

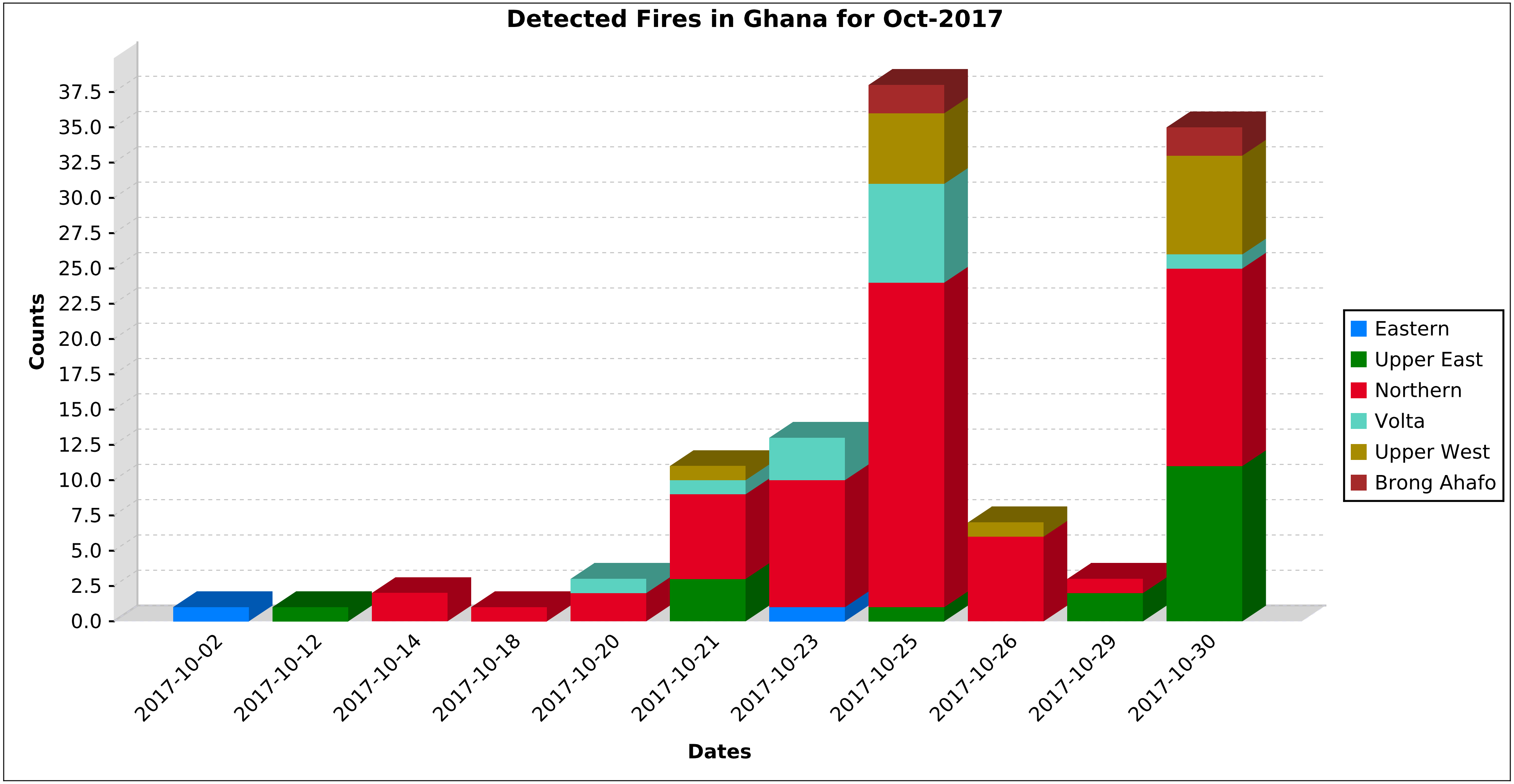

Daily Fire Detection count > 50 are highlighted in orange and Fire detection >=100 are highlighted in red.

Nov-2017		01/11/17	02/11/17	03/11/17	04/11/17	05/11/17	06/11/17	07/11/17	08/11/17	09/11/17	10/11/17	11/11/17	12/11/17	13/11/17	14/11/17	15/11/17	16/11/17	17/11/17	18/11/17	19/11/17	20/11/17	21/11/17	22/11/17	23/11/17	24/11/17	25/11/17	26/11/17	27/11/17	28/11/17	29/11/17	30/11/17	Monthly Total
**Ashanti**	Afigya Sekyere	0	0	0	0	0	0	0	1	0	0	0	0	0	0	0	0	0	0	0	0	0	0	0	0	0	0	0	0	0	0	**1**
	Asante Akim North	0	0	0	0	0	0	0	0	0	0	0	0	0	0	0	0	0	0	0	0	0	1	0	0	0	3	0	0	0	1	**5**
	Sekyere East	0	0	0	0	0	0	0	0	0	0	0	0	0	0	0	0	0	0	0	0	0	0	0	2	0	7	0	0	11	9	**29**
	Sekyere West	0	0	0	0	0	0	0	0	0	0	0	0	0	0	0	0	0	0	0	0	0	0	0	0	0	1	0	0	0	0	**1**
	**Ashanti Total**	0	0	0	0	0	0	0	1	0	0	0	0	0	0	0	0	0	0	0	0	0	1	0	2	0	11	0	0	11	10	**36**
**Brong Ahafo**	Atebubu-Amantin	1	0	0	0	0	0	0	2	0	0	0	0	0	0	0	0	2	0	0	0	0	0	0	0	0	4	1	1	0	0	**11**
	Jaman South	0	0	0	0	0	0	0	0	0	0	0	0	0	0	0	0	0	0	0	0	0	0	0	0	0	0	0	0	2	0	**2**
	Kintampo North	0	0	0	0	0	0	0	4	0	0	0	0	0	0	5	0	8	0	0	0	0	17	0	0	0	35	10	18	9	0	**106**
	Kintampo South	0	0	0	0	0	0	0	1	0	0	0	0	0	0	1	0	4	0	0	0	0	0	0	0	0	3	0	0	0	0	**9**
	Nkoranza	0	0	0	0	0	0	0	0	0	0	0	0	0	0	0	0	0	0	0	0	0	0	0	0	0	1	0	0	1	0	**2**
	Pru	1	0	1	0	0	0	0	6	0	2	0	0	0	0	5	0	11	0	6	1	0	6	0	0	0	38	14	12	21	0	**124**
	Sene	0	0	0	0	0	0	0	7	0	0	0	0	0	0	4	0	7	0	1	0	0	7	0	0	0	35	12	18	10	0	**101**
	Sunyani	0	0	0	0	0	0	0	0	0	0	0	0	0	0	0	0	0	0	0	0	0	0	0	0	0	0	0	0	1	0	**1**
	Tain	0	0	0	0	0	0	0	1	0	0	0	0	1	0	6	0	8	0	0	0	0	7	0	0	0	3	3	6	7	0	**42**
	**Brong Ahafo Total**	2	0	1	0	0	0	0	21	0	2	0	0	1	0	21	0	40	0	7	1	0	37	0	0	0	119	40	55	51	0	**398**
**Central**	Gomoa	0	0	0	0	0	0	0	0	0	0	0	0	0	0	0	0	0	0	0	0	0	0	0	0	0	3	0	0	0	0	**3**
	**Central Total**	0	0	0	0	0	0	0	0	0	0	0	0	0	0	0	0	0	0	0	0	0	0	0	0	0	3	0	0	0	0	**3**
**Eastern**	Afram Plains	0	0	0	0	0	0	0	4	0	0	0	0	4	0	1	0	1	0	0	0	0	0	0	0	0	13	2	6	3	0	**34**
	Fanteakwa	1	0	0	0	0	0	0	0	0	0	0	0	0	0	0	0	2	0	0	0	0	0	0	0	0	0	0	0	0	0	**3**

Daily Fire Detection count > 50 are highlighted in orange and Fire detection >=100 are highlighted in red.

Nov-2017		01/11/17	02/11/17	03/11/17	04/11/17	05/11/17	06/11/17	07/11/17	08/11/17	09/11/17	10/11/17	11/11/17	12/11/17	13/11/17	14/11/17	15/11/17	16/11/17	17/11/17	18/11/17	19/11/17	20/11/17	21/11/17	22/11/17	23/11/17	24/11/17	25/11/17	26/11/17	27/11/17	28/11/17	29/11/17	30/11/17	Monthly Total
**Eastern**	Kwahu South	0	0	0	0	0	0	0	0	0	1	0	0	0	0	0	0	0	0	0	0	0	0	0	0	0	0	0	0	2	0	**3**
	**Eastern Total**	1	0	0	0	0	0	0	4	0	1	0	0	4	0	1	0	3	0	0	0	0	0	0	0	0	13	2	6	5	0	**40**
**Greater Accra**	Dangbe West	0	0	0	0	0	0	0	0	0	0	0	1	0	0	0	0	0	0	0	0	0	0	0	0	0	0	0	0	0	0	**1**
	**Greater Accra Total**	0	0	0	0	0	0	0	0	0	0	0	1	0	0	0	0	0	0	0	0	0	0	0	0	0	0	0	0	0	0	**1**
**Northern**	Bole	0	0	0	0	0	0	0	4	1	0	0	0	1	0	13	0	20	5	1	0	0	17	0	1	2	60	23	20	18	1	**187**
	Bunkpurugu Yunyoo	0	0	1	1	0	1	0	13	0	6	0	4	0	0	1	0	11	0	0	0	0	0	0	2	1	8	1	14	1	0	**65**
	Central Gonja	0	0	0	0	0	0	0	36	0	6	28	2	5	1	11	0	30	6	3	2	0	57	0	7	0	177	66	61	79	3	**580**
	East Gonja	2	0	0	2	0	0	0	14	0	1	6	6	5	0	20	0	25	0	5	2	0	70	0	4	2	249	55	86	95	1	**650**
	East Mamprusi	0	3	0	1	0	1	0	13	0	0	2	6	2	0	18	0	8	0	1	0	0	2	0	0	0	15	6	3	4	0	**85**
	Gushiegu	1	1	3	6	1	0	0	13	2	4	6	1	0	3	6	5	6	0	0	0	0	0	0	1	0	46	18	21	17	2	**163**
	Karaga	0	0	1	4	0	3	0	18	0	1	5	1	4	1	17	0	9	0	2	0	2	1	0	3	0	41	9	25	11	1	**159**
	Nanumba North	1	0	3	0	0	0	0	4	0	0	0	0	0	0	1	0	0	0	0	0	0	1	0	0	0	26	16	22	21	1	**96**
	Nanumba South	0	0	1	1	0	0	0	3	0	1	0	0	0	0	0	0	0	0	0	0	0	4	0	0	0	15	3	2	5	1	**36**
	Saboba Chereponi	3	1	0	1	0	0	0	17	0	3	2	5	1	0	5	0	3	0	2	0	0	1	0	1	0	22	8	12	8	5	**100**
	Savelugu Nanton	1	0	0	2	0	0	0	17	0	2	0	2	1	0	11	0	4	0	1	0	4	4	0	1	0	27	5	5	2	0	**89**
	Sawa-Tuna-Kalba	0	0	0	0	0	0	0	44	0	0	34	1	3	0	50	0	80	7	2	3	0	98	1	12	4	148	89	66	84	2	**728**
	Tamale	0	0	0	0	0	0	0	4	0	0	0	0	0	0	0	0	2	0	0	0	0	1	0	2	0	8	0	10	4	0	**31**
	Tolon-Kumbungu	0	0	0	2	0	1	0	14	0	10	9	3	5	0	14	3	12	6	4	6	0	25	0	1	0	37	15	19	12	1	**199**
	West Gonja	0	0	0	0	0	1	0	17	0	2	4	0	4	0	28	0	31	12	5	5	0	87	0	2	1	145	101	116	87	3	**651**
	West Mamprusi	0	1	1	1	0	3	8	80	1	9	68	6	6	3	52	2	46	1	9	0	0	3	0	0	2	36	12	25	31	5	**411**

Daily Fire Detection count > 50 are highlighted in orange and Fire detection >=100 are highlighted in red.

Nov-2017		01/11/17	02/11/17	03/11/17	04/11/17	05/11/17	06/11/17	07/11/17	08/11/17	09/11/17	10/11/17	11/11/17	12/11/17	13/11/17	14/11/17	15/11/17	16/11/17	17/11/17	18/11/17	19/11/17	20/11/17	21/11/17	22/11/17	23/11/17	24/11/17	25/11/17	26/11/17	27/11/17	28/11/17	29/11/17	30/11/17	Monthly Total
**Northern**	Yendi	0	0	0	4	0	0	0	34	0	0	7	3	4	7	35	1	9	0	6	4	2	13	0	0	0	76	16	23	35	2	**281**
	Zabzugu Tatale	3	0	0	0	0	0	0	6	0	1	1	0	0	1	1	0	1	0	0	0	0	5	0	0	0	41	10	20	3	2	**95**
	**Northern Total**	11	6	10	25	1	10	8	351	4	46	172	40	41	16	283	11	297	37	41	22	8	389	1	37	12	1177	453	550	517	30	**4606**
**Upper East**	Bawku Municipal	0	0	0	0	0	0	0	0	0	0	0	0	0	0	0	0	5	0	2	0	0	0	0	0	0	2	0	2	0	0	**11**
	Bawku West	0	0	0	0	0	0	0	6	0	3	5	2	6	0	13	0	8	0	2	0	0	2	0	6	0	6	2	8	9	3	**81**
	Bolgatanga	0	0	0	1	0	0	0	5	0	0	1	0	1	0	14	0	3	0	0	0	0	0	0	3	0	0	5	3	0	0	**36**
	Bongo	0	0	0	0	0	0	0	0	0	0	0	0	0	0	4	0	0	0	1	0	0	0	0	0	0	0	1	5	1	0	**12**
	Builsa	0	4	4	3	0	7	0	68	0	3	18	3	9	1	43	3	33	0	4	0	0	5	0	0	4	25	6	8	2	0	**253**
	Garu Tempane	0	0	0	0	0	0	0	13	0	2	0	0	1	0	0	0	0	0	0	0	0	3	0	0	0	9	3	1	1	0	**33**
	Kassena Nankana	0	3	0	2	0	1	0	24	0	5	11	3	6	0	45	2	17	0	0	0	0	19	0	0	6	23	22	10	5	0	**204**
	Talensi Nabdam	0	0	0	0	0	0	1	19	2	5	5	2	1	0	15	0	18	0	0	0	0	1	0	2	0	8	6	15	0	0	**100**
	**Upper East Total**	0	7	4	6	0	8	1	135	2	18	40	10	24	1	134	5	84	0	9	0	0	30	0	11	10	73	45	52	18	3	**730**
**Upper West**	Jirapa Lambussie	1	0	0	3	0	1	0	34	0	0	11	3	13	1	18	0	13	1	1	0	0	3	0	0	1	3	7	7	10	0	**131**
	Lawra	0	0	0	1	0	0	1	13	0	0	2	0	6	0	9	2	4	0	0	0	0	2	0	0	0	0	1	2	6	0	**49**
	Nadowli	0	0	0	0	0	0	0	70	4	1	45	10	26	0	77	2	44	13	9	0	0	27	3	1	11	36	13	27	27	0	**446**
	Sissala East	1	4	0	1	0	7	0	62	0	1	27	21	13	7	100	1	34	3	2	0	0	14	0	5	7	58	32	28	45	6	**479**
	Sissala West	0	6	2	0	0	0	0	43	0	0	20	2	20	0	60	0	23	14	0	0	0	22	1	2	6	59	43	42	24	0	**389**
	Wa	0	0	0	0	0	0	0	10	0	0	6	2	7	0	21	0	24	6	2	0	0	40	0	5	5	57	15	32	39	0	**271**
	Wa East	0	0	0	0	0	0	0	23	0	0	19	2	11	1	56	0	40	5	2	1	0	56	0	5	0	72	25	38	39	3	**398**

Daily Fire Detection count > 50 are highlighted in orange and Fire detection >=100 are highlighted in red.

Nov-2017		01/11/17	02/11/17	03/11/17	04/11/17	05/11/17	06/11/17	07/11/17	08/11/17	09/11/17	10/11/17	11/11/17	12/11/17	13/11/17	14/11/17	15/11/17	16/11/17	17/11/17	18/11/17	19/11/17	20/11/17	21/11/17	22/11/17	23/11/17	24/11/17	25/11/17	26/11/17	27/11/17	28/11/17	29/11/17	30/11/17	Monthly Total
**Upper West**	Wa West	0	2	0	0	0	0	0	8	1	0	4	0	1	0	18	0	27	0	2	0	0	26	0	2	4	21	8	8	10	0	**142**
	**Upper West Total**	2	12	2	5	0	8	1	263	5	2	134	40	97	9	359	5	209	42	18	1	0	190	4	20	34	306	144	184	200	9	**2305**
**Volta**	Adaklu Anyigbe	0	0	0	0	0	0	0	0	0	0	0	0	0	0	0	0	0	0	0	0	0	0	0	0	0	0	0	4	0	0	**4**
	Ho	0	0	0	0	0	0	0	0	0	0	0	0	0	0	0	0	0	0	0	0	0	0	0	0	0	0	0	1	0	0	**1**
	Hohoe	0	0	0	0	0	0	0	0	0	0	0	0	0	0	0	0	1	0	0	0	0	0	0	0	0	1	0	0	0	0	**2**
	Jasikan	0	0	0	0	0	0	0	0	0	0	0	0	0	0	0	0	0	0	0	0	0	0	0	0	0	1	0	0	0	0	**1**
	Kpandu	0	0	0	0	0	0	0	0	0	0	0	0	0	0	0	0	0	0	0	0	0	0	0	0	0	1	0	0	0	0	**1**
	Krachi	0	0	1	0	0	0	0	1	0	0	0	0	0	0	1	0	2	0	0	0	0	0	0	0	0	5	0	1	2	0	**13**
	Krachi East	0	0	0	0	0	0	0	1	0	0	0	0	0	0	0	0	1	0	0	0	0	0	0	0	0	1	0	0	0	0	**3**
	Nkwanta	2	0	2	1	0	0	0	5	0	2	0	0	0	0	7	0	3	0	1	0	0	1	0	1	0	14	4	4	10	0	**57**
	**Volta Total**	2	0	3	1	0	0	0	7	0	2	0	0	0	0	8	0	7	0	1	0	0	1	0	1	0	23	4	10	12	0	**82**
**Provincial Total**		**18**	**25**	**20**	**37**	**1**	**26**	**10**	**782**	**11**	**71**	**346**	**91**	**167**	**26**	**806**	**21**	**640**	**79**	**76**	**24**	**8**	**648**	**5**	**71**	**56**	**1725**	**688**	**857**	**814**	**52**	**8201**

Daily Fire Detection count > 50 are highlighted in orange and Fire detection >=100 are highlighted in red.

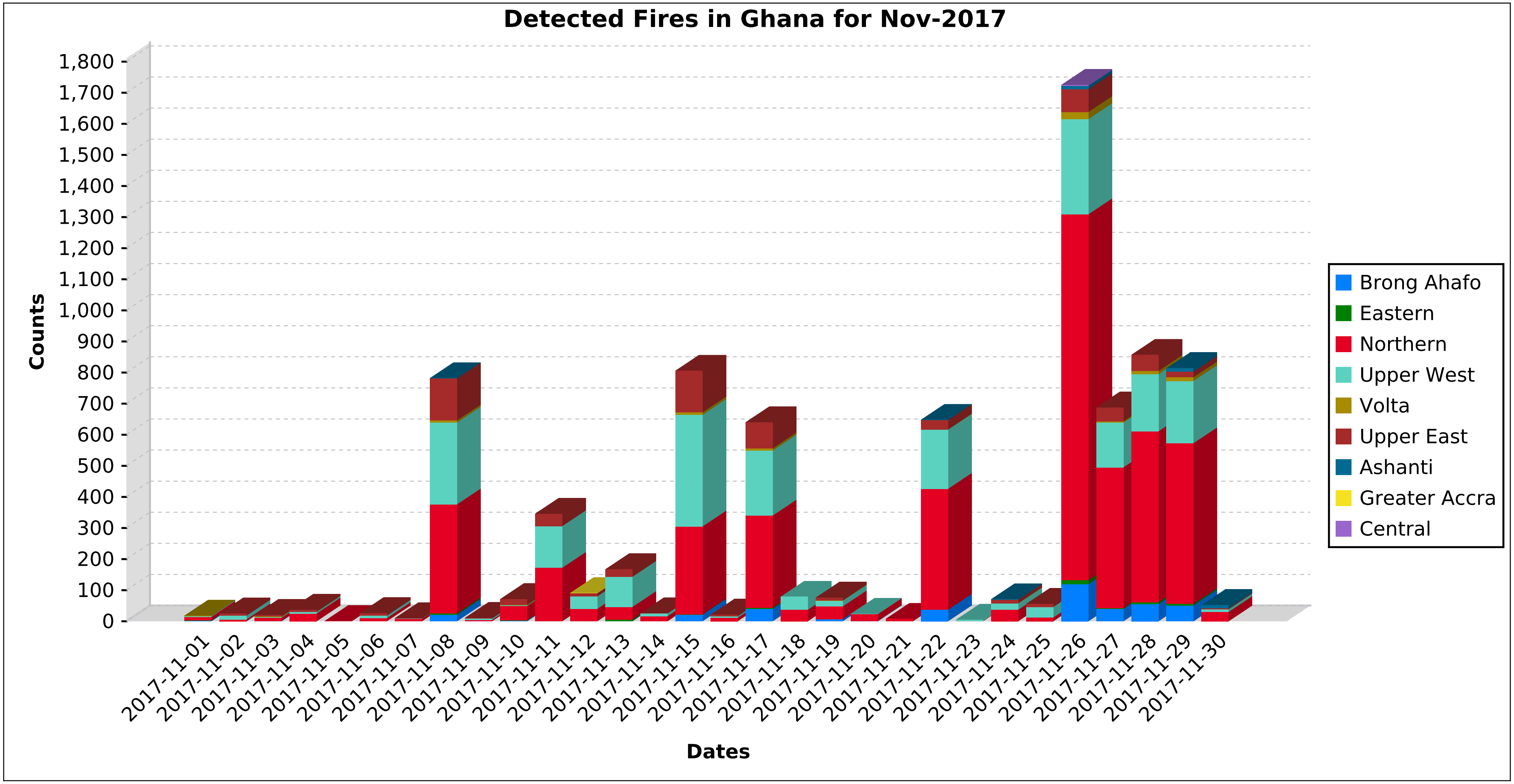

Daily Fire Detection count > 50 are highlighted in orange and Fire detection >=100 are highlighted in red.

Dec-2017		01/12/17	02/12/17	03/12/17	04/12/17	05/12/17	06/12/17	07/12/17	08/12/17	09/12/17	10/12/17	11/12/17	12/12/17	13/12/17	14/12/17	15/12/17	16/12/17	17/12/17	18/12/17	19/12/17	20/12/17	21/12/17	22/12/17	23/12/17	24/12/17	25/12/17	26/12/17	27/12/17	28/12/17	29/12/17	30/12/17	31/12/17	Monthly Total
**Ashanti**	Asante Akim North	1	0	0	0	0	0	0	0	0	10	0	5	0	2	0	0	1	0	1	0	0	0	0	3	2	20	0	4	0	0	6	**55**
	Asante Akim South	0	0	0	0	0	0	0	0	0	0	0	0	0	0	0	0	1	0	0	0	0	0	0	0	0	0	0	0	0	0	0	**1**
	Ejura Sekyedumas	0	0	0	0	0	0	0	0	0	0	0	0	0	0	0	0	0	0	0	0	0	0	0	1	0	1	0	0	0	0	1	**3**
	Offinso	0	0	0	0	0	0	0	0	0	0	0	0	0	0	0	0	0	0	0	0	0	0	2	1	0	3	0	0	0	0	0	**6**
	Sekyere East	8	1	1	2	0	4	0	1	3	40	0	14	11	3	7	0	23	1	1	0	0	8	7	25	0	38	0	49	1	1	41	**290**
	Sekyere West	0	0	0	0	0	0	0	0	0	5	0	8	1	3	0	0	10	0	0	0	0	0	0	8	0	17	0	15	0	1	11	**79**
	**Ashanti Total**	9	1	1	2	0	4	0	1	3	55	0	27	12	8	7	0	35	1	2	0	0	8	9	38	2	79	0	68	1	2	59	**434**
**Brong Ahafo**	Atebubu-Amantin	0	0	0	0	0	0	0	0	0	15	0	2	1	3	2	0	1	1	2	0	0	1	0	3	0	0	0	2	0	1	1	**35**
	Berekum	0	0	0	0	0	1	0	0	0	0	0	0	0	0	0	0	0	0	2	0	0	0	0	0	0	0	0	0	0	0	0	**3**
	Dormaa	0	0	0	0	0	0	0	0	0	1	0	0	0	0	0	0	0	0	0	0	0	0	0	0	0	0	0	3	0	1	9	**14**
	Jaman North	0	0	0	0	0	0	0	2	0	2	0	0	0	0	0	0	0	0	0	0	2	0	1	2	0	0	0	0	0	0	3	**12**
	Jaman South	0	0	0	0	0	0	0	0	0	0	0	0	0	0	0	0	0	0	0	0	0	0	0	0	0	1	0	0	0	0	0	**1**
	Kintampo North	7	0	1	7	6	9	0	17	1	128	0	16	11	12	19	29	55	1	0	0	11	19	25	108	0	34	5	66	6	7	74	**674**
	Kintampo South	4	0	0	2	0	2	0	1	0	19	0	2	1	1	2	3	15	0	0	0	0	4	4	59	0	45	3	29	5	0	48	**249**
	Nkoranza	0	0	0	0	0	0	2	4	0	31	0	2	6	0	7	1	18	0	1	0	0	7	2	53	1	18	2	26	7	4	29	**221**
	Pru	4	0	3	13	4	3	1	3	0	78	0	8	12	12	10	8	32	0	3	0	1	4	5	72	0	31	1	52	16	3	50	**429**
	Sene	17	4	0	0	4	1	1	12	0	108	0	8	9	21	8	14	44	0	4	0	0	2	9	55	2	80	0	91	7	9	102	**612**
	Tain	0	0	0	4	0	4	0	14	0	93	6	15	23	15	13	7	37	0	1	0	5	33	15	80	5	52	0	31	11	6	42	**512**

Daily Fire Detection count > 50 are highlighted in orange and Fire detection >=100 are highlighted in red.

Dec-2017		01/12/17	02/12/17	03/12/17	04/12/17	05/12/17	06/12/17	07/12/17	08/12/17	09/12/17	10/12/17	11/12/17	12/12/17	13/12/17	14/12/17	15/12/17	16/12/17	17/12/17	18/12/17	19/12/17	20/12/17	21/12/17	22/12/17	23/12/17	24/12/17	25/12/17	26/12/17	27/12/17	28/12/17	29/12/17	30/12/17	31/12/17	Monthly Total
**Brong Ahafo**	Techiman	0	0	0	0	0	0	0	0	0	2	0	0	0	0	0	0	1	0	0	0	0	1	0	0	0	0	0	0	0	0	1	**5**
	**Brong Ahafo Total**	32	4	4	26	14	20	4	53	1	477	6	53	63	64	61	62	203	2	13	0	19	71	61	432	8	261	11	300	52	31	359	**2767**
**Central**	Gomoa	0	0	0	0	0	0	0	0	0	0	0	0	0	0	0	0	0	0	0	0	0	0	0	0	0	1	0	0	0	0	0	**1**
	**Central Total**	0	0	0	0	0	0	0	0	0	0	0	0	0	0	0	0	0	0	0	0	0	0	0	0	0	1	0	0	0	0	0	**1**
**Eastern**	Afram Plains	10	2	0	0	2	0	0	1	0	12	0	10	0	5	2	2	10	1	0	0	0	0	4	41	0	61	0	34	6	0	35	**238**
	Asuogyaman	0	0	0	0	0	0	0	0	0	0	0	0	0	0	0	0	0	0	0	0	0	0	0	0	0	4	0	0	0	0	0	**4**
	Atiwa	0	0	0	0	0	0	0	0	0	0	0	0	0	0	0	0	0	0	0	0	0	0	0	0	0	1	0	0	0	0	0	**1**
	Fanteakwa	0	0	0	0	1	0	0	0	0	0	0	2	0	1	1	0	0	0	0	0	0	0	0	7	0	1	0	3	0	2	2	**20**
	Kwahu South	0	0	0	0	0	0	0	0	0	1	0	1	0	0	1	0	4	0	1	0	0	0	0	1	0	3	0	6	0	0	0	**18**
	**Eastern Total**	10	2	0	0	3	0	0	1	0	13	0	13	0	6	4	2	14	1	1	0	0	0	4	49	0	70	0	43	6	2	37	**281**
**Greater Accra**	Dangbe East	2	0	0	0	0	0	0	0	0	0	0	0	0	0	0	0	0	0	0	0	0	0	0	0	0	2	0	1	0	0	2	**7**
	Tema	0	0	0	0	0	0	0	0	0	0	0	0	0	0	0	0	0	0	0	0	0	0	0	0	0	0	0	0	0	0	1	**1**
	**Greater Accra Total**	2	0	0	0	0	0	0	0	0	0	0	0	0	0	0	0	0	0	0	0	0	0	0	0	0	2	0	1	0	0	3	**8**
**Northern**	Bole	13	1	6	17	1	22	0	32	3	227	1	39	28	8	29	3	94	1	3	0	5	18	9	100	2	44	5	50	9	9	32	**811**
	Bunkpurugu Yunyoo	7	4	1	0	0	2	0	2	0	5	0	0	0	2	0	2	12	7	5	0	0	1	0	5	0	2	3	16	0	1	0	**77**
	Central Gonja	32	6	20	20	12	16	5	45	8	352	11	41	36	17	33	43	130	10	12	0	7	20	11	106	0	97	5	62	18	16	59	**1250**
	East Gonja	36	25	11	3	10	7	6	28	8	370	3	20	26	43	30	45	122	14	6	0	5	3	45	117	5	152	9	153	22	34	102	**1460**
	East Mamprusi	0	0	3	2	4	0	0	3	0	7	0	0	0	1	1	7	17	3	3	0	1	2	0	3	0	1	0	6	1	0	0	**65**
	Gushiegu	21	4	3	0	1	3	0	3	2	31	0	0	0	2	1	16	29	15	8	0	12	10	15	40	0	28	3	30	28	19	28	**352**
	Karaga	13	7	5	1	2	8	6	19	2	62	1	1	0	4	7	7	63	15	9	0	2	2	2	29	3	40	0	21	2	4	28	**365**

Daily Fire Detection count > 50 are highlighted in orange and Fire detection >=100 are highlighted in red.

Dec-2017		01/12/17	02/12/17	03/12/17	04/12/17	05/12/17	06/12/17	07/12/17	08/12/17	09/12/17	10/12/17	11/12/17	12/12/17	13/12/17	14/12/17	15/12/17	16/12/17	17/12/17	18/12/17	19/12/17	20/12/17	21/12/17	22/12/17	23/12/17	24/12/17	25/12/17	26/12/17	27/12/17	28/12/17	29/12/17	30/12/17	31/12/17	Monthly Total
**Northern**	Nanumba North	10	8	6	2	11	8	0	1	1	77	3	2	3	15	1	11	27	11	2	0	0	1	2	23	0	45	0	36	0	4	15	**325**
	Nanumba South	1	0	2	0	2	0	0	0	3	17	0	1	3	4	0	1	6	5	0	0	0	0	3	6	0	8	0	22	2	1	7	**94**
	Saboba Chereponi	6	3	0	0	4	1	1	0	7	33	0	0	6	11	0	12	11	22	18	0	1	6	39	16	4	12	3	10	17	21	27	**291**
	Savelugu Nanton	4	9	7	2	4	3	1	7	1	47	2	6	1	3	13	4	31	2	16	0	1	0	17	24	1	16	1	18	3	9	20	**273**
	Sawa-Tuna-Kalba	48	19	28	26	3	26	6	44	0	84	0	17	1	0	31	3	60	1	4	0	0	0	4	27	0	14	4	12	13	1	11	**487**
	Tamale	2	0	0	0	0	0	2	2	1	8	1	2	1	0	0	9	4	3	2	0	0	0	0	6	0	1	0	1	2	0	1	**48**
	Tolon-Kumbungu	5	8	6	7	4	6	3	10	0	37	0	2	0	3	8	1	42	8	9	0	5	1	8	23	0	17	0	13	7	4	13	**250**
	West Gonja	58	10	27	68	23	46	12	107	9	249	5	82	29	7	47	65	220	10	23	7	11	9	31	116	3	109	15	87	48	7	77	**1617**
	West Mamprusi	14	5	13	16	3	3	6	18	0	40	1	3	0	4	8	14	49	6	10	0	3	4	10	29	0	20	1	31	3	7	46	**367**
	Yendi	28	18	2	2	16	3	0	4	4	68	0	6	1	18	1	10	47	10	6	0	8	2	16	25	2	38	1	36	5	8	49	**434**
	Zabzugu Tatale	20	5	0	0	2	1	0	4	1	21	0	3	0	18	0	7	23	2	3	0	5	3	15	38	7	24	1	47	3	12	14	**279**
	**Northern Total**	318	132	140	166	102	155	48	329	50	1735	28	225	135	160	210	260	987	145	139	7	66	82	227	733	27	668	51	651	183	157	529	**8845**
**Upper East**	Bawku Municipal	0	0	0	0	0	0	0	0	0	1	0	0	0	0	0	0	0	0	0	0	0	0	0	0	0	0	0	0	0	0	0	**1**
	Bawku West	3	0	0	0	3	0	3	0	0	0	0	0	0	0	0	0	1	0	0	0	1	0	0	0	0	1	0	1	0	0	0	**13**
	Bolgatanga	0	0	2	0	0	0	0	0	0	5	0	0	0	0	0	0	0	0	0	0	0	0	0	2	0	0	0	0	0	0	1	**10**
	Bongo	4	0	1	0	0	0	0	0	0	0	0	0	0	0	0	0	1	0	0	0	0	0	0	0	0	0	0	1	0	0	0	**7**
	Builsa	7	0	1	3	2	5	2	8	0	5	0	0	0	0	3	3	9	0	2	0	0	0	0	1	0	1	0	1	0	0	4	**57**
	Garu Tempane	2	0	0	0	0	0	0	1	0	0	0	0	0	0	0	2	0	0	0	0	0	0	0	0	0	0	0	1	0	0	3	**9**
	Kassena Nankana	6	0	4	4	0	2	0	23	0	10	0	0	0	0	5	2	7	0	4	0	0	0	0	2	0	0	0	2	0	1	1	**73**

Daily Fire Detection count > 50 are highlighted in orange and Fire detection >=100 are highlighted in red.

Dec-2017		01/12/17	02/12/17	03/12/17	04/12/17	05/12/17	06/12/17	07/12/17	08/12/17	09/12/17	10/12/17	11/12/17	12/12/17	13/12/17	14/12/17	15/12/17	16/12/17	17/12/17	18/12/17	19/12/17	20/12/17	21/12/17	22/12/17	23/12/17	24/12/17	25/12/17	26/12/17	27/12/17	28/12/17	29/12/17	30/12/17	31/12/17	Monthly Total
**Upper East**	Talensi Nabdam	2	0	1	0	0	0	0	0	0	4	0	0	0	0	0	0	1	0	0	1	0	3	0	0	0	2	0	2	0	0	0	**16**
	**Upper East Total**	24	0	9	7	5	7	5	32	0	25	0	0	0	0	8	7	19	0	6	1	1	3	0	5	0	4	0	8	0	1	9	**186**
**Upper West**	Jirapa Lambussie	5	2	0	1	1	0	0	3	0	6	0	1	0	0	2	0	3	0	1	0	0	0	0	1	0	1	0	0	3	0	0	**30**
	Lawra	0	0	2	0	0	0	0	1	0	4	0	1	0	0	0	0	2	0	0	0	0	0	0	2	0	0	0	0	0	0	0	**12**
	Nadowli	8	2	3	4	1	3	1	11	0	10	0	0	0	4	4	2	15	0	2	0	0	0	0	1	0	2	0	2	0	3	8	**86**
	Sissala East	24	3	15	19	7	8	10	33	0	50	0	6	0	1	21	11	51	1	24	0	1	0	1	5	0	23	0	13	0	0	19	**346**
	Sissala West	11	21	11	16	6	5	4	16	0	44	0	7	0	0	19	0	31	0	8	0	1	3	0	4	0	14	0	10	1	0	5	**237**
	Wa	14	1	4	11	2	8	0	11	0	35	0	1	1	0	8	0	18	0	1	0	0	1	0	4	0	1	0	5	0	0	0	**126**
	Wa East	27	4	15	18	4	11	3	14	0	45	0	2	1	0	3	3	30	0	3	0	0	0	4	9	0	5	1	10	5	2	10	**229**
	Wa West	8	0	5	0	0	1	0	6	0	7	0	0	0	0	1	0	1	0	0	0	0	0	0	1	0	3	0	0	0	0	1	**34**
	**Upper West Total**	97	33	55	69	21	36	18	95	0	201	0	18	2	5	58	16	151	1	39	0	2	4	5	27	0	49	1	40	9	5	43	**1100**
**Volta**	Adaklu Anyigbe	1	0	0	0	0	0	0	1	0	0	0	0	0	0	0	0	0	0	1	0	0	0	1	5	0	10	0	29	4	0	7	**59**
	Akatsi	0	0	0	0	0	0	0	0	0	0	0	0	0	0	0	0	0	0	0	0	0	0	0	1	0	2	0	1	0	0	0	**4**
	Ho	0	0	1	0	0	0	0	2	0	0	0	0	0	0	0	0	0	0	0	0	0	0	0	0	0	7	0	3	0	0	2	**15**
	Jasikan	0	0	0	0	0	0	0	0	0	0	0	0	0	0	0	0	0	0	0	0	0	0	0	0	0	3	0	0	0	0	0	**3**
	Kadjebi	0	0	0	0	0	0	0	0	0	0	0	0	0	0	0	0	0	0	0	0	0	0	0	0	0	0	1	0	0	0	0	**1**
	Keta	0	0	0	0	0	0	0	0	0	0	0	0	0	0	0	0	0	0	0	0	0	0	0	0	0	0	0	2	0	0	0	**2**
	Ketu	0	0	0	0	0	0	0	0	0	0	0	0	0	0	1	0	0	0	0	0	0	0	0	0	0	0	0	0	0	0	0	**1**
	Kpandu	0	0	0	0	0	0	0	0	0	0	0	0	0	0	0	0	0	0	0	0	0	0	0	0	0	0	0	1	0	0	0	**1**
	Krachi	2	0	0	0	0	0	0	0	0	16	0	10	0	4	0	0	4	2	0	0	0	0	3	11	0	13	0	11	0	2	26	**104**

Daily Fire Detection count > 50 are highlighted in orange and Fire detection >=100 are highlighted in red.

Dec-2017		01/12/17	02/12/17	03/12/17	04/12/17	05/12/17	06/12/17	07/12/17	08/12/17	09/12/17	10/12/17	11/12/17	12/12/17	13/12/17	14/12/17	15/12/17	16/12/17	17/12/17	18/12/17	19/12/17	20/12/17	21/12/17	22/12/17	23/12/17	24/12/17	25/12/17	26/12/17	27/12/17	28/12/17	29/12/17	30/12/17	31/12/17	Monthly Total
**Volta**	Krachi East	0	0	0	0	1	0	0	0	0	3	0	1	0	0	0	0	0	0	0	0	0	0	0	1	0	5	0	9	0	0	11	**31**
	Nkwanta	13	0	4	0	1	1	0	5	0	21	0	4	0	9	4	9	5	0	0	0	0	2	0	10	0	34	0	40	9	9	29	**209**
	North Tongu	0	0	0	0	0	0	0	0	0	0	0	2	0	1	0	0	1	0	0	0	0	0	0	0	0	4	0	1	1	0	2	**12**
	South Dayi	0	0	0	0	0	0	0	0	0	0	0	0	0	0	0	0	1	0	0	0	0	0	0	0	0	0	0	0	0	0	0	**1**
	South Tongu	0	0	0	0	0	0	0	0	0	0	0	0	0	0	0	0	0	0	0	0	0	0	0	0	0	0	0	1	0	0	0	**1**
	**Volta Total**	16	0	5	0	2	1	0	8	0	40	0	17	0	14	5	9	11	2	1	0	0	2	4	28	0	78	1	98	14	11	77	**444**
**Western**	Mpohor Wassa East	0	0	0	0	0	0	0	0	0	0	0	0	0	0	0	0	0	0	0	0	0	0	0	0	0	3	0	0	0	0	0	**3**
	**Western Total**	0	0	0	0	0	0	0	0	0	0	0	0	0	0	0	0	0	0	0	0	0	0	0	0	0	3	0	0	0	0	0	**3**
**Provincial Total**		**508**	**172**	**214**	**270**	**147**	**223**	**75**	**519**	**54**	**2546**	**34**	**353**	**212**	**257**	**353**	**356**	**1420**	**152**	**201**	**8**	**88**	**170**	**310**	**1312**	**37**	**1215**	**64**	**1209**	**265**	**209**	**1116**	**14069**

Daily Fire Detection count > 50 are highlighted in orange and Fire detection >=100 are highlighted in red.

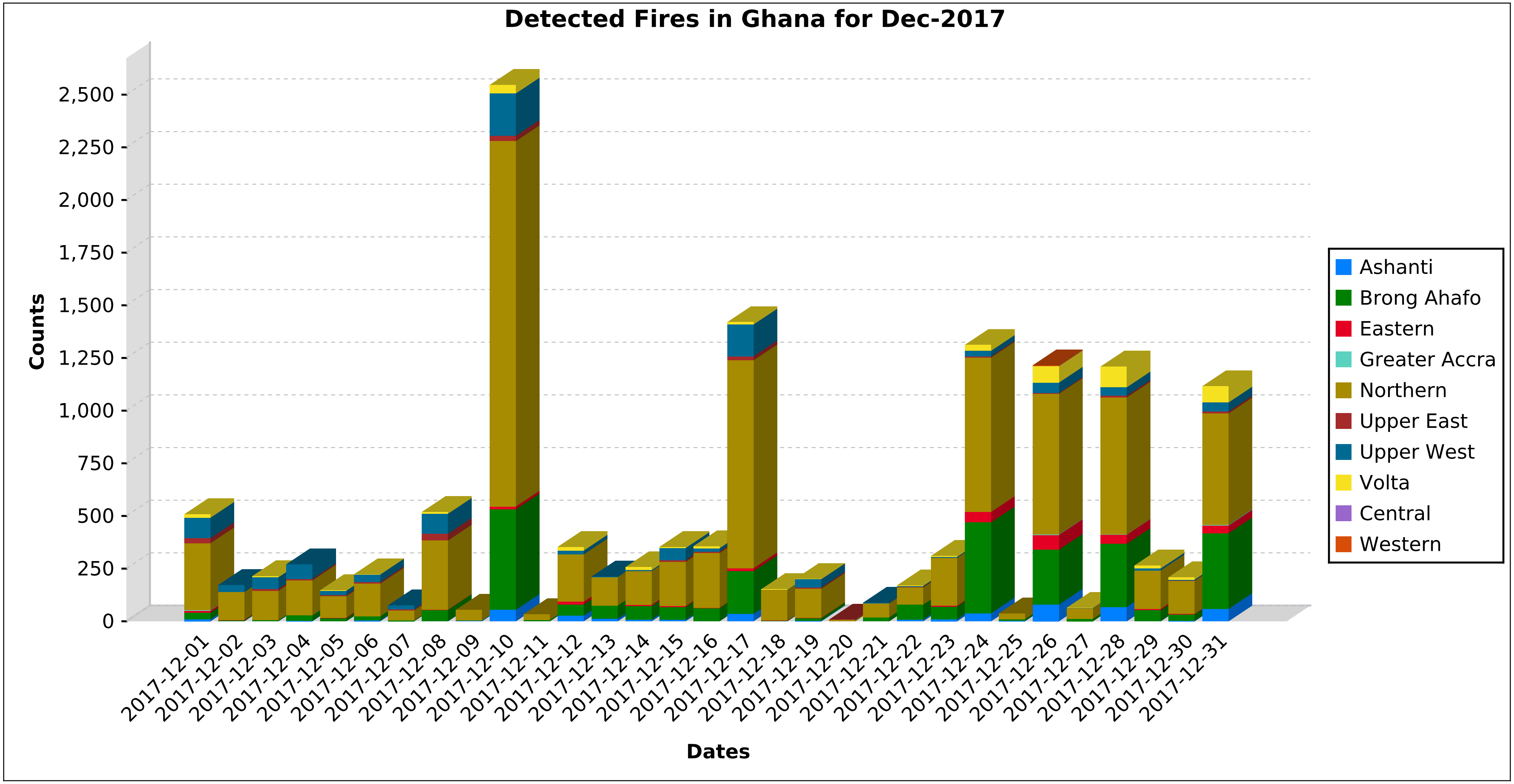

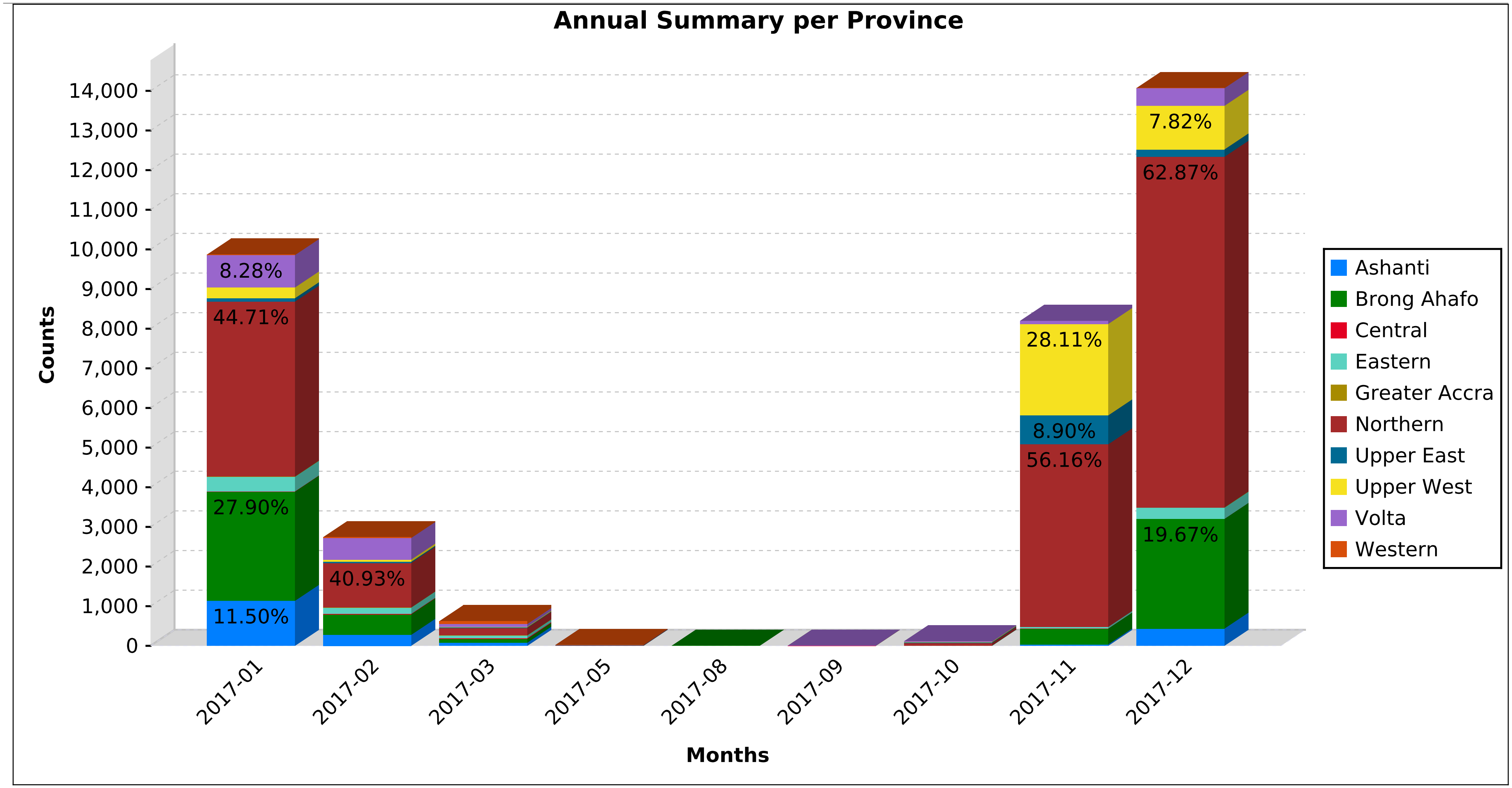

Contacts Us

Names	Email	Number
Philip Frost	pfrost@csir.co.za	+27 12 841 4665
Ndumiso Booi	nbooi@csir.co.za	+27 12 841 7760
