# Supplementary material for: Fire use practices, knowledge and perceptions in a West African savanna parkland
Source: PLoS One. 2022 May 19;17(5):e0240271. doi: 10.1371/journal.pone.0240271 (PMC9119518; doi:10.1371/journal.pone.0240271)
Supplement: S1 File — (PDF) [file pone.0240271.s003.pdf]

# Fire Detections

## Ghana

afis Advanced Fire Information System

## Detected Fires between Jan-2016 & Dec-2016

*Daily Fire Detection count > 50 are highlighted in orange and Fire detection >=100 are highlighted in red*

| Jan-2016 | | 01/01/16 | 02/01/16 | 03/01/16 | 04/01/16 | 05/01/16 | 06/01/16 | 07/01/16 | 08/01/16 | 09/01/16 | 10/01/16 | 11/01/16 | 12/01/16 | 13/01/16 | 14/01/16 | 15/01/16 | 16/01/16 | 17/01/16 | 18/01/16 | 19/01/16 | 20/01/16 | 21/01/16 | 22/01/16 | 23/01/16 | 24/01/16 | 25/01/16 | 26/01/16 | 27/01/16 | 28/01/16 | 29/01/16 | 30/01/16 | 31/01/16 | Monthly Total |
|---|---|---|---|---|---|---|---|---|---|---|---|---|---|---|---|---|---|---|---|---|---|---|---|---|---|---|---|---|---|---|---|---|---|
| **Ashanti** | Adansi North | 0 | 0 | 0 | 0 | 0 | 1 | 0 | 6 | 0 | 1 | 2 | 0 | 0 | 0 | 0 | 0 | 0 | 0 | 0 | 0 | 0 | 0 | 0 | 0 | 0 | 0 | 0 | 0 | 0 | 0 | 4 | **14** |
| | Adansi South | 0 | 0 | 0 | 0 | 0 | 3 | 0 | 0 | 0 | 0 | 0 | 0 | 0 | 0 | 0 | 0 | 0 | 0 | 0 | 0 | 0 | 0 | 0 | 2 | 0 | 2 | 4 | 0 | 4 | 0 | 1 | **16** |
| | Afigya Sekyere | 0 | 1 | 0 | 0 | 0 | 5 | 0 | 9 | 0 | 0 | 0 | 0 | 0 | 0 | 0 | | 0 | | 0 | 0 | 0 | 0 | 0 | 7 | 0 | 0 | 1 | 0 | 8 | 0 | 3 | **34** |
| | Ahafo Ano North | 0 | 0 | 0 | 0 | 0 | 0 | 0 | 0 | 0 | 0 | 0 | 0 | 0 | 0 | 0 | 0 | 0 | 0 | 0 | 0 | 0 | 0 | 0 | 0 | 0 | 0 | 0 | 0 | 1 | 0 | 0 | **1** |
| | Ahafo Ano South | 0 | 0 | 0 | 0 | 0 | 2 | 0 | 7 | 8 | 0 | 0 | 0 | 0 | 0 | | | 0 | 0 | 0 | 0 | 0 | 0 | 2 | 14 | 0 | 18 | 9 | 14 | 29 | 8 | 21 | **133** |
| | Amansie Central | 0 | 0 | 0 | 1 | 0 | 6 | 0 | 6 | 0 | 0 | 0 | 0 | 0 | 0 | 0 | 0 | 0 | 0 | 0 | 0 | 0 | 0 | 0 | 0 | 0 | 0 | 2 | 0 | 2 | 0 | 0 | **17** |
| | Amansie East | 0 | 0 | 0 | 2 | 0 | 2 | 0 | 8 | 4 | 0 | 0 | 0 | 0 | 0 | 5 | 0 | 2 | 0 | 0 | 0 | 0 | 4 | 0 | 1 | 0 | 2 | 0 | 0 | 10 | 3 | 7 | **50** |
| | Amansie West | 0 | 0 | 0 | 2 | 0 | 6 | 0 | 2 | 0 | 0 | 0 | 0 | 0 | 0 | 0 | 0 | 0 | 0 | 0 | 0 | 0 | 0 | 0 | 0 | 0 | 0 | 0 | 0 | 18 | 6 | 0 | **34** |
| | Asante Akim North | 11 | 1 | 0 | 20 | 2 | 102 | 10 | 6 | 12 | 4 | 7 | 3 | 0 | 0 | 3 | 0 | 2 | 0 | 0 | 0 | 0 | 1 | 0 | 4 | 0 | 7 | 6 | 5 | 10 | 0 | 13 | **259** |
| | Asante Akim South | 0 | 0 | 0 | 0 | 0 | 14 | 1 | 23 | 5 | 0 | 0 | 0 | 0 | 0 | 0 | 0 | 0 | 0 | 0 | 0 | 0 | 2 | 0 | 2 | 0 | 0 | 0 | 0 | 3 | 0 | 0 | **50** |
| | Atwima | 2 | 0 | 0 | 0 | | 0 | | 3 | 0 | 0 | 1 | 0 | 0 | 0 | 0 | 0 | 1 | 0 | 0 | 0 | 0 | 2 | 0 | 2 | 0 | 0 | 0 | 0 | 1 | 0 | 4 | **16** |
| | Atwima Mponua | 0 | 0 | 0 | 0 | 0 | 6 | 6 | 15 | 1 | 0 | 0 | 0 | 0 | 0 | 1 | 0 | 0 | 0 | 0 | 0 | 0 | 1 | 0 | 4 | 0 | 5 | 4 | 14 | 28 | 2 | 8 | **95** |
| | Bosomtwe-Kwanwoma | 0 | | 0 | 0 | 0 | 8 | 0 | 7 | 0 | 0 | 0 | 0 | 0 | 0 | 0 | 0 | 0 | 0 | 0 | 0 | 0 | 1 | 0 | 0 | 0 | 4 | 2 | 0 | 10 | 1 | 8 | **41** |
| | Ejisu-Juabeng | 0 | 0 | 0 | 3 | 0 | 3 | 0 | 2 | 0 | 0 | 1 | 0 | 0 | 0 | 3 | 0 | 0 | 0 | 0 | 0 | 0 | 1 | 0 | 0 | 0 | 0 | 0 | 0 | 2 | 0 | 2 | **17** |
| | Ejura Sekyedumas | 5 | 0 | 0 | 26 | 0 | 27 | 6 | 4 | 1 | 1 | 1 | 0 | 3 | 3 | 9 | 6 | 8 | 0 | 3 | 5 | 0 | 25 | 3 | 52 | 1 | 6 | 14 | 1 | 19 | 2 | 17 | **248** |
| | Kumasi | 0 | 0 | 0 | 0 | 0 | 8 | 0 | 2 | 0 | 0 | 0 | 0 | 1 | 0 | 0 | 0 | 0 | 0 | 0 | 0 | 0 | 0 | 0 | 0 | 0 | 0 | 0 | 0 | 0 | 0 | 0 | **11** |

EORIC - UENR copy

*Daily Fire Detection count > 50 are highlighted in orange and Fire detection >=100 are highlighted in red.*

| Jan-2016 | | 01/01/16 | 02/01/16 | 03/01/16 | 04/01/16 | 05/01/16 | 06/01/16 | 07/01/16 | 08/01/16 | 09/01/16 | 10/01/16 | 11/01/16 | 12/01/16 | 13/01/16 | 14/01/16 | 15/01/16 | 16/01/16 | 17/01/16 | 18/01/16 | 19/01/16 | 20/01/16 | 21/01/16 | 22/01/16 | 23/01/16 | 24/01/16 | 5/01/16 | 26/01/16 | 27/01/16 | 28/01/16 | 29/01/16 | 30/01/16 | 31/01/16 | Monthly Total |
|---|---|---|---|---|---|---|---|---|---|---|---|---|---|---|---|---|---|---|---|---|---|---|---|---|---|---|---|---|---|---|---|---|---|
| **Ashanti** | Kwabre | 1 | 0 | 0 | 0 | 0 | 4 | 0 | 4 | 0 | 0 | 0 | 0 | 0 | 0 | 0 | 0 | 0 | 0 | 0 | 0 | 0 | 0 | 0 | 1 | 0 | 0 | 0 | 0 | 1 | 0 | 0 | **11** |
| | Obuasi Municipal | 0 | 0 | 0 | 0 | 0 | 1 | 0 | 0 | 0 | 0 | 0 | 0 | 0 | 0 | 0 | 0 | 0 | 0 | 0 | 0 | 0 | 0 | 0 | 0 | 0 | 0 | 0 | 0 | 0 | 0 | 2 | **3** |
| | Offinso | 1 | 1 | 0 | 7 | 3 | 10 | 0 | 12 | 0 | 0 | 0 | 0 | 0 | 0 | 2 | 0 | 0 | 1 | 0 | 1 | [illegible] | 6 | [illegible] | 6 | 0 | 7 | 6 | 1 | 13 | 3 | 15 | **98** |
| | Sekyere East | 69 | 12 | 16 | 76 | 12 | 279 | 17 | 144 | 10 | 20 | 8 | 8 | 20 | 13 | 18 | 8 | 49 | 19 | 3 | 3 | 0 | 2 | 0 | 37 | 0 | 8 | 9 | 11 | 36 | 3 | 15 | **948** |
| | Sekyere West | 26 | 2 | 22 | 68 | 8 | 218 | 16 | 127 | 8 | 4 | 10 | 3 | 13 | 0 | 22 | 7 | 11 | 2 | 1 | 7 | 0 | 17 | 0 | 55 | 0 | 19 | 4 | 4 | 36 | 2 | 20 | **732** |
| | **Ashanti Total** | 115 | 17 | 38 | 205 | 25 | 705 | 56 | 417 | 49 | 30 | 30 | 14 | 37 | 16 | 64 | 21 | 73 | 22 | [illegible] | 16 | 0 | 85 | 8 | 187 | 1 | 78 | 61 | 50 | 231 | 30 | 140 | **2828** |
| **Brong Ahafo** | Asunafo North | 0 | 0 | 0 | 0 | 0 | 0 | 0 | 1 | 1 | 0 | 0 | 0 | 0 | 0 | 0 | 0 | 0 | 0 | 0 | 0 | 0 | 0 | 0 | 0 | 1 | 2 | 1 | 8 | 4 | 1 | 4 | **23** |
| | Asunafo South | 0 | 0 | 0 | 0 | 0 | 3 | 0 | 5 | 4 | 0 | 0 | 0 | 0 | 0 | [illegible] | 0 | 0 | 0 | 0 | 0 | 0 | 0 | 0 | 2 | 1 | 3 | 0 | 2 | 19 | 0 | 8 | **47** |
| | Asutifi | 0 | 0 | 0 | 0 | 0 | 3 | 0 | 1 | 0 | 0 | 0 | 0 | 0 | [illegible] | 2 | 0 | 0 | 0 | 0 | 0 | 0 | 0 | 0 | 2 | 0 | 1 | 1 | 1 | 13 | 0 | 5 | **29** |
| | Atebubu-Amantin | 17 | 0 | 13 | 37 | 1 | 128 | 0 | 9 | 2 | 3 | 10 | 1 | 7 | 0 | 14 | 5 | 8 | 6 | 1 | 6 | 0 | 17 | 0 | 39 | 1 | 8 | 7 | 5 | 4 | 1 | 10 | **360** |
| | Berekum | 7 | 0 | 0 | 2 | 0 | 3 | 0 | 1 | 0 | 0 | 1 | 0 | 0 | 0 | 1 | 1 | 2 | 13 | 2 | 5 | 0 | 3 | 6 | 14 | 1 | 8 | 26 | 10 | 34 | 0 | 13 | **153** |
| | Dormaa | 1 | 3 | 0 | 4 | 0 | 1 | 0 | 4 | 0 | 0 | 0 | 0 | 0 | 0 | 0 | 0 | 0 | 0 | 0 | 0 | 0 | 2 | 0 | 1 | 0 | 0 | 0 | 0 | 6 | 0 | 5 | **27** |
| | Jaman North | 0 | 0 | 0 | 0 | 0 | 0 | 0 | 0 | 0 | 0 | 0 | 0 | 0 | 0 | 0 | 0 | 0 | 0 | 0 | 0 | 0 | 0 | 0 | 0 | 0 | 1 | 0 | 0 | 0 | 0 | 0 | **1** |
| | Jaman South | 0 | 0 | 0 | 0 | 0 | 0 | 0 | [illegible] | 0 | 0 | 0 | 0 | 0 | 0 | 0 | 0 | 0 | 0 | 0 | 0 | 0 | 0 | 2 | 5 | 0 | 0 | 0 | 0 | 1 | 0 | 0 | **8** |
| | Kintampo North | 33 | 6 | 6 | 22 | 1 | 1[illegible]5 | 0 | 13 | 4 | 1 | 17 | 6 | 13 | 0 | 18 | 1 | 8 | 3 | 0 | 7 | 1 | 24 | 0 | 1 | 0 | 0 | 2 | 2 | 6 | 0 | 12 | **312** |
| | Kintampo South | 7 | 0 | 9 | 9 | 3 | 79 | 0 | 18 | 13 | 7 | 7 | 1 | 4 | 0 | 2 | 1 | 1 | 2 | 0 | 2 | 0 | 28 | 4 | 10 | 0 | 2 | 4 | 8 | 10 | 0 | 12 | **243** |
| | Nkoranza | 12 | 0 | 5 | 9 | [illegible] | 94 | 1 | 10 | 2 | 3 | 12 | 1 | 2 | 0 | 6 | 1 | 3 | 0 | 0 | 4 | 0 | 17 | 2 | 29 | 0 | 15 | 7 | 4 | 25 | 1 | 17 | **297** |
| | Pru | 18 | 0 | 2 | 37 | 2 | 53 | 0 | 18 | 6 | 3 | 15 | 7 | 10 | 0 | 5 | 2 | 6 | 3 | 1 | 8 | 0 | 7 | 0 | 4 | 0 | 5 | 0 | 2 | 4 | 0 | 6 | **224** |
| | Sene | 90 | 14 | 16 | 67 | 15 | 234 | 2 | 128 | 41 | 25 | 13 | 12 | 30 | 5 | 27 | 4 | 16 | 4 | 9 | 7 | 4 | 45 | 2 | 55 | 0 | 35 | 8 | 10 | 24 | 1 | 21 | **964** |
| | Sunyani | 1 | 0 | 0 | 3 | 0 | 4 | 0 | 9 | 1 | 0 | 0 | 0 | 0 | 0 | 0 | 0 | 0 | 0 | 0 | 1 | 0 | 7 | 11 | 6 | 0 | 5 | 10 | 9 | 28 | 5 | 16 | **116** |

EORIC - UENR copy

*Daily Fire Detection count > 50 are highlighted in orange and Fire detection >=100 are highlighted in red.*

| Jan-2016 | | 01/01/16 | 02/01/16 | 03/01/16 | 04/01/16 | 05/01/16 | 06/01/16 | 07/01/16 | 08/01/16 | 09/01/16 | 10/01/16 | 11/01/16 | 12/01/16 | 13/01/16 | 14/01/16 | 15/01/16 | 16/01/16 | 17/01/16 | 18/01/16 | 19/01/16 | 20/01/16 | 21/01/16 | 22/01/16 | 23/01/16 | 24/01/16 | 5/01/16 | 26/01/16 | 27/01/16 | 28/01/16 | 29/01/16 | 30/01/16 | 31/01/16 | Monthly Total |
|---|---|---|---|---|---|---|---|---|---|---|---|---|---|---|---|---|---|---|---|---|---|---|---|---|---|---|---|---|---|---|---|---|---|
| **Brong Ahafo** | Tain | 12 | 0 | 13 | 23 | 0 | 67 | 0 | 9 | 14 | 2 | 1 | 3 | 11 | 0 | 12 | 1 | 8 | 4 | 0 | 12 | 0 | 20 | 0 | 0 | 5 | 2 | 6 | 6 | 20 | 0 | 12 | **263** |
| | Tano North | 0 | 0 | 0 | 0 | 0 | 1 | 0 | 0 | 1 | 0 | 0 | 0 | 0 | 0 | 0 | 0 | 0 | 0 | 0 | 0 | 0 | 2 | 4 | 8 | 0 | 3 | 3 | 3 | 15 | 3 | 14 | **57** |
| | Tano South | 0 | 0 | 0 | 0 | 0 | 9 | 0 | 1 | 0 | 0 | 0 | 0 | 0 | 0 | 0 | 0 | 3 | 0 | 0 | 0 | [illegible] | 2 | [illegible] | 7 | 0 | 2 | 0 | 0 | 1 | 0 | 1 | **26** |
| | Techiman | 2 | 0 | 0 | 6 | 0 | 4 | 0 | 3 | 2 | 0 | 0 | 0 | 0 | 0 | 1 | 1 | 1 | 0 | 0 | 3 | 0 | 8 | 6 | 5 | 0 | 0 | 4 | 5 | 13 | 0 | 8 | **72** |
| | **Brong Ahafo Total** | 200 | 23 | 64 | 229 | 27 | 788 | 3 | 230 | 91 | 44 | 76 | 31 | 77 | 5 | 88 | 17 | 56 | 35 | 1 | 55 | 5 | 182 | 37 | 188 | 9 | 92 | 79 | 75 | 227 | 12 | 164 | **3222** |
| **Central** | Agona | 0 | 0 | 0 | 0 | 0 | 0 | 0 | 1 | 0 | 0 | 0 | 0 | 0 | 0 | 0 | 0 | 0 | 0 | [illegible] | 0 | 0 | 0 | 0 | 1 | 0 | 0 | 0 | 0 | 1 | 0 | 0 | **3** |
| | Ajumako-Enyan-Esiam | 0 | 0 | 0 | 0 | 0 | 1 | 0 | 0 | 0 | 0 | 0 | 0 | 0 | 0 | 0 | 0 | 0 | 0 | 0 | 0 | 0 | 0 | 0 | 0 | 0 | 0 | 0 | 0 | 0 | 0 | 0 | **1** |
| | Asikuma Odoben | 0 | 0 | 0 | 0 | 0 | 1 | 0 | 0 | 0 | 0 | 0 | 0 | 0 | 0 | [illegible] | 0 | 0 | 0 | 0 | 0 | 0 | 0 | 0 | 0 | 0 | 0 | 0 | 0 | 4 | 0 | 3 | **8** |
| | Assin North | 0 | 0 | 0 | 0 | 0 | 0 | 0 | 0 | 0 | 0 | 0 | 0 | 0 | [illegible] | 0 | 0 | 0 | 0 | 0 | 0 | 0 | 0 | 0 | 1 | 0 | 0 | 0 | 0 | 0 | 0 | 3 | **4** |
| | Assin South | 0 | 0 | 0 | 0 | 0 | 0 | 0 | 0 | 0 | 0 | 0 | 0 | [illegible] | 0 | 1 | 0 | 0 | 0 | 0 | 0 | 0 | 2 | 0 | 1 | 0 | 0 | 0 | 0 | 1 | 0 | 1 | **6** |
| | Awutu Efutu Senya | 1 | 0 | 0 | 0 | 0 | 5 | 0 | 3 | 0 | 2 | 0 | 0 | 0 | 0 | 1 | 0 | 0 | 0 | 0 | 0 | 0 | 0 | 0 | 0 | 0 | 0 | 1 | 0 | 4 | 0 | 0 | **17** |
| | Cape Coast | 0 | 0 | 0 | 0 | 0 | 0 | 0 | 1 | 0 | 0 | 0 | 0 | 0 | 0 | 0 | 0 | 0 | 0 | 0 | 0 | 0 | 0 | 0 | 0 | 0 | 0 | 0 | 0 | 0 | 0 | 0 | **1** |
| | Gomoa | 0 | 2 | 0 | 1 | 0 | 7 | 0 | 8 | 0 | 0 | 0 | 0 | 0 | 0 | 0 | 0 | 0 | 0 | 0 | 0 | 0 | 2 | 0 | 1 | 0 | 0 | 0 | 0 | 8 | 0 | 5 | **34** |
| | Komenda-Edina-Eguafo- | 0 | 0 | 0 | 1 | 0 | 1 | 0 | [illegible] | 0 | 0 | 0 | 0 | 0 | 0 | 0 | 0 | 0 | 0 | 0 | 0 | 0 | 0 | 0 | 0 | 0 | 0 | 0 | 0 | 2 | 0 | 0 | **4** |
| | Lower Denkyira | 0 | 0 | 0 | 0 | 0 | [illegible] | 0 | 0 | 0 | 0 | 0 | 0 | 0 | 0 | 1 | 0 | 0 | 0 | 0 | 0 | 0 | 1 | 0 | 1 | 0 | 0 | 0 | 0 | 2 | 0 | 0 | **5** |
| | Mfantsiman | 0 | 0 | 0 | 0 | 0 | 0 | 0 | 2 | 0 | 0 | 0 | 0 | 0 | 0 | 0 | 0 | 0 | 0 | 0 | 0 | 0 | 0 | 0 | 0 | 0 | 0 | 0 | 0 | 1 | 0 | 0 | **3** |
| | Upper Denkyira | 0 | 0 | 0 | [illegible] | [illegible] | 0 | 0 | 0 | 0 | 0 | 0 | 0 | 0 | 0 | 0 | 0 | 1 | 0 | 0 | 0 | 0 | 0 | 0 | 0 | 0 | 0 | 0 | 0 | 0 | 0 | 1 | **2** |
| | **Central Total** | 1 | [illegible] | 0 | 2 | 0 | 15 | 0 | 15 | 0 | 2 | 0 | 0 | 0 | 0 | 3 | 0 | 1 | 0 | 0 | 0 | 0 | 5 | 0 | 5 | 0 | 0 | 1 | 0 | 23 | 0 | 13 | **88** |
| **Eastern** | Afram Plains | 28 | 13 | 15 | 45 | 7 | 89 | 2 | 102 | 8 | 10 | 6 | 2 | 20 | 0 | 17 | 0 | 0 | 2 | 4 | 1 | 0 | 13 | 0 | 22 | 0 | 8 | 10 | 2 | 21 | 0 | 34 | **481** |
| | Akwapim North | 0 | 0 | 0 | 2 | 0 | 13 | 3 | 8 | 2 | 0 | 0 | 0 | 0 | 0 | 0 | 0 | 0 | 0 | 0 | 0 | 0 | 0 | 0 | 1 | 0 | 1 | 0 | 0 | 2 | 0 | 1 | **33** |

EORIC - UENR COPY

*Daily Fire Detection count > 50 are highlighted in orange and Fire detection >=100 are highlighted in red.*

| Jan-2016 | | 01/01/16 | 02/01/16 | 03/01/16 | 04/01/16 | 05/01/16 | 06/01/16 | 07/01/16 | 08/01/16 | 09/01/16 | 10/01/16 | 11/01/16 | 12/01/16 | 13/01/16 | 14/01/16 | 15/01/16 | 16/01/16 | 17/01/16 | 18/01/16 | 19/01/16 | 20/01/16 | 21/01/16 | 22/01/16 | 23/01/16 | 24/01/16 | 5/01/16 | 26/01/16 | 27/01/16 | 28/01/16 | 29/01/16 | 30/01/16 | 31/01/16 | Monthly Total |
|---|---|---|---|---|---|---|---|---|---|---|---|---|---|---|---|---|---|---|---|---|---|---|---|---|---|---|---|---|---|---|---|---|---|
| **Eastern** | Akwapim South | 0 | 0 | 0 | 0 | 0 | 6 | 1 | 5 | 0 | 0 | 0 | 0 | 0 | 0 | 0 | 0 | 0 | 0 | 0 | 0 | 0 | 1 | 0 | 0 | 0 | 0 | 1 | 0 | 2 | 0 | 0 | **16** |
| | Asuogyaman | 1 | 2 | 0 | 0 | 1 | 14 | 2 | 4 | 0 | 3 | 0 | 0 | 1 | 0 | 0 | 0 | 0 | 0 | 0 | 1 | 1 | 0 | 0 | 5 | 0 | 0 | 0 | 0 | 0 | 0 | 0 | **35** |
| | Atiwa | 0 | 0 | 0 | 0 | 0 | 0 | 0 | 1 | 0 | 0 | 0 | 0 | 0 | 0 | 0 | 0 | 0 | 0 | 0 | 0 | | 1 | | 0 | 0 | 0 | 0 | 0 | 1 | 0 | 2 | **5** |
| | Birim North | 0 | 0 | 0 | 0 | 0 | 1 | 0 | 0 | 0 | 0 | 0 | 0 | 0 | 0 | 2 | 0 | 0 | 0 | 1 | 0 | 0 | 0 | 0 | 0 | 0 | 0 | 0 | 0 | 0 | 0 | 0 | **4** |
| | Birim South | 0 | 0 | 0 | 0 | 0 | 1 | 0 | 0 | 0 | 0 | 0 | 0 | 1 | 0 | 1 | 0 | 0 | 0 | | 0 | 0 | 0 | 0 | 0 | 0 | 0 | 0 | 0 | 6 | 0 | 1 | **10** |
| | East Akim | 0 | 0 | 0 | 0 | 0 | 3 | 0 | 4 | 0 | 0 | 0 | 0 | 0 | 0 | 0 | 0 | 0 | 0 | | 0 | 0 | 1 | 0 | 0 | 0 | 0 | 0 | 0 | 1 | 0 | 0 | **9** |
| | Fanteakwa | 2 | 3 | 8 | 9 | 3 | 31 | 7 | 43 | 3 | 3 | 3 | 0 | 0 | 0 | 1 | 0 | 0 | 0 | 0 | 0 | 0 | 4 | 0 | 6 | 0 | 3 | 4 | 6 | 13 | 0 | 6 | **158** |
| | Kwabibirem | 0 | 0 | 0 | 1 | 0 | 1 | 0 | 1 | 0 | 0 | 0 | 0 | 0 | 0 | | 0 | 0 | 0 | 0 | 0 | 0 | 0 | 0 | 1 | 0 | 0 | 0 | 0 | 3 | 0 | 1 | **8** |
| | Kwahu South | 9 | 4 | 6 | 17 | 0 | 56 | 7 | 43 | 0 | 1 | 5 | 0 | 0 | | 1 | 0 | 1 | 0 | 0 | 0 | 0 | 2 | 0 | 8 | 0 | 8 | 2 | 1 | 13 | 1 | 21 | **206** |
| | Kwahu West | 0 | 0 | 0 | 0 | 0 | 1 | 0 | 2 | 2 | 0 | 0 | 0 | | 0 | 0 | 0 | 0 | 0 | 0 | 0 | 0 | 0 | 0 | 0 | 0 | 0 | 1 | 0 | 0 | 0 | 2 | **8** |
| | Manya Krobo | 4 | 0 | 0 | 2 | 0 | 44 | 5 | 43 | 2 | 0 | 0 | 0 | 3 | 0 | 0 | 0 | 0 | 2 | 0 | 0 | 0 | 5 | 0 | 4 | 0 | 2 | 4 | 2 | 6 | 3 | 12 | **143** |
| | New Juaben | 0 | 0 | 0 | 0 | 0 | 2 | 0 | 6 | 0 | 0 | 0 | 0 | 0 | 0 | 0 | 0 | 0 | 0 | 0 | 0 | 0 | 2 | 0 | 0 | 0 | 0 | 0 | 0 | 4 | 0 | 1 | **15** |
| | Suhum Kraboa | 0 | 0 | 0 | 1 | 0 | 10 | 0 | 5 | 0 | 0 | 0 | 0 | 2 | 0 | 0 | 0 | 0 | 0 | 0 | 1 | 0 | 0 | 0 | 0 | 0 | 0 | 1 | 0 | 2 | 0 | 6 | **28** |
| | West Akim | 0 | 0 | 0 | 0 | 0 | 0 | 0 | 1 | 0 | 0 | 0 | 0 | 0 | 0 | 0 | 0 | 0 | 0 | 0 | 0 | 0 | 2 | 0 | 0 | 0 | 0 | 0 | 0 | 1 | 0 | 2 | **6** |
| | Yilo Krobo | 1 | 0 | 7 | 4 | 0 | 4 | 11 | 40 | 1 | 0 | 0 | 2 | 0 | 0 | 0 | 0 | 2 | 0 | 0 | 0 | 0 | 0 | 0 | 1 | 0 | 0 | 0 | 0 | 5 | 0 | 7 | **105** |
| | **Eastern Total** | 45 | 22 | 3 | 81 | 11 | 296 | 38 | 308 | 18 | 17 | 14 | 4 | 27 | 0 | 22 | 0 | 3 | 4 | 5 | 3 | 1 | 31 | 0 | 48 | 0 | 22 | 23 | 11 | 80 | 4 | 96 | **1270** |
| **Greater Accra** | Dangbe East | 0 | 0 | 0 | 2 | | 5 | 1 | 15 | 1 | 0 | 0 | 0 | 0 | 0 | 2 | 0 | 0 | 0 | 0 | 0 | 0 | 0 | 0 | 0 | 0 | 0 | 1 | 0 | 6 | 0 | 1 | **34** |
| | Dangbe West | 0 | 0 | 0 | 1 | 0 | 0 | 0 | 1 | 0 | 0 | 0 | 0 | 0 | 0 | 0 | 0 | 0 | 0 | 0 | 0 | 0 | 0 | 0 | 0 | 0 | 0 | 0 | 0 | 1 | 0 | 0 | **3** |
| | Ga East | 0 | 0 | 0 | 0 | 0 | 1 | 0 | 0 | 0 | 0 | 0 | 0 | 0 | 0 | 0 | 0 | 0 | 0 | 0 | 0 | 0 | 0 | 0 | 0 | 0 | 0 | 0 | 0 | 1 | 0 | 0 | **2** |

C - UENR

*Daily Fire Detection count > 50 are highlighted in orange and Fire detection >=100 are highlighted in red.*

| Jan-2016 | | 01/01/16 | 02/01/16 | 03/01/16 | 04/01/16 | 05/01/16 | 06/01/16 | 07/01/16 | 08/01/16 | 09/01/16 | 10/01/16 | 11/01/16 | 12/01/16 | 13/01/16 | 14/01/16 | 15/01/16 | 16/01/16 | 17/01/16 | 18/01/16 | 19/01/16 | 20/01/16 | 21/01/16 | 22/01/16 | 23/01/16 | 24/01/16 | 5/01/16 | 26/01/16 | 27/01/16 | 28/01/16 | 29/01/16 | 30/01/16 | 31/01/16 | Monthly Total |
|---|---|---|---|---|---|---|---|---|---|---|---|---|---|---|---|---|---|---|---|---|---|---|---|---|---|---|---|---|---|---|---|---|---|
| **Greater Accra** | Ga West | 0 | 0 | 0 | 0 | 0 | 7 | 0 | 1 | 0 | 0 | 0 | 0 | 0 | 0 | 0 | 0 | 0 | 0 | 0 | 0 | 0 | 0 | 0 | 0 | 0 | 0 | 0 | 0 | 1 | 0 | 0 | **9** |
| | **Greater Accra Total** | 0 | 0 | 0 | 3 | 0 | 13 | 1 | 17 | 1 | 0 | 0 | 0 | 0 | 0 | 2 | 0 | 0 | 0 | 0 | 0 | 0 | 0 | 0 | 0 | 0 | 0 | 1 | 0 | 9 | 0 | 1 | **48** |
| **Northern** | Bole | 10 | 2 | 3 | 11 | 0 | 33 | 0 | 12 | 6 | 4 | 13 | 3 | 20 | 0 | 4 | 0 | 8 | 1 | 0 | 6 | | 9 | | 0 | 0 | 0 | 1 | 0 | 4 | 0 | 5 | **155** |
| | Bunkpurugu Yunyoo | 5 | 3 | 2 | 0 | 0 | 6 | 0 | 8 | 0 | 2 | 3 | 6 | 7 | 0 | 2 | 0 | 8 | 0 | 1 | 5 | 0 | 2 | 0 | 1 | 0 | 0 | 0 | 0 | 0 | 0 | 5 | **66** |
| | Central Gonja | 44 | 5 | 3 | 31 | 0 | 93 | 1 | 18 | 11 | 21 | 17 | 1 | 20 | 0 | 24 | 2 | 22 | 11 | 1 | 16 | 0 | 25 | 0 | 1 | 4 | 6 | 11 | 2 | 11 | 0 | 27 | **428** |
| | East Gonja | 70 | 7 | 3 | 38 | 13 | 175 | 6 | 28 | 10 | 13 | 44 | 16 | 58 | 9 | 52 | 3 | 48 | 13 | 14 | 23 | 6 | 56 | 2 | 27 | 1 | 30 | 25 | 2 | 22 | 0 | 38 | **852** |
| | East Mamprusi | 2 | 0 | 0 | 0 | 0 | 7 | 0 | 7 | 1 | 5 | 6 | 10 | 6 | 0 | 7 | 0 | 3 | 0 | 2 | 2 | 0 | 1 | 0 | 1 | 0 | 1 | 2 | 0 | 3 | 0 | 0 | **66** |
| | Gushiegu | 23 | 10 | 9 | 2 | 7 | 26 | 0 | 61 | 15 | 9 | 35 | 24 | 22 | 8 | 18 | 4 | 25 | 5 | 18 | 18 | 4 | 14 | 0 | 0 | 2 | 9 | 14 | 1 | 10 | 9 | 7 | **409** |
| | Karaga | 11 | 8 | 0 | 5 | 3 | 23 | 3 | 12 | 8 | 13 | 32 | 18 | 30 | 3 | 10 | 0 | 21 | 6 | 9 | 24 | 3 | 8 | 1 | 7 | 5 | 11 | 1 | 3 | 2 | 5 | 12 | **297** |
| | Nanumba North | 12 | 0 | 0 | 27 | 0 | 56 | 0 | 15 | 3 | 11 | 14 | 12 | 5 | 7 | 8 | 5 | 21 | 5 | 22 | 11 | 5 | 7 | 0 | 2 | 1 | 13 | 14 | 0 | 0 | 0 | 12 | **308** |
| | Nanumba South | 9 | 1 | 0 | 7 | 0 | 7 | 0 | 0 | 3 | 8 | 21 | 3 | 14 | 4 | 8 | 0 | 12 | 2 | 5 | 7 | 2 | 2 | 0 | 3 | 1 | 9 | 9 | 2 | 1 | 1 | 6 | **147** |
| | Saboba Chereponi | 7 | 4 | 4 | 1 | 0 | 6 | 6 | 1 | 11 | 18 | 4 | 10 | 22 | 3 | 9 | 0 | 27 | 11 | 9 | 5 | 6 | 10 | 0 | 1 | 0 | 4 | 19 | 3 | 3 | 3 | 23 | **246** |
| | Savelugu Nanton | 5 | 8 | 5 | 0 | 1 | 12 | 2 | 1 | 2 | 9 | 11 | 5 | 11 | 1 | 8 | 0 | 20 | 6 | 6 | 9 | 0 | 5 | 0 | 0 | 0 | 2 | 2 | 0 | 1 | 2 | 0 | **145** |
| | Sawa-Tuna-Kalba | 8 | 5 | 0 | 1 | 0 | 13 | 0 | 2 | 6 | 3 | 8 | 11 | 13 | 0 | 11 | 1 | 17 | 12 | 1 | 6 | 1 | 10 | 1 | 0 | 1 | 1 | 8 | 0 | 3 | 0 | 4 | **147** |
| | Tamale | 1 | 0 | 2 | 1 | 0 | 12 | 0 | 1 | 2 | 2 | 0 | 0 | 2 | 0 | 6 | 0 | 0 | 3 | 3 | 1 | 0 | 1 | 0 | 0 | 0 | 0 | 0 | 0 | 0 | 0 | 1 | **38** |
| | Tolon-Kumbungu | 10 | 6 | 0 | 0 | 2 | 35 | 0 | 9 | 12 | 19 | 15 | 3 | 31 | 0 | 7 | 2 | 14 | 8 | 3 | 4 | 2 | 9 | 0 | 0 | 2 | 0 | 0 | 0 | 1 | 0 | 5 | **199** |
| | West Gonja | 59 | 27 | 2 | 0 | | 112 | 6 | 75 | 33 | 42 | 73 | 17 | 79 | 0 | 35 | 4 | 20 | 17 | 18 | 26 | 3 | 36 | 3 | 12 | 3 | 2 | 15 | 0 | 24 | 0 | 15 | **778** |
| | West Mamprusi | 10 | | 0 | 0 | 1 | 36 | 0 | 14 | 1 | 8 | 9 | 10 | 11 | 1 | 16 | 0 | 22 | 1 | 3 | 10 | 0 | 13 | 2 | 0 | 0 | 0 | 1 | 0 | 7 | 0 | 2 | **182** |
| | Yendi | 18 | 0 | 5 | 12 | 3 | 42 | 2 | 12 | 4 | 14 | 23 | 12 | 38 | 5 | 31 | 13 | 45 | 10 | 26 | 18 | 12 | 36 | 2 | 3 | 10 | 11 | 13 | 8 | 8 | 3 | 40 | **479** |

C-UENR

*Daily Fire Detection count > 50 are highlighted in orange and Fire detection >=100 are highlighted in red.*

| Jan-2016 | | 01/01/16 | 02/01/16 | 03/01/16 | 04/01/16 | 05/01/16 | 06/01/16 | 07/01/16 | 08/01/16 | 09/01/16 | 10/01/16 | 11/01/16 | 12/01/16 | 13/01/16 | 14/01/16 | 15/01/16 | 16/01/16 | 17/01/16 | 18/01/16 | 19/01/16 | 20/01/16 | 21/01/16 | 22/01/16 | 23/01/16 | 24/01/16 | 5/01/16 | 26/01/16 | 27/01/16 | 28/01/16 | 29/01/16 | 30/01/16 | 31/01/16 | Monthly Total |
|---|---|---|---|---|---|---|---|---|---|---|---|---|---|---|---|---|---|---|---|---|---|---|---|---|---|---|---|---|---|---|---|---|---|
| **Northern** | Zabzugu Tatale | 8 | 2 | 0 | 3 | 2 | 45 | 4 | 13 | 9 | 20 | 20 | 10 | 27 | 6 | 14 | 4 | 26 | 5 | 1 | 5 | 7 | 12 | 0 | 4 | 0 | 8 | 4 | 0 | 2 | 7 | 6 | **274** |
| | **Northern Total** | 312 | 92 | 38 | 159 | 32 | 739 | 30 | 316 | 137 | 221 | 348 | 171 | 436 | 47 | 270 | 38 | 359 | 116 | 142 | 196 | 51 | 256 | 11 | 62 | 30 | 107 | 139 | 21 | 102 | 30 | 208 | **5216** |
| **Upper East** | Bawku West | 0 | 0 | 0 | 0 | 0 | 3 | 0 | 1 | 0 | 0 | 0 | 2 | 1 | 0 | 5 | 0 | 2 | 0 | 0 | 2 | | 1 | | 0 | 1 | 0 | 0 | 0 | 0 | 0 | 0 | **18** |
| | Bolgatanga | 0 | 0 | 0 | 0 | 0 | 0 | 0 | 0 | 1 | 0 | 0 | 0 | 0 | 0 | 0 | 0 | 0 | 0 | 0 | 1 | 0 | 0 | 0 | 0 | 0 | 0 | 0 | 0 | 1 | 0 | 1 | **4** |
| | Bongo | 2 | 0 | 0 | 0 | 0 | 0 | 0 | 0 | 0 | 0 | 0 | 0 | 0 | 0 | 0 | 0 | 0 | 0 | | 0 | 0 | 0 | 0 | 0 | 0 | 0 | 2 | 0 | 0 | 0 | 0 | **4** |
| | Builsa | 0 | 0 | 0 | 0 | 0 | 3 | 0 | 1 | 2 | 0 | 0 | 0 | 9 | 0 | 5 | 0 | 1 | 0 | | 1 | 3 | 10 | 0 | 1 | 0 | 0 | 3 | 3 | 5 | 0 | 0 | **47** |
| | Garu Tempane | 0 | 0 | 0 | 0 | 0 | 5 | 0 | 1 | 0 | 0 | 0 | 0 | 0 | 0 | 0 | 0 | 0 | 0 | 0 | 1 | 0 | 3 | 0 | 2 | 0 | 0 | 0 | 0 | 0 | 0 | 0 | **12** |
| | Kassena Nankana | 2 | 1 | 0 | 0 | 0 | 9 | 0 | 1 | 0 | 1 | 0 | 0 | 7 | 0 | | 0 | 5 | 3 | 0 | 0 | 0 | 0 | 0 | 0 | 3 | 0 | 3 | 0 | 2 | 0 | 0 | **39** |
| | Talensi Nabdam | 1 | 0 | 0 | 0 | 0 | 1 | 0 | 0 | 0 | 0 | 0 | 0 | 5 | | 0 | 0 | 0 | 0 | 0 | 0 | 0 | 2 | 0 | 0 | 0 | 0 | 0 | 0 | 0 | 0 | 1 | **10** |
| | **Upper East Total** | 5 | 1 | 0 | 0 | 0 | 21 | 0 | 4 | 3 | 1 | 0 | 2 | 22 | 0 | 12 | 0 | 8 | 3 | 0 | 5 | 3 | 16 | 0 | 3 | 4 | 0 | 8 | 3 | 8 | 0 | 2 | **134** |
| **Upper West** | Jirapa Lambussie | 0 | 0 | 0 | 0 | 0 | 1 | 0 | 0 | 0 | 0 | 0 | 2 | 0 | 0 | 1 | 0 | 0 | 0 | 0 | 3 | 0 | 0 | 0 | 0 | 0 | 0 | 0 | 0 | 5 | 0 | 0 | **12** |
| | Lawra | 0 | 0 | 0 | 2 | 0 | 2 | 0 | 0 | 0 | 1 | 4 | 0 | 0 | 0 | 1 | 0 | 0 | 1 | 0 | 1 | 0 | 0 | 0 | 0 | 0 | 0 | 1 | 0 | 1 | 0 | 0 | **14** |
| | Nadowli | 5 | 4 | 0 | 1 | 0 | 9 | 0 | 1 | 1 | 5 | 4 | 0 | 2 | 0 | 0 | 0 | 0 | 0 | 0 | 1 | 0 | 7 | 0 | 0 | 0 | 1 | 0 | 0 | 3 | 3 | 1 | **48** |
| | Sissala East | 4 | 9 | 0 | 20 | 4 | 42 | 0 | | 3 | 4 | 12 | 8 | 30 | 0 | 14 | 4 | 14 | 0 | 6 | 7 | 0 | 23 | 1 | 1 | 2 | 2 | 1 | 0 | 2 | 0 | 2 | **219** |
| | Sissala West | 9 | 10 | 0 | 1 | 0 | 3 | 0 | 0 | 3 | 10 | 10 | 0 | 8 | 0 | 7 | 2 | 3 | 5 | 0 | 8 | 0 | 4 | 0 | 0 | 3 | 0 | 2 | 0 | 1 | 0 | 3 | **112** |
| | Wa | 2 | 6 | 0 | 0 | 0 | 13 | 0 | 1 | 0 | 3 | 6 | 0 | 5 | 0 | 2 | 0 | 4 | 2 | 0 | 3 | 0 | 2 | 0 | 2 | 0 | 0 | 0 | 0 | 1 | 0 | 3 | **55** |
| | Wa East | 11 | 5 | 1 | | | 39 | 0 | 1 | 11 | 15 | 17 | 2 | 31 | 0 | 13 | 0 | 0 | 2 | 5 | 13 | 0 | 6 | 0 | 2 | 0 | 1 | 5 | 1 | 5 | 0 | 3 | **189** |
| | Wa West | 0 | 0 | 0 | 0 | 0 | 2 | 0 | 0 | 0 | 0 | 1 | 0 | 2 | 0 | 0 | 0 | 0 | 0 | 0 | 0 | 0 | 0 | 0 | 0 | 0 | 0 | 1 | 0 | 0 | 0 | 0 | **6** |
| | **Upper West Total** | 31 | 34 | 1 | 24 | 4 | 131 | 0 | 7 | 18 | 38 | 54 | 12 | 78 | 0 | 38 | 6 | 21 | 10 | 11 | 36 | 0 | 42 | 1 | 5 | 5 | 4 | 10 | 1 | 18 | 3 | 12 | **655** |

C - UENR

*Daily Fire Detection count > 50 are highlighted in orange and Fire detection >=100 are highlighted in red.*

| Jan-2016 | | 01/01/16 | 02/01/16 | 03/01/16 | 04/01/16 | 05/01/16 | 06/01/16 | 07/01/16 | 08/01/16 | 09/01/16 | 10/01/16 | 11/01/16 | 12/01/16 | 13/01/16 | 14/01/16 | 15/01/16 | 16/01/16 | 17/01/16 | 18/01/16 | 19/01/16 | 20/01/16 | 21/01/16 | 22/01/16 | 23/01/16 | 24/01/16 | 5/01/16 | 26/01/16 | 27/01/16 | 28/01/16 | 29/01/16 | 30/01/16 | 31/01/16 | Monthly Total |
|---|---|---|---|---|---|---|---|---|---|---|---|---|---|---|---|---|---|---|---|---|---|---|---|---|---|---|---|---|---|---|---|---|---|
| **Volta** | Adaklu Anyigbe | 26 | 18 | 11 | 36 | 0 | 48 | 3 | 17 | 0 | 5 | 0 | 3 | 7 | 0 | 1 | 0 | 2 | 0 | 0 | 0 | 0 | 0 | 0 | 6 | 0 | 3 | 1 | 10 | 8 | 1 | 4 | **210** |
| | Akatsi | 0 | 4 | 1 | 3 | 0 | 11 | 0 | 6 | 0 | 0 | 0 | 0 | 1 | 0 | 3 | 0 | 1 | 0 | 0 | 0 | 0 | 1 | 0 | 3 | 0 | 2 | 5 | 0 | 3 | 0 | 2 | **46** |
| | Ho | 8 | 5 | 2 | 5 | 0 | 35 | 7 | 22 | 0 | 2 | 4 | 1 | 4 | 1 | 0 | 0 | 4 | 3 | 0 | 0 | | 4 | | 12 | 0 | 3 | 4 | 4 | 9 | 0 | 8 | **147** |
| | Hohoe | 1 | 1 | 1 | 1 | 0 | 2 | 0 | 5 | 0 | 1 | 0 | 0 | 7 | 0 | 0 | 0 | 1 | 0 | 0 | 0 | 0 | 31 | 0 | 28 | 1 | 3 | 17 | 4 | 15 | 4 | 8 | **131** |
| | Jasikan | 2 | 0 | 0 | 5 | 0 | 5 | 0 | 2 | 0 | 2 | 0 | 0 | 6 | 0 | 2 | 0 | 1 | 0 | 1 | 1 | 2 | 17 | 2 | 36 | 5 | 7 | 9 | 3 | 20 | 5 | 16 | **149** |
| | Kadjebi | 2 | 0 | 0 | 0 | 0 | 1 | 0 | 6 | 0 | 5 | 0 | 1 | 5 | 2 | 0 | 0 | 0 | 0 | | 0 | 1 | 10 | 1 | 53 | 0 | 3 | 0 | 2 | 0 | 0 | 7 | **99** |
| | Keta | 0 | 0 | 0 | 0 | 0 | 1 | 0 | 3 | 0 | 0 | 0 | 0 | 0 | 0 | 0 | 0 | 0 | 0 | 0 | 0 | 0 | 0 | 0 | 0 | 0 | 0 | 0 | 1 | 1 | 0 | 0 | **6** |
| | Ketu | 0 | 1 | 0 | 1 | 0 | 2 | 0 | 2 | 0 | 0 | 0 | 0 | 0 | 0 | | 0 | 0 | 0 | 0 | 0 | 0 | 0 | 0 | 0 | 0 | 0 | 0 | 0 | 0 | 0 | 0 | **6** |
| | Kpandu | 2 | 0 | 0 | 0 | 0 | 1 | 0 | 7 | 1 | 2 | 0 | 1 | 0 | | 1 | 0 | 2 | 0 | 0 | 0 | 0 | 5 | 0 | 18 | 0 | 0 | 6 | 0 | 7 | 3 | 13 | **69** |
| | Krachi | 18 | 0 | 2 | 11 | 2 | 37 | 0 | 8 | 1 | 4 | 3 | 5 | 0 | 4 | 11 | 0 | 5 | 0 | 4 | 3 | 3 | 4 | 0 | 2 | 0 | 1 | 2 | 1 | 5 | 0 | 1 | **157** |
| | Krachi East | 31 | 5 | 1 | 5 | 0 | 41 | 3 | 17 | 3 | 1 | 5 | 1 | 29 | 7 | 19 | 0 | 1 | 0 | 0 | 4 | 6 | 32 | 1 | 47 | 1 | 2 | 10 | 3 | 6 | 0 | 6 | **287** |
| | Nkwanta | 14 | 1 | 0 | 20 | 4 | 66 | 0 | 4 | 19 | 15 | 26 | 24 | 62 | 1 | 40 | 3 | 24 | 14 | 9 | 9 | 4 | 73 | 4 | 179 | 5 | 21 | 11 | 16 | 16 | 6 | 21 | **751** |
| | North Tongu | 2 | 3 | 2 | 1 | 0 | 13 | 0 | 1 | 0 | 0 | 1 | 0 | 0 | 0 | 1 | 0 | 1 | 1 | 0 | 0 | 0 | 0 | 0 | 2 | 0 | 0 | 4 | 3 | 4 | 0 | 10 | **61** |
| | South Dayi | 0 | 3 | 8 | 1 | 2 | 12 | 0 | 5 | 0 | 1 | 2 | 0 | 1 | 0 | 0 | 0 | 1 | 0 | 0 | 0 | 0 | 3 | 0 | 0 | 0 | 2 | 4 | 0 | 0 | 0 | 0 | **45** |
| | South Tongu | 0 | 0 | 0 | 0 | 0 | 1 | 0 | 2 | 0 | 0 | 0 | 0 | 0 | 0 | 2 | 0 | 0 | 0 | 0 | 0 | 0 | 0 | 0 | 0 | 0 | 0 | 0 | 0 | 1 | 0 | 0 | **6** |
| | **Volta Total** | 106 | 41 | 2 | 89 | 8 | 276 | 13 | 159 | 24 | 38 | 41 | 36 | 142 | 15 | 80 | 3 | 43 | 18 | 14 | 17 | 16 | 180 | 8 | 386 | 12 | 47 | 73 | 47 | 95 | 19 | 96 | **2170** |
| **Western** | Aowin-Suaman | 0 | 0 | 0 | | | 0 | 0 | 1 | 0 | 0 | 0 | 0 | 0 | 0 | 0 | 0 | 0 | 0 | 0 | 0 | 0 | 0 | 0 | 0 | 0 | 0 | 0 | 0 | 2 | 0 | 0 | **3** |
| | Bia | 0 | 0 | 0 | 0 | 0 | 1 | 0 | 0 | 0 | 0 | 0 | 0 | 0 | 0 | 0 | 0 | 0 | 0 | 0 | 0 | 0 | 0 | 0 | 0 | 0 | 0 | 0 | 0 | 2 | 0 | 0 | **3** |
| | Bibiani Anhwiaso | 0 | 0 | 0 | 0 | 0 | 5 | 2 | 10 | 1 | 0 | 0 | 0 | 0 | 0 | 1 | 0 | 0 | 0 | 0 | 0 | 0 | 0 | 0 | 0 | 0 | 3 | 1 | 6 | 14 | 1 | 1 | **45** |
| | Jomoro | 0 | 0 | 0 | 0 | 0 | 1 | 0 | 0 | 0 | 0 | 0 | 0 | 0 | 0 | 0 | 0 | 0 | 0 | 0 | 0 | 0 | 0 | 0 | 0 | 0 | 0 | 0 | 0 | 2 | 0 | 0 | **3** |

C-UENR

*Daily Fire Detection count > 50 are highlighted in orange and Fire detection >=100 are highlighted in red.*

| Jan-2016 | | 01/01/16 | 02/01/16 | 03/01/16 | 04/01/16 | 05/01/16 | 06/01/16 | 07/01/16 | 08/01/16 | 09/01/16 | 10/01/16 | 11/01/16 | 12/01/16 | 13/01/16 | 14/01/16 | 15/01/16 | 16/01/16 | 17/01/16 | 18/01/16 | 19/01/16 | 20/01/16 | 21/01/16 | 22/01/16 | 23/01/16 | 24/01/16 | 5/01/16 | 26/01/16 | 27/01/16 | 28/01/16 | 29/01/16 | 30/01/16 | 31/01/16 | Monthly Total |
|---|---|---|---|---|---|---|---|---|---|---|---|---|---|---|---|---|---|---|---|---|---|---|---|---|---|---|---|---|---|---|---|---|---|
| **Western** | Juabeso | 0 | 0 | 0 | 0 | 0 | 0 | 0 | 0 | 0 | 0 | 0 | 0 | 0 | 0 | 0 | 0 | 0 | 0 | 0 | 0 | 0 | 0 | 0 | 0 | 1 | 1 | 0 | 4 | 14 | 0 | 12 | **32** |
| | Mpohor Wassa East | 0 | 0 | 0 | 1 | 0 | 3 | 0 | 1 | 2 | 0 | 0 | 0 | 0 | 0 | 1 | 0 | 0 | 0 | 0 | 0 | 0 | 1 | 0 | 0 | 0 | 0 | 2 | 0 | 6 | 0 | 4 | **21** |
| | Nzema East | 0 | 0 | 0 | 0 | 0 | 1 | 0 | 0 | 0 | 0 | 0 | 0 | 0 | 0 | 0 | 0 | 0 | 0 | 0 | 0 | | 0 | | 0 | 0 | 0 | 1 | 0 | 2 | 0 | 0 | **4** |
| | Sefwi Wiawso | 0 | 0 | 0 | 0 | 0 | 0 | 0 | 0 | 0 | 0 | 0 | 0 | 0 | 0 | 0 | 0 | 0 | 0 | 0 | 0 | 0 | 0 | 0 | 1 | 0 | 0 | 0 | 2 | 2 | 0 | 0 | **5** |
| | Shama Ahanta East | 0 | 0 | 0 | 0 | 0 | 2 | 0 | 0 | 0 | 0 | 0 | 0 | 2 | 0 | 0 | 0 | 0 | 0 | | 0 | 0 | 0 | 0 | 0 | 0 | 0 | 0 | 0 | 5 | 0 | 0 | **9** |
| | Wasa Amenfi East | 0 | 0 | 0 | 0 | 0 | 2 | 0 | 0 | 0 | 0 | 0 | 0 | 0 | 0 | 0 | 0 | 0 | 0 | | 0 | 0 | 4 | 0 | 1 | 0 | 0 | 0 | 0 | 0 | 0 | 0 | **7** |
| | Wasa Amenfi West | 0 | 0 | 0 | 0 | 0 | 0 | 0 | 0 | 0 | 0 | 0 | 0 | 0 | 0 | 1 | 0 | 0 | 0 | 0 | 0 | 0 | 2 | 0 | 1 | 0 | 0 | 0 | 0 | 5 | 0 | 1 | **10** |
| | Wassa West | 0 | 0 | 0 | 2 | 0 | 2 | 0 | 2 | 1 | 0 | 0 | 0 | 1 | 0 | | 0 | 1 | 0 | 0 | 0 | 0 | 1 | 0 | 1 | 0 | 0 | 0 | 0 | 7 | 0 | 7 | **26** |
| | **Western Total** | 0 | 0 | 0 | 3 | 0 | 17 | 2 | 14 | 4 | 0 | 0 | 0 | 3 | 0 | 4 | 0 | 1 | 0 | 0 | 0 | 0 | 8 | 0 | 4 | 1 | 4 | 4 | 12 | 61 | 1 | 25 | **168** |
| **Provincial Total** | | **815** | **232** | **205** | **795** | **107** | **3001** | **143** | **1487** | **345** | **391** | **563** | **270** | **822** | **83** | **583** | **85** | **565** | **208** | **192** | **328** | **76** | **805** | **65** | **888** | **62** | **354** | **399** | **220** | **854** | **99** | **757** | **15799** |

C - IENR

*Daily Fire Detection count > 50 are highlighted in orange and Fire detection >=100 are highlighted in red.*

| Feb-2016 | | 01/02/16 | 02/02/16 | 03/02/16 | 04/02/16 | 05/02/16 | 06/02/16 | 07/02/16 | 08/02/16 | 09/02/16 | 10/02/16 | 11/02/16 | 12/02/16 | 13/02/16 | 14/02/16 | 15/02/16 | 16/02/16 | 17/02/16 | 19/02/16 | 20/02/16 | 21/02/16 | 22/02/16 | 23/02/16 | 25/02/16 | 26/02/16 | 2 /02/16 | 28/02/16 | 29/02/16 | Monthly Total |
|---|---|---|---|---|---|---|---|---|---|---|---|---|---|---|---|---|---|---|---|---|---|---|---|---|---|---|---|---|---|
| **Ashanti** | Adansi North | 0 | 1 | 0 | 0 | 1 | 0 | 1 | 0 | 1 | 0 | 4 | 0 | 0 | 0 | 0 | 0 | 0 | 0 | 0 | 0 | 0 | 0 | 0 | 0 | 0 | 0 | 0 | **8** |
| | Adansi South | 0 | 1 | 0 | 0 | 1 | 0 | 0 | 0 | 0 | 0 | 2 | 1 | 0 | 0 | 0 | 0 | 0 | 0 | 1 | 0 | 0 | 2 | 1 | 0 | 0 | 1 | 0 | **10** |
| | Afigya Sekyere | 1 | 7 | 1 | 0 | 1 | 2 | 1 | 0 | 2 | 2 | 0 | 2 | 0 | 0 | 0 | 1 | 0 | 0 | 0 | 0 | 0 | 0 | 0 | 0 | 0 | 1 | 0 | **21** |
| | Ahafo Ano North | 0 | 3 | 4 | 0 | 2 | 3 | 7 | 0 | 0 | 14 | 3 | 2 | 0 | 0 | 0 | 0 | 0 | 0 | 1 | 3 | 0 | 0 | 0 | 0 | 0 | 2 | 0 | **44** |
| | Ahafo Ano South | 15 | 54 | 54 | 32 | 58 | 20 | 37 | 6 | 8 | 47 | 21 | 12 | 0 | 0 | 0 | 0 | 1 | | 0 | 1 | 0 | 1 | 0 | 0 | 0 | 0 | 0 | **367** |
| | Amansie Central | 0 | 0 | 0 | 0 | 2 | 0 | 0 | 0 | 0 | 5 | 0 | 0 | 0 | 0 | 0 | 0 | 0 | 0 | 0 | 0 | 0 | 2 | 1 | 0 | 0 | 0 | 0 | **10** |
| | Amansie East | 0 | 20 | 6 | 0 | 3 | 0 | 0 | 0 | 2 | 2 | 6 | 1 | 0 | 0 | 0 | 0 | 0 | 0 | 1 | 0 | 0 | 0 | 0 | 0 | 0 | 0 | 0 | **41** |
| | Amansie West | 0 | 4 | 4 | 1 | 9 | 3 | 6 | 0 | 1 | 4 | 4 | 1 | 1 | 0 | 0 | 0 | 0 | 0 | 0 | 0 | 0 | 1 | 0 | 0 | 0 | 0 | 0 | **39** |
| | Asante Akim North | 1 | 9 | 1 | 0 | 0 | 1 | 7 | 0 | 4 | 4 | 0 | 9 | 0 | 1 | 0 | 0 | 0 | 0 | 0 | 0 | 0 | 0 | 0 | 0 | 4 | 1 | 0 | **42** |
| | Asante Akim South | 0 | 7 | 2 | 0 | 2 | 0 | 0 | 0 | 0 | 7 | 2 | 0 | 0 | | 0 | 0 | 0 | 0 | 3 | 0 | 0 | 0 | 0 | 0 | 0 | 0 | 0 | **23** |
| | Atwima | 0 | 3 | 5 | 6 | 13 | 0 | 6 | 0 | 8 | 14 | 4 | 5 | 0 | 1 | 0 | 0 | 0 | 0 | 0 | 1 | 0 | 1 | 1 | 0 | 0 | 0 | 0 | **68** |
| | Atwima Mponua | 0 | 10 | 20 | 0 | 14 | 0 | 27 | | 8 | 21 | 25 | 20 | 2 | 1 | 0 | 0 | 0 | 0 | 0 | 2 | 0 | 0 | 0 | 0 | 0 | 1 | 0 | **153** |
| | Bosomtwe-Kwanwoma | 1 | 0 | 1 | 1 | 5 | 0 | | 0 | 2 | 1 | 0 | 0 | 0 | 1 | 0 | 0 | 0 | 0 | 0 | 0 | 0 | 0 | 0 | 0 | 0 | 0 | 0 | **13** |
| | Ejisu-Juabeng | 0 | 4 | 1 | 0 | 2 | 0 | 3 | 0 | 1 | 4 | 1 | 1 | 0 | 0 | 0 | 0 | 0 | 0 | 0 | 0 | 0 | 0 | 0 | 0 | 0 | 1 | 0 | **18** |
| | Ejura Sekyedumas | 4 | 34 | 24 | 4 | 22 | 1 | 16 | 0 | 11 | 5 | 12 | 4 | 0 | 15 | 0 | 3 | 0 | 3 | 2 | 0 | 0 | 2 | 0 | 0 | 1 | 1 | 0 | **164** |
| | Kumasi | 0 | 0 | 0 | 0 | 1 | 0 | 0 | 0 | 0 | 0 | 0 | 0 | 0 | 0 | 0 | 0 | 0 | 0 | 0 | 0 | 0 | 0 | 0 | 0 | 0 | 0 | 0 | **1** |
| | Kwabre | 1 | 5 | 1 | 0 | 5 | 0 | 2 | 0 | 1 | 1 | 5 | 0 | 0 | 1 | 0 | 0 | 0 | 0 | 0 | 0 | 0 | 0 | 0 | 0 | 0 | 0 | 0 | **22** |
| | Obuasi Municipal | 0 | | 0 | 0 | 0 | 0 | 0 | 0 | 0 | 0 | 0 | 0 | 0 | 2 | 0 | 0 | 0 | 0 | 0 | 0 | 0 | 0 | 0 | 0 | 0 | 0 | 0 | **2** |
| | Offinso | 4 | 36 | 27 | 12 | 29 | 15 | 51 | 5 | 14 | 12 | 7 | 13 | 0 | 2 | 0 | 3 | 0 | 6 | 1 | 0 | 0 | 0 | 1 | 0 | 0 | 8 | 0 | **246** |

C-UENR

*Daily Fire Detection count > 50 are highlighted in orange and Fire detection >=100 are highlighted in red.*

| Feb-2016 | | 01/02/16 | 02/02/16 | 03/02/16 | 04/02/16 | 05/02/16 | 06/02/16 | 07/02/16 | 08/02/16 | 09/02/16 | 10/02/16 | 11/02/16 | 12/02/16 | 13/02/16 | 14/02/16 | 15/02/16 | 16/02/16 | 17/02/16 | 19/02/16 | 20/02/16 | 21/02/16 | 22/02/16 | 23/02/16 | 25/02/16 | 26/02/16 | 7/02/16 | 28/02/16 | 29/02/16 | Monthly Total |
|---|---|---|---|---|---|---|---|---|---|---|---|---|---|---|---|---|---|---|---|---|---|---|---|---|---|---|---|---|---|
| **Ashanti** | Sekyere East | 0 | 18 | 2 | 1 | 14 | 5 | 10 | 0 | 8 | 0 | 10 | 4 | 7 | 3 | 0 | 5 | 0 | 0 | 0 | 1 | 0 | 1 | 0 | 0 | 1 | 1 | 0 | **91** |
| | Sekyere West | 4 | 20 | 13 | 3 | 16 | 4 | 19 | 0 | 11 | 13 | 11 | 8 | 3 | 7 | 0 | 3 | 0 | 3 | 3 | 0 | 0 | 2 | 5 | 0 | 0 | 2 | 0 | **150** |
| | **Ashanti Total** | 31 | 236 | 166 | 60 | 200 | 54 | 194 | 13 | 82 | 156 | 117 | 83 | 13 | 34 | 0 | 15 | 1 | 12 | 12 | 8 | 0 | 12 | | 0 | 6 | 19 | 0 | **1533** |
| **Brong Ahafo** | Asunafo North | 4 | 7 | 6 | 5 | 11 | 1 | 10 | 2 | 6 | 9 | 17 | 16 | 3 | 4 | 1 | 3 | 3 | 2 | 0 | 3 | 0 | 2 | 3 | 0 | 0 | 0 | 0 | **118** |
| | Asunafo South | 0 | 6 | 5 | 4 | 9 | 2 | 10 | 0 | 3 | 5 | 9 | 11 | 7 | 3 | 0 | 0 | 1 | 0 | | 0 | 0 | 0 | 1 | 0 | 0 | 1 | 0 | **77** |
| | Asutifi | 3 | 5 | 4 | 0 | 3 | 0 | 14 | 0 | 0 | 4 | 3 | 7 | 4 | 3 | 1 | 2 | 1 | 0 | | 2 | 0 | 0 | 0 | 2 | 0 | 0 | 0 | **58** |
| | Atebubu-Amantin | 0 | 8 | 7 | 4 | 1 | 0 | 6 | 0 | 4 | 0 | 3 | 5 | 0 | 4 | 0 | 2 | 0 | 0 | 1 | 0 | 0 | 1 | 0 | 0 | 0 | 0 | 0 | **46** |
| | Berekum | 4 | 8 | 14 | 8 | 5 | 0 | 11 | 1 | 0 | 1 | 6 | 2 | 0 | 0 | | 4 | 0 | 0 | 0 | 0 | 0 | 0 | 0 | 0 | 0 | 0 | 0 | **64** |
| | Dormaa | 4 | 1 | 2 | 0 | 3 | 2 | 6 | 3 | 7 | 11 | 5 | 1 | 1 | | 0 | 0 | 0 | 0 | 2 | 1 | 0 | 2 | 4 | 2 | 0 | 2 | 0 | **59** |
| | Jaman North | 0 | 3 | 0 | 0 | 1 | 0 | 0 | 2 | 1 | 0 | 0 | 0 | | 0 | 0 | 0 | 0 | 0 | 0 | 0 | 0 | 0 | 0 | 0 | 0 | 0 | 0 | **7** |
| | Jaman South | 0 | 1 | 0 | 0 | 2 | 0 | 2 | 0 | 0 | 0 | 3 | 7 | 0 | 0 | 0 | 0 | 0 | 0 | 0 | 0 | 0 | 0 | 0 | 0 | 0 | 0 | 0 | **15** |
| | Kintampo North | 1 | 4 | 2 | 4 | 2 | 0 | 1 | 0 | 3 | 4 | 1 | 7 | 1 | 2 | 0 | 5 | 2 | 1 | 0 | 1 | 0 | 1 | 0 | 0 | 0 | 1 | 0 | **43** |
| | Kintampo South | 2 | 16 | 11 | 0 | 6 | 2 | 1 | 1 | 5 | 2 | 2 | 4 | 5 | 5 | 0 | 2 | 1 | 0 | 0 | 0 | 0 | 0 | 2 | 0 | 0 | 0 | 0 | **77** |
| | Nkoranza | 4 | 16 | 16 | 2 | 10 | 2 | 19 | 2 | 12 | 14 | 8 | 8 | 0 | 10 | 0 | 8 | 0 | 1 | 1 | 4 | 0 | 5 | 4 | 1 | 0 | 2 | 0 | **149** |
| | Pru | 5 | 10 | 0 | 2 | 2 | | 2 | 0 | 7 | 3 | 1 | 0 | 3 | 2 | 0 | 2 | 0 | 0 | 2 | 0 | 0 | 0 | 2 | 0 | 0 | 0 | 0 | **43** |
| | Sene | 10 | 14 | 4 | 3 | 5 | 0 | 10 | 0 | 14 | 0 | 12 | 2 | 1 | 5 | 0 | 3 | 0 | 1 | 4 | 0 | 0 | 0 | 0 | 0 | 1 | 0 | 0 | **89** |
| | Sunyani | 3 | 4 | 5 | | | 4 | 21 | 0 | 8 | 9 | 12 | 5 | 0 | 1 | 0 | 0 | 0 | 3 | 0 | 0 | 0 | 1 | 3 | 1 | 0 | 0 | 0 | **89** |
| | Tain | 2 | 6 | 21 | 2 | 9 | 0 | 42 | 1 | 12 | 18 | 11 | 26 | 14 | 2 | 0 | 12 | 2 | 0 | 2 | 5 | 0 | 0 | 1 | 0 | 0 | 2 | 0 | **200** |
| | Tano North | 1 | 2 | 3 | 2 | 5 | 0 | 9 | 0 | 0 | 1 | 1 | 4 | 0 | 0 | 0 | 0 | 0 | 1 | 0 | 0 | 0 | 0 | 0 | 0 | 0 | 0 | 0 | **29** |
| | Tano South | 1 | 8 | 6 | 5 | 10 | 0 | 0 | 0 | 1 | 4 | 0 | 1 | 0 | 0 | 0 | 2 | 0 | 0 | 0 | 0 | 0 | 1 | 0 | 0 | 0 | 0 | 0 | **39** |

C-UENR

*Daily Fire Detection count > 50 are highlighted in orange and Fire detection >=100 are highlighted in red.*

| Feb-2016 | | 01/02/16 | 02/02/16 | 03/02/16 | 04/02/16 | 05/02/16 | 06/02/16 | 07/02/16 | 08/02/16 | 09/02/16 | 10/02/16 | 11/02/16 | 12/02/16 | 13/02/16 | 14/02/16 | 15/02/16 | 16/02/16 | 17/02/16 | 19/02/16 | 20/02/16 | 21/02/16 | 22/02/16 | 23/02/16 | 25/02/16 | 26/02/16 | 7/02/16 | 28/02/16 | 29/02/16 | Monthly Total |
|---|---|---|---|---|---|---|---|---|---|---|---|---|---|---|---|---|---|---|---|---|---|---|---|---|---|---|---|---|---|
| **Brong Ahafo** | Techiman | 0 | 11 | 0 | 2 | 9 | 3 | 5 | 0 | 4 | 6 | 1 | 11 | 1 | 1 | 0 | 3 | 0 | 1 | 0 | 0 | 0 | 1 | 0 | 0 | 0 | 0 | 0 | **59** |
| | **Brong Ahafo Total** | 44 | 140 | 106 | 47 | 98 | 16 | 179 | 12 | 87 | 91 | 95 | 117 | 40 | 42 | 2 | 48 | 10 | 10 | 12 | 16 | 0 | 14 | 20 | 6 | 1 | 8 | 0 | **1261** |
| **Central** | Agona | 0 | 0 | 0 | 0 | 1 | 0 | 0 | 0 | 0 | 0 | 0 | 0 | 0 | 0 | 0 | 0 | 0 | 0 | 0 | 0 | | 0 | | 0 | 0 | 0 | 0 | **1** |
| | Ajumako-Enyan-Esiam | 0 | 0 | 0 | 0 | 0 | 0 | 0 | 0 | 1 | 0 | 0 | 0 | 0 | 0 | 0 | 0 | 0 | 0 | 0 | 0 | 0 | 1 | 0 | 0 | 0 | 0 | 0 | **2** |
| | Asikuma Odoben | 0 | 1 | 0 | 0 | 0 | 0 | 0 | 0 | 0 | 0 | 3 | 1 | 0 | 0 | 0 | 0 | 0 | 0 | | 0 | 0 | 2 | 1 | 0 | 0 | 0 | 0 | **8** |
| | Assin North | 0 | 2 | 0 | 0 | 0 | 0 | 0 | 0 | 3 | 0 | 0 | 0 | 0 | 2 | 0 | 0 | 0 | 0 | | 0 | 0 | 1 | 1 | 0 | 0 | 0 | 0 | **9** |
| | Assin South | 0 | 2 | 0 | 0 | 0 | 0 | 1 | 0 | 0 | 0 | 1 | 0 | 0 | 2 | 0 | 0 | 0 | 0 | 0 | 0 | 0 | 0 | 0 | 0 | 0 | 0 | 0 | **6** |
| | Awutu Efutu Senya | 0 | 0 | 0 | 0 | 0 | 0 | 0 | 0 | 0 | 0 | 0 | 1 | 0 | 0 | | 0 | 0 | 0 | 0 | 0 | 0 | 0 | 0 | 0 | 0 | 0 | 0 | **1** |
| | Gomoa | 0 | 3 | 0 | 0 | 1 | 0 | 1 | 0 | 1 | 0 | 0 | 0 | 2 | | 0 | 0 | 0 | 0 | 1 | 1 | 0 | 0 | 0 | 0 | 0 | 0 | 0 | **10** |
| | Komenda-Edina-Eguafo- | 0 | 1 | 0 | 0 | 5 | 0 | 0 | 0 | 1 | 0 | 0 | 0 | | 0 | 0 | 0 | 0 | 2 | 0 | 0 | 0 | 1 | 2 | 0 | 0 | 0 | 0 | **12** |
| | Lower Denkyira | 0 | 2 | 0 | 0 | 1 | 0 | 1 | 0 | 3 | 0 | 0 | 1 | 0 | 3 | 0 | 0 | 0 | 0 | 0 | 0 | 0 | 0 | 0 | 0 | 0 | 0 | 0 | **11** |
| | Mfantsiman | 0 | 1 | 0 | 0 | 1 | 0 | 0 | 0 | 1 | 0 | 0 | 0 | 0 | 1 | 0 | 0 | 0 | 0 | 2 | 0 | 0 | 1 | 0 | 0 | 0 | 0 | 0 | **7** |
| | Upper Denkyira | 0 | 1 | 0 | 0 | 0 | 0 | 1 | 0 | 0 | 0 | 0 | 0 | 0 | 0 | 0 | 0 | 0 | 0 | 0 | 0 | 0 | 1 | 1 | 0 | 0 | 0 | 0 | **4** |
| | **Central Total** | 0 | 13 | 0 | 0 | 9 | 0 | 4 | 0 | 10 | 0 | 4 | 3 | 2 | 8 | 0 | 0 | 0 | 2 | 3 | 1 | 0 | 7 | 5 | 0 | 0 | 0 | 0 | **71** |
| **Eastern** | Afram Plains | 2 | 25 | 3 | 13 | 15 | 1 | 12 | 0 | 11 | 6 | 42 | 12 | 0 | 2 | 0 | 2 | 0 | 2 | 0 | 0 | 0 | 1 | 3 | 0 | 0 | 0 | 0 | **152** |
| | Akwapim North | 0 | 1 | 1 | 0 | 2 | 0 | 4 | 0 | 4 | 1 | 0 | 1 | 0 | 0 | 0 | 0 | 0 | 0 | 1 | 0 | 0 | 1 | 1 | 0 | 0 | 0 | 0 | **17** |
| | Akwapim South | 0 | 2 | 0 | | | 0 | 0 | 0 | 0 | 0 | 1 | 1 | 0 | 0 | 0 | 0 | 0 | 0 | 0 | 0 | 0 | 0 | 0 | 0 | 0 | 0 | 0 | **5** |
| | Asuogyaman | 0 | | 0 | 0 | 0 | 0 | 1 | 0 | 0 | 0 | 5 | 0 | 0 | 0 | 0 | 1 | 0 | 0 | 0 | 0 | 0 | 0 | 0 | 0 | 0 | 0 | 0 | **8** |
| | Atiwa | 0 | 1 | 0 | 0 | 1 | 0 | 0 | 0 | 0 | 0 | 0 | 0 | 0 | 0 | 0 | 0 | 0 | 0 | 0 | 0 | 0 | 1 | 0 | 0 | 0 | 0 | 0 | **3** |
| | Birim North | 0 | 0 | 0 | 0 | 0 | 0 | 0 | 0 | 0 | 0 | 1 | 0 | 0 | 0 | 0 | 0 | 0 | 0 | 0 | 1 | 0 | 6 | 2 | 0 | 0 | 0 | 0 | **10** |

C-UENR

*Daily Fire Detection count > 50 are highlighted in orange and Fire detection >=100 are highlighted in red.*

| Feb-2016 | | 01/02/16 | 02/02/16 | 03/02/16 | 04/02/16 | 05/02/16 | 06/02/16 | 07/02/16 | 08/02/16 | 09/02/16 | 10/02/16 | 11/02/16 | 12/02/16 | 13/02/16 | 14/02/16 | 15/02/16 | 16/02/16 | 17/02/16 | 19/02/16 | 20/02/16 | 21/02/16 | 22/02/16 | 23/02/16 | 25/02/16 | 26/02/16 | 7/02/16 | 28/02/16 | 29/02/16 | Monthly Total |
|---|---|---|---|---|---|---|---|---|---|---|---|---|---|---|---|---|---|---|---|---|---|---|---|---|---|---|---|---|---|
| **Eastern** | Birim South | 0 | 2 | 0 | 0 | 1 | 0 | 3 | 0 | 1 | 0 | 2 | 3 | 0 | 1 | 0 | 0 | 0 | 0 | 1 | 0 | 0 | 1 | 5 | 0 | 0 | 0 | 0 | **20** |
| | East Akim | 0 | 0 | 0 | 0 | 1 | 1 | 0 | 0 | 1 | 0 | 2 | 0 | 0 | 0 | 0 | 0 | 0 | 0 | 0 | 0 | 0 | 0 | 0 | 0 | 0 | 0 | 1 | **6** |
| | Fanteakwa | 2 | 8 | 2 | 3 | 3 | 1 | 1 | 0 | 0 | 3 | 2 | 1 | 0 | 0 | 0 | 0 | 0 | 0 | 0 | 0 | | 0 | | 0 | 0 | 0 | 1 | **27** |
| | Kwabibirem | 0 | 1 | 0 | 0 | 0 | 0 | 1 | 0 | 0 | 0 | 2 | 0 | 0 | 0 | 0 | 0 | 0 | 0 | 0 | 0 | 0 | 3 | 0 | 0 | 0 | 0 | 0 | **7** |
| | Kwahu South | 4 | 23 | 11 | 0 | 5 | 3 | 2 | 0 | 3 | 11 | 13 | 0 | 0 | 0 | 0 | 0 | 0 | 0 | 3 | 0 | 0 | 0 | 0 | 0 | 0 | 0 | 0 | **78** |
| | Kwahu West | 0 | 7 | 0 | 0 | 1 | 0 | 0 | 0 | 0 | 0 | 0 | 0 | 0 | 0 | 0 | 0 | 0 | 0 | | 0 | 2 | 0 | 0 | 0 | 0 | 0 | 0 | **10** |
| | Manya Krobo | 0 | 17 | 2 | 0 | 0 | 1 | 3 | 0 | 1 | 1 | 1 | 0 | 0 | 0 | 0 | 0 | 0 | 0 | 1 | 0 | 0 | 1 | 0 | 0 | 1 | 0 | 0 | **29** |
| | New Juaben | 2 | 3 | 0 | 0 | 0 | 0 | 0 | 0 | 0 | 0 | 0 | 0 | 0 | 0 | | 0 | 0 | 0 | 0 | 0 | 0 | 0 | 0 | 0 | 0 | 0 | 0 | **5** |
| | Suhum Kraboa | 0 | 3 | 0 | 0 | 1 | 0 | 0 | 0 | 4 | 6 | 4 | 0 | 0 | | 0 | 0 | 0 | 0 | 0 | 0 | 0 | 1 | 0 | 0 | 0 | 0 | 0 | **19** |
| | West Akim | 0 | 0 | 0 | 0 | 1 | 0 | 4 | 0 | 1 | 0 | 0 | 0 | | 0 | 0 | 0 | 0 | 0 | 0 | 0 | 0 | 1 | 1 | 0 | 0 | 0 | 0 | **8** |
| | Yilo Krobo | 0 | 10 | 2 | 0 | 1 | 0 | 1 | 0 | 2 | 3 | 4 | 1 | 0 | 0 | 0 | 0 | 0 | 0 | 0 | 0 | 0 | 0 | 0 | 0 | 0 | 0 | 2 | **26** |
| | **Eastern Total** | 10 | 104 | 21 | 16 | 33 | 7 | 32 | 0 | 28 | 31 | 79 | 19 | 0 | 3 | 0 | 3 | 0 | 2 | 6 | 1 | 2 | 16 | 12 | 0 | 1 | 0 | 4 | **430** |
| **Greater Accra** | Dangbe East | 0 | 0 | 0 | 3 | 0 | 1 | 0 | 0 | 1 | 0 | 0 | 1 | 0 | 0 | 0 | 0 | 0 | 0 | 0 | 0 | 0 | 0 | 0 | 0 | 0 | 0 | 0 | **6** |
| | Dangbe West | 0 | 0 | 0 | 0 | 1 | 0 | 1 | | 0 | 0 | 0 | 0 | 0 | 0 | 0 | 0 | 0 | 0 | 0 | 0 | 0 | 0 | 0 | 0 | 0 | 0 | 0 | **2** |
| | Ga West | 0 | 0 | 0 | 2 | 0 | | 0 | 0 | 1 | 0 | 0 | 0 | 0 | 0 | 0 | 0 | 0 | 0 | 0 | 0 | 0 | 0 | 0 | 0 | 0 | 0 | 0 | **3** |
| | Tema | 0 | 0 | 0 | 1 | 0 | 0 | 0 | 0 | 0 | 0 | 0 | 0 | 0 | 0 | 0 | 0 | 0 | 0 | 0 | 0 | 0 | 0 | 0 | 0 | 0 | 0 | 0 | **1** |
| | **Greater Accra Total** | 0 | 0 | 0 | 6 | | 1 | 1 | 0 | 2 | 0 | 0 | 1 | 0 | 0 | 0 | 0 | 0 | 0 | 0 | 0 | 0 | 0 | 0 | 0 | 0 | 0 | 0 | **12** |
| **Northern** | Bole | 0 | | 2 | 0 | 2 | 0 | 4 | 0 | 0 | 3 | 2 | 1 | 0 | 0 | 0 | 1 | 1 | 0 | 0 | 0 | 0 | 0 | 0 | 0 | 0 | 0 | 0 | **18** |
| | Bunkpurugu Yunyoo | 0 | 4 | 2 | 0 | 1 | 0 | 3 | 0 | 0 | 0 | 0 | 0 | 0 | 2 | 0 | 2 | 0 | 0 | 3 | 0 | 0 | 2 | 1 | 0 | 0 | 0 | 0 | **20** |
| | Central Gonja | 0 | 9 | 2 | 1 | 13 | 0 | 16 | 0 | 6 | 3 | 3 | 1 | 9 | 7 | 0 | 5 | 1 | 0 | 1 | 0 | 0 | 1 | 1 | 2 | 2 | 0 | 0 | **83** |

C-UENR

*Daily Fire Detection count > 50 are highlighted in orange and Fire detection >=100 are highlighted in red.*

| Feb-2016 | | 01/02/16 | 02/02/16 | 03/02/16 | 04/02/16 | 05/02/16 | 06/02/16 | 07/02/16 | 08/02/16 | 09/02/16 | 10/02/16 | 11/02/16 | 12/02/16 | 13/02/16 | 14/02/16 | 15/02/16 | 16/02/16 | 17/02/16 | 19/02/16 | 20/02/16 | 21/02/16 | 22/02/16 | 23/02/16 | 25/02/16 | 26/02/16 | 7/02/16 | 28/02/16 | 29/02/16 | Monthly Total |
|---|---|---|---|---|---|---|---|---|---|---|---|---|---|---|---|---|---|---|---|---|---|---|---|---|---|---|---|---|---|
| **Northern** | East Gonja | 3 | 31 | 6 | 11 | 9 | 6 | 27 | 0 | 23 | 4 | 13 | 3 | 4 | 25 | 7 | 6 | 0 | 0 | 3 | 0 | 0 | 1 | 6 | 2 | 1 | 4 | 3 | **198** |
| | East Mamprusi | 0 | 0 | 0 | 0 | 0 | 0 | 2 | 0 | 1 | 0 | 0 | 0 | 0 | 1 | 0 | 2 | 0 | 0 | 0 | 0 | 0 | 0 | 0 | 0 | 0 | 0 | 0 | **6** |
| | Gushiegu | 1 | 15 | 14 | 10 | 6 | 2 | 6 | 1 | 2 | 12 | 17 | 0 | 0 | 5 | 5 | 2 | 5 | 1 | 3 | 0 | | 1 | | 0 | 1 | 1 | 0 | **110** |
| | Karaga | 1 | 10 | 5 | 0 | 0 | 3 | 13 | 0 | 9 | 4 | 0 | 0 | 0 | 1 | 0 | 0 | 0 | 0 | 0 | 0 | 0 | 0 | 0 | 0 | 0 | 0 | 0 | **46** |
| | Nanumba North | 0 | 21 | 1 | 3 | 2 | 8 | 6 | 0 | 8 | 3 | 3 | 0 | 6 | 3 | 0 | 0 | 0 | 0 | | 0 | 0 | 0 | 1 | 0 | 6 | 0 | 0 | **73** |
| | Nanumba South | 2 | 7 | 2 | 8 | 1 | 2 | 5 | 0 | 1 | 0 | 10 | 0 | 4 | 1 | 0 | 1 | 0 | 0 | | 2 | 0 | 0 | 5 | 0 | 0 | 2 | 1 | **56** |
| | Saboba Chereponi | 5 | 8 | 3 | 5 | 3 | 16 | 17 | 1 | 4 | 7 | 0 | 5 | 4 | 10 | 0 | 1 | 1 | 0 | 0 | 8 | 0 | 5 | 6 | 0 | 2 | 1 | 0 | **112** |
| | Savelugu Nanton | 0 | 0 | 0 | 0 | 1 | 1 | 3 | 0 | 1 | 0 | 0 | 0 | 0 | 0 | | 0 | 0 | 0 | 0 | 0 | 0 | 0 | 0 | 0 | 0 | 0 | 0 | **6** |
| | Sawa-Tuna-Kalba | 0 | 6 | 9 | 0 | 1 | 0 | 7 | 0 | 1 | 1 | 1 | 3 | 0 | 1 | 0 | 2 | 0 | 0 | 1 | 1 | 0 | 0 | 4 | 0 | 0 | 0 | 0 | **38** |
| | Tolon-Kumbungu | 0 | 1 | 3 | 0 | 0 | 0 | 2 | 0 | 4 | 1 | 0 | 0 | | 3 | 0 | 3 | 0 | 0 | 1 | 0 | 0 | 0 | 0 | 0 | 0 | 0 | 0 | **18** |
| | West Gonja | 10 | 19 | 17 | 0 | 6 | 7 | 19 | 4 | 10 | 9 | 7 | 6 | 10 | 3 | 0 | 18 | 1 | 0 | 6 | 1 | 0 | 0 | 2 | 0 | 7 | 6 | 0 | **168** |
| | West Mamprusi | 0 | 7 | 2 | 2 | 2 | 3 | 12 | 0 | 4 | 0 | 0 | 3 | 2 | 1 | 0 | 1 | 0 | 0 | 2 | 0 | 0 | 0 | 0 | 0 | 2 | 1 | 0 | **44** |
| | Yendi | 3 | 16 | 4 | 5 | 11 | 21 | 22 | | 14 | 4 | 6 | 3 | 3 | 7 | 1 | 2 | 3 | 1 | 0 | 1 | 2 | 0 | 2 | 0 | 1 | 0 | 2 | **140** |
| | Zabzugu Tatale | 5 | 6 | 0 | 5 | 0 | 0 | 20 | | 0 | 3 | 3 | 2 | 0 | 1 | 0 | 4 | 0 | 0 | 1 | 1 | 0 | 0 | 4 | 0 | 2 | 0 | 0 | **57** |
| | **Northern Total** | 30 | 162 | 72 | 50 | 58 | 69 | 184 | 12 | 88 | 54 | 65 | 27 | 42 | 71 | 13 | 50 | 12 | 2 | 25 | 14 | 2 | 10 | 32 | 4 | 24 | 15 | 6 | **1193** |
| **Upper East** | Bawku Municipal | 0 | 0 | 0 | 0 | 0 | 0 | 0 | 1 | 1 | 0 | 0 | 0 | 0 | 0 | 0 | 0 | 0 | 0 | 1 | 0 | 0 | 0 | 0 | 0 | 0 | 0 | 0 | **3** |
| | Bawku West | 1 | 2 | 0 | | | 0 | 0 | 0 | 0 | 0 | 0 | 0 | 0 | 0 | 0 | 0 | 0 | 0 | 0 | 0 | 0 | 0 | 0 | 0 | 0 | 0 | 0 | **4** |
| | Bolgatanga | 0 | 0 | 0 | 0 | 0 | 0 | 1 | 0 | 0 | 0 | 1 | 0 | 0 | 0 | 0 | 0 | 0 | 0 | 0 | 0 | 0 | 0 | 0 | 0 | 0 | 0 | 0 | **2** |
| | Bongo | 0 | 0 | 0 | 0 | 0 | 0 | 0 | 0 | 1 | 0 | 0 | 0 | 0 | 1 | 0 | 0 | 0 | 0 | 0 | 0 | 0 | 0 | 0 | 0 | 0 | 0 | 0 | **2** |
| | Builsa | 0 | 1 | 0 | 0 | 0 | 0 | 0 | 0 | 0 | 0 | 0 | 0 | 0 | 2 | 0 | 0 | 0 | 0 | 0 | 1 | 0 | 0 | 2 | 0 | 0 | 0 | 0 | **6** |

*Daily Fire Detection count > 50 are highlighted in orange and Fire detection >=100 are highlighted in red.*

| Feb-2016 | | 01/02/16 | 02/02/16 | 03/02/16 | 04/02/16 | 05/02/16 | 06/02/16 | 07/02/16 | 08/02/16 | 09/02/16 | 10/02/16 | 11/02/16 | 12/02/16 | 13/02/16 | 14/02/16 | 15/02/16 | 16/02/16 | 17/02/16 | 19/02/16 | 20/02/16 | 21/02/16 | 22/02/16 | 23/02/16 | 25/02/16 | 26/02/16 | 7/02/16 | 28/02/16 | 29/02/16 | Monthly Total |
|---|---|---|---|---|---|---|---|---|---|---|---|---|---|---|---|---|---|---|---|---|---|---|---|---|---|---|---|---|---|
| **Upper East** | Kassena Nankana | 0 | 1 | 0 | 0 | 0 | 0 | 0 | 0 | 0 | 0 | 0 | 0 | 0 | 1 | 0 | 0 | 0 | 0 | 1 | 0 | 0 | 0 | 0 | 3 | 0 | 1 | 0 | **7** |
| | Talensi Nabdam | 0 | 0 | 0 | 0 | 1 | 0 | 0 | 0 | 0 | 0 | 0 | 0 | 0 | 0 | 0 | 0 | 0 | 0 | 0 | 0 | 0 | 0 | 1 | 0 | 1 | 0 | 0 | **3** |
| | **Upper East Total** | 1 | 4 | 0 | 0 | 2 | 0 | 1 | 1 | 2 | 0 | 1 | 0 | 0 | 4 | 0 | 0 | 0 | 0 | 2 | 1 | 0 | 0 | 3 | 3 | 1 | 1 | 0 | **27** |
| **Upper West** | Jirapa Lambussie | 0 | 0 | 0 | 0 | 1 | 0 | 1 | 0 | 0 | 0 | 0 | 0 | 0 | 0 | 0 | 0 | 0 | 0 | 0 | 0 | 0 | 0 | 0 | 0 | 0 | 0 | 0 | **2** |
| | Lawra | 0 | 0 | 0 | 0 | 0 | 0 | 1 | 0 | 0 | 0 | 0 | 0 | 0 | 0 | 0 | 0 | 0 | 0 | | 0 | 0 | 0 | 0 | 0 | 0 | 0 | 0 | **1** |
| | Nadowli | 0 | 1 | 1 | 0 | 0 | 0 | 2 | 0 | 1 | 0 | 0 | 0 | 0 | 0 | 0 | 0 | 0 | 0 | | 0 | 0 | 0 | 0 | 0 | 0 | 0 | 0 | **5** |
| | Sissala East | 1 | 4 | 0 | 1 | 1 | 0 | 1 | 0 | 5 | 2 | 0 | 0 | 0 | 3 | 0 | 3 | 0 | 0 | 0 | 0 | 0 | 0 | 5 | 0 | 0 | 0 | 0 | **26** |
| | Sissala West | 0 | 1 | 1 | 2 | 1 | 0 | 1 | 0 | 0 | 0 | 1 | 0 | 0 | 1 | | 0 | 0 | 0 | 0 | 0 | 0 | 0 | 1 | 0 | 2 | 0 | 0 | **11** |
| | Wa | 1 | 3 | 1 | 0 | 3 | 0 | 2 | 0 | 4 | 0 | 1 | 1 | 0 | | 0 | 0 | 0 | 0 | 0 | 1 | 0 | 0 | 0 | 0 | 0 | 0 | 0 | **17** |
| | Wa East | 2 | 4 | 2 | 0 | 7 | 0 | 4 | 0 | 1 | 0 | 3 | 2 | | 0 | 0 | 2 | 0 | 0 | 2 | 1 | 0 | 0 | 0 | 0 | 0 | 0 | 0 | **30** |
| | Wa West | 0 | 0 | 0 | 0 | 0 | 0 | 1 | 0 | 0 | 0 | 0 | 0 | 0 | 0 | 0 | 0 | 0 | 0 | 0 | 1 | 0 | 0 | 0 | 0 | 0 | 0 | 0 | **2** |
| | **Upper West Total** | 4 | 13 | 5 | 3 | 13 | 0 | 13 | 0 | 11 | 2 | 5 | 3 | 0 | 4 | 0 | 5 | 0 | 0 | 2 | 3 | 0 | 0 | 6 | 0 | 2 | 0 | 0 | **94** |
| **Volta** | Adaklu Anyigbe | 0 | 1 | 0 | 0 | 2 | 1 | 4 | 0 | 4 | 0 | 13 | 1 | 0 | 2 | 0 | 0 | 0 | 4 | 1 | 0 | 0 | 0 | 0 | 0 | 0 | 0 | 0 | **33** |
| | Akatsi | 0 | 0 | 0 | 0 | 1 | 0 | 0 | | 7 | 0 | 1 | 0 | 0 | 2 | 0 | 0 | 0 | 0 | 0 | 0 | 0 | 0 | 1 | 0 | 0 | 0 | 0 | **12** |
| | Ho | 0 | 11 | 0 | 1 | 0 | | 4 | 0 | 15 | 0 | 12 | 1 | 0 | 0 | 0 | 0 | 0 | 0 | 0 | 0 | 0 | 0 | 0 | 0 | 0 | 0 | 0 | **48** |
| | Hohoe | 0 | 18 | 0 | 2 | 4 | 3 | 15 | 0 | 10 | 8 | 35 | 9 | 1 | 0 | 0 | 1 | 0 | 0 | 1 | 0 | 0 | 2 | 0 | 0 | 0 | 0 | 0 | **109** |
| | Jasikan | 10 | 33 | 9 | | | 7 | 16 | 0 | 19 | 15 | 40 | 22 | 16 | 5 | 2 | 2 | 0 | 0 | 1 | 0 | 0 | 3 | 1 | 0 | 0 | 2 | 0 | **212** |
| | Kadjebi | 0 | 5 | 0 | 9 | 4 | 3 | 6 | 0 | 20 | 12 | 26 | 10 | 9 | 2 | 0 | 0 | 0 | 0 | 0 | 0 | 0 | 1 | 0 | 0 | 0 | 0 | 0 | **107** |
| | Ketu | 0 | 1 | 0 | 0 | 1 | 0 | 0 | 0 | 0 | 0 | 0 | 0 | 0 | 0 | 0 | 0 | 0 | 0 | 0 | 0 | 0 | 2 | 0 | 0 | 0 | 0 | 0 | **4** |
| | Kpandu | 0 | 13 | 0 | 0 | 4 | 3 | 11 | 0 | 6 | 3 | 20 | 2 | 1 | 1 | 0 | 2 | 0 | 0 | 2 | 0 | 0 | 0 | 0 | 0 | 0 | 0 | 0 | **68** |

C - UENR

*Daily Fire Detection count > 50 are highlighted in orange and Fire detection >=100 are highlighted in red.*

| Feb-2016 | | 01/02/16 | 02/02/16 | 03/02/16 | 04/02/16 | 05/02/16 | 06/02/16 | 07/02/16 | 08/02/16 | 09/02/16 | 10/02/16 | 11/02/16 | 12/02/16 | 13/02/16 | 14/02/16 | 15/02/16 | 16/02/16 | 17/02/16 | 19/02/16 | 20/02/16 | 21/02/16 | 22/02/16 | 23/02/16 | 25/02/16 | 26/02/16 | 7/02/16 | 28/02/16 | 29/02/16 | Monthly Total |
|---|---|---|---|---|---|---|---|---|---|---|---|---|---|---|---|---|---|---|---|---|---|---|---|---|---|---|---|---|---|
| **Volta** | Krachi | 1 | 1 | 0 | 1 | 0 | 0 | 1 | 0 | 1 | 0 | 1 | 2 | 0 | 1 | 0 | 4 | 0 | 0 | 1 | 0 | 0 | 0 | 0 | 0 | 2 | 0 | 0 | **16** |
| | Krachi East | 0 | 5 | 0 | 5 | 2 | 2 | 3 | 0 | 2 | 1 | 2 | 1 | 2 | 0 | 0 | 0 | 0 | 0 | 0 | 0 | 0 | 1 | 1 | 0 | 0 | 0 | 0 | **27** |
| | Nkwanta | 1 | 30 | 3 | 7 | 10 | 14 | 25 | 0 | 20 | 7 | 12 | 3 | 6 | 7 | 0 | 11 | 0 | 0 | 3 | 0 | | 3 | 2 | 0 | 1 | 0 | 0 | **165** |
| | North Tongu | 0 | 0 | 0 | 0 | 2 | 0 | 2 | 0 | 4 | 1 | 6 | 0 | 0 | 0 | 0 | 0 | 0 | 0 | 0 | 0 | 0 | 0 | 1 | 0 | 0 | 0 | 0 | **16** |
| | South Dayi | 0 | 2 | 0 | 0 | 0 | 0 | 2 | 0 | 4 | 0 | 4 | 0 | 0 | 0 | 0 | 0 | 0 | 0 | | 0 | 0 | 0 | 0 | 0 | 0 | 2 | 0 | **14** |
| | South Tongu | 0 | 0 | 0 | 0 | 0 | 0 | 0 | 0 | 0 | 0 | 0 | 0 | 1 | 0 | 0 | 0 | 0 | 0 | | 0 | 0 | 0 | 0 | 0 | 0 | 0 | 0 | **1** |
| | **Volta Total** | 12 | 120 | 12 | 29 | 35 | 37 | 89 | 0 | 112 | 47 | 172 | 51 | 36 | 20 | 2 | 20 | 0 | 4 | 9 | 0 | 0 | 12 | 6 | 0 | 3 | 4 | 0 | **832** |
| **Western** | Ahanta West | 0 | 1 | 0 | 0 | 0 | 0 | 0 | 0 | 0 | 0 | 0 | 0 | 0 | 0 | | 0 | 0 | 1 | 0 | 2 | 0 | 0 | 0 | 0 | 0 | 0 | 0 | **4** |
| | Aowin-Suaman | 0 | 0 | 0 | 0 | 3 | 0 | 0 | 0 | 0 | 1 | 0 | 0 | 0 | 1 | 0 | 0 | 0 | 0 | 0 | 1 | 0 | 3 | 1 | 0 | 0 | 1 | 0 | **11** |
| | Bia | 0 | 1 | 0 | 0 | 0 | 0 | 0 | 0 | 0 | 1 | 2 | 2 | | 0 | 0 | 1 | 0 | 0 | 2 | 0 | 0 | 0 | 0 | 0 | 0 | 0 | 0 | **9** |
| | Bibiani Anhwiaso | 0 | 9 | 1 | 0 | 11 | 0 | 4 | 0 | 2 | 6 | 5 | 2 | 0 | 0 | 0 | 0 | 0 | 0 | 0 | 1 | 0 | 1 | 0 | 0 | 0 | 0 | 0 | **42** |
| | Jomoro | 0 | 0 | 0 | 0 | 0 | 0 | 0 | 0 | 0 | 2 | 0 | 0 | 0 | 0 | 0 | 0 | 0 | 3 | 0 | 0 | 0 | 2 | 1 | 0 | 0 | 0 | 0 | **8** |
| | Juabeso | 1 | 8 | 7 | 3 | 12 | 0 | 2 | 0 | 5 | 9 | 10 | 21 | 0 | 1 | 0 | 0 | 0 | 1 | 0 | 2 | 0 | 0 | 0 | 0 | 0 | 0 | 0 | **92** |
| | Mpohor Wassa East | 0 | 1 | 1 | 0 | 0 | 0 | 0 | | 0 | 0 | 4 | 0 | 0 | 1 | 0 | 0 | 0 | 0 | 0 | 0 | 0 | 2 | 3 | 0 | 0 | 0 | 0 | **12** |
| | Nzema East | 0 | 0 | 0 | 1 | 0 | | 0 | 0 | 1 | 0 | 0 | 1 | 0 | 1 | 0 | 0 | 1 | 1 | 0 | 0 | 0 | 0 | 0 | 0 | 0 | 0 | 0 | **6** |
| | Sefwi Wiawso | 0 | 1 | 1 | 0 | 2 | 0 | 0 | 0 | 4 | 2 | 5 | 2 | 0 | 0 | 0 | 3 | 0 | 0 | 0 | 0 | 0 | 2 | 3 | 0 | 0 | 0 | 0 | **25** |
| | Shama Ahanta East | 0 | 1 | 0 | | | 0 | 3 | 0 | 1 | 0 | 0 | 0 | 0 | 0 | 0 | 0 | 0 | 0 | 0 | 0 | 0 | 1 | 0 | 0 | 0 | 0 | 0 | **6** |
| | Wasa Amenfi East | 0 | 0 | 0 | 0 | 1 | 0 | 3 | 0 | 0 | 0 | 0 | 1 | 0 | 1 | 0 | 0 | 0 | 0 | 0 | 0 | 0 | 1 | 0 | 0 | 0 | 0 | 0 | **7** |
| | Wasa Amenfi West | 0 | 0 | 0 | 0 | 1 | 0 | 2 | 0 | 2 | 0 | 0 | 3 | 0 | 0 | 0 | 0 | 0 | 0 | 0 | 0 | 0 | 4 | 2 | 0 | 0 | 0 | 0 | **14** |

C-UENR

*Daily Fire Detection count > 50 are highlighted in orange an  Fi e detec ion >=100 are highlighted in red.*

| Feb-2016 | | 01/02/16 | 02/02/16 | 03/02/16 | 04/02/16 | 05/02/16 | 06/02/16 | 07/02/16 | 08/02/16 | 09/02/16 | 10/02/16 | 11/02/16 | 12/02/16 | 13/02/16 | 14/ 2 16 | 15 02/16 | 16/02/16 | 17/02 16 | 19/02/16 | 20/02/16 | 21/02/16 | 22/02/16 | 23/02/16 | 25/02/16 | 26/02/16 | 27/02/16 | 28/02/16 | 29/02/16 | **Monthly Total** |
|---|---|---|---|---|---|---|---|---|---|---|---|---|---|---|---|---|---|---|---|---|---|---|---|---|---|---|---|---|---|
| **Western** | Wassa West | 0 | 4 | 0 | 0 | 0 | 0 | 4 | 0 | 9 | 2 | 1 | | 0 | 3 | 0 | 0 | 0 | 0 | 0 | 0 | 0 | 5 | 5 | 0 | 0 | 2 | 0 | **40** |
| | **Western Total** | 1 | 26 | 10 | 4 | 30 | 0 | 28 | 0 | 24 | 23 | 27 | 37 | 0 | 8 | 0 | 4 | 1 | 6 | 2 | 6 | 0 | 21 | 15 | 0 | 0 | 3 | 0 | **276** |
| **Provincial Total** | | **133** | **818** | **392** | **215** | **479** | **184** | **725** | **38** | **446** | **404** | **565** | **341** | **133** | **194** | **17** | **145** | **24** | **38** | **73** | **50** | **4** | **92** | **108** | **13** | **38** | **50** | **10** | **5729** |

*Daily Fire Detection count > 50 are highlighted in orange and Fire detection >=100 are highlighted in red.*

| Mar-2016 | | 01/03/16 | 02/03/16 | 03/03/16 | 04/03/16 | 05/03/16 | 06/03/16 | 07/03/16 | 08/03/16 | 09/03/16 | 10/03/16 | 11/03/16 | 12/03/16 | 13/03/16 | 15/03/16 | 16/03/16 | 17/03/16 | 19/03/16 | 20/03/16 | 21/03/16 | 22/03/16 | 23/03/16 | 24/03/16 | 26/03/16 | 27/03/16 | 28/03/16 | 29/03/16 | 31/03/16 | Monthly Total |
|---|---|---|---|---|---|---|---|---|---|---|---|---|---|---|---|---|---|---|---|---|---|---|---|---|---|---|---|---|---|
| **Ashanti** | Adansi North | 0 | 0 | 0 | 0 | 1 | 0 | 0 | 0 | 0 | 0 | 0 | 0 | 0 | 0 | 0 | 0 | 0 | 0 | 0 | 0 | 0 | 0 | 0 | 0 | 0 | 0 | 0 | **1** |
| | Adansi South | 2 | 0 | 8 | 0 | 2 | 0 | 0 | 0 | 0 | 2 | 0 | 1 | 0 | 0 | 0 | 0 | 0 | 0 | 0 | 0 | 0 | 0 | 1 | 0 | 0 | 0 | 0 | **16** |
| | Afigya Sekyere | 0 | 0 | 6 | 0 | 0 | 0 | 0 | 0 | 0 | 0 | 0 | 1 | 0 | 0 | 0 | 0 | 0 | 0 | 0 | 0 | 0 | 0 | 0 | 0 | 0 | 0 | 0 | **7** |
| | Ahafo Ano North | 0 | 0 | 3 | 0 | 1 | 0 | 0 | 0 | 0 | 1 | 0 | 1 | 0 | 0 | 0 | 0 | 0 | 0 | 0 | 0 | 0 | 0 | 0 | 0 | 0 | 0 | 0 | **6** |
| | Ahafo Ano South | 0 | 0 | 10 | 3 | 3 | 0 | 0 | 0 | 0 | 0 | 0 | 0 | 0 | 0 | 0 | 0 | 0 | [illegible] | 1 | 1 | 0 | 0 | 0 | 1 | 0 | 0 | 0 | **19** |
| | Amansie Central | 0 | 0 | 0 | 0 | 0 | 0 | 0 | 0 | 0 | 2 | 0 | 1 | 0 | 0 | 0 | 0 | 0 | 0 | 0 | 0 | 0 | 0 | 0 | 0 | 0 | 0 | 0 | **3** |
| | Amansie East | 0 | 0 | 4 | 0 | 0 | 0 | 0 | 0 | 0 | 0 | 0 | 0 | 0 | 0 | 0 | 0 | 0 | 0 | 0 | 0 | 0 | 0 | 0 | 0 | 0 | 0 | 0 | **4** |
| | Amansie West | 0 | 0 | 2 | 0 | 0 | 0 | 0 | 1 | 0 | 1 | 0 | 0 | 0 | 0 | 0 | 0 | 0 | 0 | 0 | 0 | 0 | 0 | 0 | 0 | 0 | 0 | 0 | **4** |
| | Asante Akim North | 0 | 0 | 3 | 0 | 1 | 0 | 1 | 0 | 0 | 0 | 0 | 0 | 0 | 0 | 0 | 0 | 0 | 0 | 1 | 0 | 0 | 0 | 0 | 0 | 0 | 0 | 0 | **6** |
| | Asante Akim South | 0 | 0 | 1 | 0 | 0 | 0 | 0 | 1 | 0 | 0 | 0 | 1 | 0 | [illegible] | 0 | 0 | 0 | 0 | 0 | 0 | 0 | 0 | 0 | 0 | 0 | 0 | 0 | **3** |
| | Atwima | 0 | 0 | 7 | 0 | 1 | 0 | 0 | 0 | 0 | 0 | 0 | 1 | 0 | 0 | 0 | 0 | 0 | 0 | 0 | 1 | 0 | 0 | 0 | 0 | 0 | 0 | 0 | **10** |
| | Atwima Mponua | 0 | 0 | 2 | 0 | 2 | 0 | 0 | [illegible] | 0 | 1 | 0 | 1 | 0 | 0 | 0 | 0 | 0 | 0 | 0 | 0 | 0 | 0 | 1 | 0 | 0 | 0 | 0 | **7** |
| | Bosomtwe-Kwanwoma | 0 | 0 | 1 | 0 | 0 | 0 | 0 | 2 | 0 | [illegible] | 0 | 1 | 0 | 2 | 0 | 0 | 0 | 0 | 0 | 0 | 0 | 0 | 2 | 0 | 0 | 0 | 0 | **8** |
| | Ejisu-Juabeng | 0 | 0 | 3 | 0 | 0 | 0 | 0 | 0 | 0 | 2 | 0 | 0 | 0 | 0 | 0 | 0 | 0 | 0 | 0 | 1 | 0 | 0 | 0 | 0 | 0 | 0 | 0 | **6** |
| | Ejura Sekyedumas | 0 | 0 | 0 | 0 | 2 | 0 | 0 | 2 | 0 | 0 | 0 | 0 | 0 | 0 | 0 | 0 | 0 | 0 | 0 | 0 | 0 | 0 | 0 | 0 | 0 | 0 | 0 | **4** |
| | Kwabre | 0 | 0 | 4 | 0 | 0 | 0 | 0 | 1 | 0 | 1 | 0 | 0 | 0 | 0 | 0 | 0 | 0 | 0 | 0 | 0 | 0 | 0 | 0 | 0 | 0 | 0 | 0 | **6** |
| | Offinso | 0 | 0 | 16 | 0 | 3 | 0 | 1 | 1 | 0 | 0 | 0 | 0 | 0 | 0 | 0 | 0 | 0 | 0 | 0 | 0 | 0 | 0 | 1 | 0 | 0 | 0 | 0 | **22** |
| | Sekyere East | 0 | 0 | 15 | 2 | 0 | 0 | 0 | 4 | 0 | 5 | 0 | 0 | 0 | 0 | 0 | 0 | 0 | 0 | 3 | 0 | 0 | 0 | 0 | 0 | 0 | 0 | 0 | **29** |

C-UENR

*Daily Fire Detection count > 50 are highlighted in orange and Fire detection >=100 are highlighted in red.*

| Mar-2016 | | 01/03/16 | 02/03/16 | 03/03/16 | 04/03/16 | 05/03/16 | 06/03/16 | 07/03/16 | 08/03/16 | 09/03/16 | 10/03/16 | 11/03/16 | 12/03/16 | 13/03/16 | 15/03/16 | 16/03/16 | 17/03/16 | 19/03/16 | 20/03/16 | 21/03/16 | 22/03/16 | 23/03/16 | 24/03/16 | 26/03/16 | 27/03/16 | 28/03/16 | 29/03/16 | 31/03/16 | Monthly Total |
|---|---|---|---|---|---|---|---|---|---|---|---|---|---|---|---|---|---|---|---|---|---|---|---|---|---|---|---|---|---|
| **Ashanti** | Sekyere West | 0 | 0 | 1 | 0 | 1 | 0 | 0 | 0 | 0 | 1 | 0 | 0 | 0 | 0 | 0 | 0 | 0 | 0 | 0 | 0 | 0 | 0 | 0 | 0 | 0 | 0 | 0 | **3** |
| | **Ashanti Total** | 2 | 0 | 86 | 5 | 17 | 0 | 2 | 12 | 0 | 16 | 0 | 8 | 0 | 2 | 0 | 0 | 0 | 0 | 5 | 3 | 0 | 0 | 5 | 1 | 0 | 0 | 0 | **164** |
| **Brong Ahafo** | Asunafo North | 0 | 0 | 0 | 0 | 0 | 0 | 0 | 0 | 0 | 0 | 0 | 0 | 0 | 0 | 0 | 0 | 0 | 0 | 0 | 0 | 0 | 0 | 0 | 0 | 1 | 0 | 0 | **1** |
| | Asunafo South | 1 | 0 | 2 | 1 | 0 | 0 | 0 | 0 | 0 | 0 | 0 | 0 | 0 | 0 | 0 | 0 | 0 | 0 | 0 | 0 | 0 | 0 | 0 | 0 | 0 | 0 | 0 | **4** |
| | Asutifi | 0 | 0 | 4 | 0 | 2 | 0 | 0 | 0 | 0 | 2 | 0 | 0 | 0 | 0 | 0 | 0 | 0 | 0 | [illegible] | 0 | 0 | 0 | 0 | 0 | 1 | 0 | 0 | **9** |
| | Berekum | 0 | 0 | 0 | 0 | 0 | 0 | 0 | 0 | 0 | 0 | 0 | 1 | 0 | 0 | 0 | 0 | 0 | 0 | [illegible] | 0 | 0 | 0 | 0 | 0 | 6 | 0 | 0 | **7** |
| | Dormaa | 0 | 0 | 0 | 0 | 0 | 0 | 0 | 0 | 0 | 1 | 1 | 0 | 0 | 0 | 0 | 0 | 2 | 0 | 0 | 3 | 0 | 0 | 1 | 0 | 12 | 1 | 0 | **21** |
| | Jaman North | 0 | 0 | 0 | 0 | 0 | 0 | 0 | 0 | 0 | 0 | 0 | 0 | 0 | 0 | [illegible] | 0 | 1 | 0 | 0 | 0 | 0 | 0 | 0 | 0 | 3 | 0 | 0 | **4** |
| | Jaman South | 0 | 0 | 0 | 0 | 0 | 0 | 0 | 0 | 0 | 0 | 0 | 0 | 0 | [illegible] | 0 | 0 | 0 | 0 | 1 | 1 | 0 | 0 | 3 | 0 | 10 | 0 | 0 | **15** |
| | Kintampo North | 0 | 0 | 0 | 0 | 0 | 0 | 0 | 0 | 0 | 1 | 0 | 0 | [illegible] | 0 | 1 | 0 | 0 | 0 | 0 | 0 | 0 | 0 | 0 | 0 | 1 | 0 | 0 | **3** |
| | Kintampo South | 0 | 0 | 1 | 0 | 2 | 0 | 0 | 0 | 0 | 0 | 0 | 0 | 0 | 0 | 0 | 0 | 1 | 0 | 0 | 0 | 1 | 0 | 0 | 0 | 0 | 0 | 0 | **5** |
| | Nkoranza | 0 | 0 | 1 | 0 | 6 | 0 | 0 | 0 | 0 | 0 | 0 | 3 | 0 | 0 | 0 | 0 | 0 | 0 | 0 | 0 | 0 | 0 | 0 | 0 | 0 | 0 | 0 | **10** |
| | Pru | 0 | 0 | 0 | 0 | 1 | 0 | 0 | 1 | 0 | 3 | 0 | 0 | 0 | 0 | 0 | 0 | 0 | 0 | 0 | 0 | 0 | 0 | 0 | 0 | 0 | 0 | 0 | **5** |
| | Sene | 0 | 0 | 0 | 0 | 0 | 0 | 0 | 0 | 0 | 1 | 0 | 1 | 0 | 0 | 0 | 0 | 0 | 0 | 0 | 0 | 0 | 0 | 0 | 0 | 0 | 0 | 0 | **2** |
| | Sunyani | 0 | 0 | 3 | 0 | 0 | 0 | 1 | 1 | 0 | 2 | 0 | 0 | 0 | 2 | 0 | 0 | 1 | 0 | 0 | 0 | 0 | 0 | 0 | 1 | 10 | 0 | 0 | **21** |
| | Tain | 0 | 0 | 6 | 0 | 3 | 0 | 0 | 0 | 0 | 0 | 0 | 0 | 0 | 1 | 0 | 0 | 1 | 0 | 0 | 1 | 0 | 0 | 0 | 0 | 15 | 2 | 0 | **29** |
| | Tano North | 0 | 0 | 3 | 0 | 0 | 0 | 0 | 0 | 0 | 0 | 0 | 1 | 0 | 0 | 0 | 0 | 0 | 0 | 0 | 0 | 0 | 0 | 0 | 0 | 1 | 0 | 0 | **5** |
| | Tano South | 0 | 0 | 11 | 0 | 2 | 0 | 0 | 0 | 0 | 1 | 0 | 1 | 0 | 0 | 0 | 0 | 0 | 0 | 0 | 1 | 0 | 0 | 0 | 0 | 0 | 0 | 0 | **16** |
| | Techiman | 0 | 0 | 3 | 0 | 1 | 0 | 0 | 1 | 0 | 0 | 0 | 0 | 0 | 0 | 0 | 0 | 0 | 0 | 0 | 0 | 0 | 0 | 0 | 0 | 1 | 0 | 0 | **6** |
| | **Brong Ahafo Total** | 1 | 0 | 34 | 1 | 17 | 0 | 1 | 3 | 0 | 11 | 1 | 7 | 0 | 3 | 1 | 0 | 6 | 0 | 1 | 6 | 1 | 0 | 4 | 1 | 61 | 3 | 0 | **163** |

C-UENR

*Daily Fire Detection count > 50 are highlighted in orange and Fire detection >=100 are highlighted in red.*

| Mar-2016 | | 01/03/16 | 02/03/16 | 03/03/16 | 04/03/16 | 05/03/16 | 06/03/16 | 07/03/16 | 08/03/16 | 09/03/16 | 10/03/16 | 11/03/16 | 12/03/16 | 13/03/16 | 15/03/16 | 16/03/16 | 17/03/16 | 19/03/16 | 20/03/16 | 21/03/16 | 22/03/16 | 23/03/16 | 24/03/16 | 26/03/16 | 27/03/16 | 28/03/16 | 29/03/16 | 31/03/16 | Monthly Total |
|---|---|---|---|---|---|---|---|---|---|---|---|---|---|---|---|---|---|---|---|---|---|---|---|---|---|---|---|---|---|
| **Central** | Agona | 0 | 0 | 2 | 0 | 0 | 0 | 0 | 0 | 0 | 0 | 0 | 0 | 0 | 0 | 0 | 0 | 0 | 0 | 0 | 0 | 0 | 0 | 0 | 0 | 0 | 0 | 0 | **2** |
| | Ajumako-Enyan-Esiam | 0 | 0 | 0 | 0 | 0 | 0 | 0 | 0 | 0 | 0 | 0 | 2 | 0 | 0 | 0 | 0 | 0 | 0 | 0 | 0 | 0 | 0 | 0 | 0 | 1 | 0 | 0 | **3** |
| | Asikuma Odoben | 0 | 0 | 0 | 0 | 1 | 0 | 0 | 0 | 0 | 0 | 0 | 0 | 0 | 0 | 0 | 0 | 0 | 0 | 0 | 0 | 0 | 0 | 0 | 0 | 0 | 0 | 0 | **1** |
| | Assin North | 0 | 0 | 6 | 0 | 2 | 0 | 0 | 2 | 0 | 4 | 0 | 0 | 0 | 0 | 0 | 0 | 0 | 0 | 1 | 0 | 0 | 0 | 0 | 0 | 0 | 0 | 0 | **15** |
| | Assin South | 1 | 0 | 11 | 0 | 1 | 0 | 0 | 0 | 0 | 1 | 0 | 0 | 0 | 0 | 0 | 0 | 0 | 0 | [illegible] | 0 | 0 | 0 | 0 | 0 | 0 | 0 | 0 | **16** |
| | Cape Coast | 0 | 0 | 0 | 0 | 0 | 0 | 0 | 0 | 0 | 2 | 0 | 0 | 0 | 0 | 0 | 0 | 0 | 0 | [illegible] | 0 | 0 | 0 | 0 | 0 | 0 | 0 | 0 | **2** |
| | Gomoa | 1 | 0 | 1 | 0 | 0 | 0 | 0 | 0 | 0 | 5 | 0 | 0 | 0 | 0 | 0 | 0 | 1 | 0 | 2 | 0 | 0 | 0 | 3 | 0 | 0 | 0 | 0 | **13** |
| | Komenda-Edina-Eguafo- | 0 | 0 | 1 | 0 | 0 | 0 | 0 | 0 | 0 | 1 | 0 | 0 | 0 | 0 | [illegible] | 0 | 0 | 0 | 2 | 0 | 0 | 0 | 0 | 0 | 0 | 0 | 0 | **4** |
| | Lower Denkyira | 1 | 0 | 6 | 0 | 0 | 0 | 0 | 0 | 0 | 3 | 0 | 0 | 0 | [illegible] | 0 | 0 | 0 | 0 | 0 | 0 | 0 | 0 | 0 | 0 | 0 | 0 | 0 | **10** |
| | Mfantsiman | 0 | 0 | 3 | 0 | 0 | 0 | 0 | 0 | 0 | 0 | 0 | 0 | [illegible] | 0 | 1 | 0 | 0 | 0 | 0 | 0 | 0 | 0 | 1 | 0 | 0 | 0 | 0 | **5** |
| | Upper Denkyira | 0 | 0 | 5 | 0 | 0 | 0 | 1 | 0 | 0 | 3 | 0 | 0 | 0 | 0 | 0 | 0 | 0 | 0 | 0 | 0 | 2 | 0 | 0 | 0 | 0 | 0 | 0 | **11** |
| | **Central Total** | 3 | 0 | 35 | 0 | 4 | 0 | 1 | 2 | 0 | 19 | 0 | 2 | 0 | 0 | 1 | 0 | 1 | 0 | 7 | 0 | 2 | 0 | 4 | 0 | 1 | 0 | 0 | **82** |
| **Eastern** | Afram Plains | 1 | 0 | 2 | 0 | 1 | 0 | 0 | 1 | 0 | 1 | 0 | 0 | 1 | 0 | 0 | 0 | 0 | 0 | 0 | 0 | 1 | 0 | 0 | 0 | 0 | 0 | 0 | **8** |
| | Akwapim North | 0 | 0 | 2 | 2 | 2 | 0 | 1 | 0 | 0 | 1 | 0 | 1 | 0 | 0 | 0 | 0 | 0 | 0 | 0 | 0 | 0 | 0 | 0 | 0 | 0 | 0 | 0 | **9** |
| | Akwapim South | 0 | 0 | 3 | 0 | 0 | 0 | 1 | 0 | 0 | 1 | 0 | 0 | 0 | 0 | 0 | 0 | 0 | 0 | 0 | 0 | 0 | 0 | 0 | 0 | 0 | 0 | 0 | **5** |
| | Asuogyaman | 0 | 0 | 0 | 0 | 0 | 0 | 0 | 0 | 0 | 0 | 0 | 0 | 0 | 0 | 0 | 0 | 0 | 0 | 0 | 0 | 1 | 0 | 0 | 0 | 0 | 0 | 0 | **1** |
| | Atiwa | 1 | 0 | 7 | 0 | 0 | 0 | 3 | 1 | 0 | 2 | 0 | 7 | 0 | 0 | 0 | 0 | 0 | 0 | 0 | 0 | 0 | 0 | 0 | 0 | 0 | 0 | 0 | **21** |
| | Birim North | 0 | 0 | 9 | 0 | 1 | 0 | 1 | 1 | 1 | 0 | 0 | 0 | 0 | 0 | 0 | 0 | 0 | 0 | 0 | 0 | 0 | 0 | 0 | 0 | 0 | 0 | 0 | **13** |
| | Birim South | 0 | 0 | 3 | 0 | 2 | 0 | 0 | 1 | 0 | 0 | 0 | 1 | 0 | 0 | 0 | 0 | 0 | 0 | 0 | 0 | 0 | 0 | 0 | 0 | 0 | 0 | 0 | **7** |
| | East Akim | 4 | 0 | 1 | 0 | 1 | 0 | 0 | 0 | 0 | 0 | 0 | 2 | 0 | 0 | 0 | 0 | 0 | 0 | 0 | 0 | 0 | 0 | 0 | 0 | 0 | 0 | 0 | **8** |

C - UENR

*Daily Fire Detection count > 50 are highlighted in orange and Fire detection >=100 are highlighted in red.*

| Mar-2016 | | 01/03/16 | 02/03/16 | 03/03/16 | 04/03/16 | 05/03/16 | 06/03/16 | 07/03/16 | 08/03/16 | 09/03/16 | 10/03/16 | 11/03/16 | 12/03/16 | 13/03/16 | 15/03/16 | 16/03/16 | 17/03/16 | 19/03/16 | 20/03/16 | 21/03/16 | 22/03/16 | 23/03/16 | 24/03/16 | 26/03/16 | 27/03/16 | 8/03/16 | 29/03/16 | 31/03/16 | Monthly Total |
|---|---|---|---|---|---|---|---|---|---|---|---|---|---|---|---|---|---|---|---|---|---|---|---|---|---|---|---|---|---|
| **Eastern** | Fanteakwa | 0 | 0 | 3 | 0 | 0 | 0 | 0 | 0 | 0 | 1 | 0 | 1 | 0 | 0 | 0 | 0 | 0 | 0 | 1 | 0 | 0 | 0 | 0 | 0 | 1 | 0 | 0 | **7** |
| | Kwabibirem | 0 | 0 | 2 | 0 | 0 | 0 | 1 | 0 | 0 | 0 | 0 | 0 | 0 | 0 | 0 | 0 | 0 | 0 | 0 | 0 | 0 | 0 | 0 | 0 | 0 | 0 | 0 | **3** |
| | Kwahu South | 0 | 0 | 0 | 0 | 0 | 0 | 0 | 0 | 0 | 2 | 0 | 0 | 0 | 0 | 0 | 0 | 0 | 0 | 0 | 0 | [illegible] | 0 | [illegible] | 0 | 2 | 0 | 0 | **4** |
| | Kwahu West | 0 | 0 | 1 | 0 | 0 | 0 | 0 | 0 | 0 | 0 | 0 | 0 | 0 | 0 | 0 | 0 | 0 | 0 | 0 | 0 | 0 | 0 | 0 | 0 | 0 | 0 | 0 | **1** |
| | Manya Krobo | 0 | 0 | 3 | 0 | 0 | 0 | 0 | 0 | 0 | 1 | 0 | 0 | 0 | 0 | 0 | 0 | 0 | 0 | [illegible] | 0 | 0 | 0 | 0 | 0 | 0 | 0 | 0 | **4** |
| | New Juaben | 0 | 0 | 0 | 0 | 0 | 0 | 0 | 0 | 0 | 0 | 0 | 1 | 0 | 0 | 0 | 0 | 0 | 0 | [illegible] | 0 | 0 | 0 | 0 | 0 | 0 | 0 | 0 | **1** |
| | Suhum Kraboa | 0 | 0 | 1 | 0 | 0 | 0 | 0 | 0 | 0 | 0 | 0 | 0 | 0 | 1 | 0 | 0 | 0 | 0 | 0 | 0 | 0 | 0 | 0 | 0 | 0 | 0 | 0 | **2** |
| | Yilo Krobo | 0 | 0 | 1 | 0 | 0 | 0 | 1 | 0 | 0 | 4 | 0 | 4 | 0 | 0 | [illegible] | 0 | 0 | 0 | 0 | 0 | 0 | 0 | 0 | 0 | 0 | 0 | 0 | **10** |
| | **Eastern Total** | 6 | 0 | 38 | 2 | 7 | 0 | 8 | 4 | 1 | 13 | 0 | 17 | 1 | [illegible] | 0 | 0 | 0 | 0 | 1 | 0 | 2 | 0 | 0 | 0 | 3 | 0 | 0 | **104** |
| **Greater Accra** | Dangbe East | 0 | 0 | 1 | 0 | 0 | 0 | 0 | 0 | 0 | 0 | 0 | 0 | [illegible] | 0 | 0 | 0 | 0 | 0 | 0 | 0 | 0 | 0 | 0 | 0 | 0 | 0 | 0 | **1** |
| | Tema | 0 | 0 | 0 | 0 | 0 | 0 | 0 | 0 | 0 | 1 | 0 | 0 | 0 | 0 | 0 | 0 | 0 | 0 | 0 | 0 | 0 | 0 | 0 | 0 | 0 | 0 | 0 | **1** |
| | **Greater Accra Total** | 0 | 0 | 1 | 0 | 0 | 0 | 0 | 0 | 0 | 1 | 0 | 0 | 0 | 0 | 0 | 0 | 0 | 0 | 0 | 0 | 0 | 0 | 0 | 0 | 0 | 0 | 0 | **2** |
| **Northern** | Bole | 0 | 0 | 1 | 0 | 1 | 0 | 0 | 0 | 0 | 0 | 0 | 0 | 0 | 0 | 0 | 0 | 0 | 0 | 0 | 0 | 0 | 0 | 0 | 0 | 0 | 0 | 0 | **2** |
| | Bunkpurugu Yunyoo | 0 | 0 | 0 | 0 | 1 | 0 | 1 | [illegible] | 0 | 1 | 0 | 0 | 0 | 0 | 0 | 0 | 0 | 0 | 0 | 0 | 0 | 0 | 0 | 0 | 0 | 0 | 0 | **3** |
| | Central Gonja | 0 | 0 | 3 | 2 | 5 | [illegible] | 1 | 0 | 0 | 0 | 0 | 0 | 0 | 1 | 0 | 0 | 0 | 0 | 0 | 0 | 0 | 0 | 0 | 0 | 0 | 0 | 0 | **12** |
| | East Gonja | 0 | 0 | 3 | 0 | 3 | 4 | 0 | 0 | 0 | 4 | 0 | 0 | 2 | 0 | 0 | 0 | 0 | 0 | 2 | 0 | 0 | 0 | 0 | 0 | 0 | 0 | 0 | **18** |
| | East Mamprusi | 0 | 0 | 2 | [illegible] | [illegible] | 0 | 0 | 0 | 0 | 3 | 0 | 0 | 0 | 0 | 0 | 0 | 0 | 0 | 0 | 0 | 0 | 0 | 0 | 0 | 0 | 0 | 0 | **6** |
| | Gushiegu | 0 | 3 | 2 | 0 | 0 | 0 | 0 | 0 | 3 | 5 | 0 | 0 | 0 | 0 | 0 | 0 | 2 | 0 | 0 | 0 | 0 | 0 | 1 | 0 | 0 | 0 | 0 | **16** |
| | Nanumba North | 0 | 0 | 6 | 0 | 3 | 0 | 0 | 0 | 1 | 1 | 0 | 0 | 0 | 0 | 0 | 0 | 0 | 0 | 0 | 0 | 0 | 0 | 0 | 0 | 1 | 0 | 0 | **12** |
| | Nanumba South | 0 | 0 | 0 | 1 | 1 | 2 | 0 | 0 | 0 | 0 | 0 | 0 | 0 | 0 | 0 | 0 | 0 | 0 | 0 | 0 | 0 | 0 | 0 | 0 | 0 | 0 | 0 | **4** |

EORIC - UENR COPY

*Daily Fire Detection count > 50 are highlighted in orange and Fire detection >=100 are highlighted in red.*

| Mar-2016 | | 01/03/16 | 02/03/16 | 03/03/16 | 04/03/16 | 05/03/16 | 06/03/16 | 07/03/16 | 08/03/16 | 09/03/16 | 10/03/16 | 11/03/16 | 12/03/16 | 13/03/16 | 15/03/16 | 16/03/16 | 17/03/16 | 19/03/16 | 20/03/16 | 21/03/16 | 22/03/16 | 23/03/16 | 24/03/16 | 26/03/16 | 27/03/16 | 8/03/16 | 29/03/16 | 31/03/16 | Monthly Total |
|---|---|---|---|---|---|---|---|---|---|---|---|---|---|---|---|---|---|---|---|---|---|---|---|---|---|---|---|---|---|
| **Northern** | Saboba Chereponi | 0 | 0 | 5 | 0 | 18 | 0 | 0 | 0 | 4 | 0 | 0 | 0 | 0 | 0 | 0 | 0 | 4 | 3 | 0 | 0 | 0 | 1 | 2 | 0 | 2 | 0 | 0 | **39** |
| | Savelugu Nanton | 0 | 0 | 1 | 0 | 0 | 0 | 0 | 0 | 0 | 1 | 0 | 0 | 0 | 0 | 0 | 0 | 0 | 0 | 0 | 0 | 0 | 0 | 0 | 0 | 0 | 0 | 0 | **2** |
| | Sawa-Tuna-Kalba | 0 | 0 | 11 | 0 | 4 | 0 | 0 | 0 | 0 | 0 | 0 | 0 | 0 | 0 | 0 | 0 | 0 | 0 | 0 | 0 | | 0 | | 0 | 0 | 0 | 0 | **15** |
| | Tolon-Kumbungu | 0 | 0 | 0 | 0 | 3 | 0 | 1 | 1 | 0 | 0 | 0 | 0 | 0 | 0 | 0 | 0 | 0 | 0 | 0 | 0 | 0 | 0 | 0 | 0 | 1 | 0 | 0 | **6** |
| | West Gonja | 1 | 0 | 7 | 0 | 5 | 1 | 0 | 6 | 0 | 1 | 0 | 1 | 0 | 1 | 0 | 0 | 0 | 0 | | 0 | 0 | 0 | 0 | 0 | 0 | 0 | 0 | **23** |
| | West Mamprusi | 0 | 1 | 4 | 1 | 1 | 0 | 0 | 0 | 0 | 4 | 0 | 0 | 1 | 0 | 0 | 0 | 0 | 0 | | 0 | 0 | 0 | 0 | 0 | 0 | 0 | 0 | **12** |
| | Yendi | 2 | 0 | 7 | 0 | 2 | 0 | 0 | 0 | 0 | 0 | 0 | 0 | 0 | 2 | 0 | 0 | 0 | 0 | 0 | 0 | 0 | 0 | 0 | 0 | 1 | 0 | 0 | **14** |
| | Zabzugu Tatale | 0 | 0 | 1 | 0 | 0 | 0 | 3 | 0 | 0 | 1 | 0 | 0 | 0 | 1 | | 0 | 2 | 0 | 2 | 0 | 0 | 0 | 0 | 0 | 0 | 1 | 0 | **11** |
| | **Northern Total** | 3 | 4 | 53 | 4 | 48 | 7 | 6 | 7 | 8 | 21 | 0 | 1 | 3 | 5 | 0 | 0 | 8 | 3 | 4 | 0 | 0 | 1 | 3 | 0 | 5 | 1 | 0 | **195** |
| **Upper East** | Bawku West | 0 | 0 | 0 | 0 | 0 | 0 | 0 | 0 | 0 | 2 | 0 | 0 | | 0 | 0 | 0 | 0 | 0 | 0 | 0 | 0 | 0 | 0 | 0 | 0 | 0 | 0 | **2** |
| | Builsa | 1 | 0 | 0 | 0 | 0 | 0 | 0 | 0 | 0 | 4 | 0 | 0 | 0 | 0 | 0 | 0 | 0 | 0 | 0 | 0 | 0 | 0 | 0 | 0 | 0 | 0 | 2 | **7** |
| | Kassena Nankana | 0 | 0 | 1 | 0 | 0 | 0 | 0 | 0 | 0 | 0 | 0 | 0 | 0 | 0 | 0 | 0 | 0 | 0 | 0 | 0 | 0 | 0 | 0 | 0 | 0 | 0 | 0 | **1** |
| | Talensi Nabdam | 0 | 0 | 0 | 0 | 1 | 0 | 0 | 0 | 0 | 0 | 0 | 0 | 0 | 0 | 0 | 0 | 0 | 0 | 0 | 0 | 0 | 0 | 0 | 0 | 0 | 0 | 0 | **1** |
| | **Upper East Total** | 1 | 0 | 1 | 0 | 1 | 0 | 0 | 0 | 0 | 6 | 0 | 0 | 0 | 0 | 0 | 0 | 0 | 0 | 0 | 0 | 0 | 0 | 0 | 0 | 0 | 0 | 2 | **11** |
| **Upper West** | Lawra | 0 | 0 | 1 | 0 | 0 | | 0 | 0 | 0 | 0 | 0 | 0 | 0 | 0 | 0 | 0 | 0 | 0 | 0 | 0 | 0 | 0 | 0 | 0 | 0 | 0 | 0 | **1** |
| | Sissala East | 0 | 0 | 1 | 0 | 1 | 0 | 0 | 0 | 0 | 0 | 0 | 0 | 0 | 0 | 0 | 0 | 1 | 0 | 0 | 0 | 0 | 0 | 0 | 0 | 0 | 0 | 0 | **3** |
| | Sissala West | 0 | 0 | 0 | | | 0 | 0 | 0 | 0 | 1 | 0 | 0 | 0 | 0 | 0 | 0 | 1 | 0 | 0 | 0 | 0 | 0 | 0 | 0 | 0 | 0 | 0 | **2** |
| | Wa East | 1 | 0 | 2 | 0 | 0 | 0 | 0 | 0 | 0 | 0 | 0 | 0 | 1 | 0 | 0 | 0 | 0 | 0 | 0 | 0 | 0 | 0 | 0 | 0 | 0 | 0 | 0 | **4** |
| | **Upper West Total** | 1 | 0 | 4 | 0 | 1 | 0 | 0 | 0 | 0 | 1 | 0 | 0 | 1 | 0 | 0 | 0 | 2 | 0 | 0 | 0 | 0 | 0 | 0 | 0 | 0 | 0 | 0 | **10** |

EORIC - UENR COPY

*Daily Fire Detection count > 50 are highlighted in orange and Fire detection >=100 are highlighted in red.*

| Mar-2016 | | 01/03/16 | 02/03/16 | 03/03/16 | 04/03/16 | 05/03/16 | 06/03/16 | 07/03/16 | 08/03/16 | 09/03/16 | 10/03/16 | 11/03/16 | 12/03/16 | 13/03/16 | 15/03/16 | 16/03/16 | 17/03/16 | 19/03/16 | 20/03/16 | 21/03/16 | 22/03/16 | 23/03/16 | 24/03/16 | 26/03/16 | 27/03/16 | 8/03/16 | 29/03/16 | 31/03/16 | Monthly Total |
|---|---|---|---|---|---|---|---|---|---|---|---|---|---|---|---|---|---|---|---|---|---|---|---|---|---|---|---|---|---|
| **Volta** | Adaklu Anyigbe | 0 | 0 | 1 | 0 | 1 | 0 | 0 | 0 | 0 | 3 | 0 | 0 | 0 | 0 | 0 | 0 | 0 | 0 | 0 | 0 | 0 | 0 | 0 | 0 | 2 | 0 | 0 | **7** |
| | Akatsi | 0 | 0 | 0 | 0 | 0 | 0 | 0 | 0 | 0 | 0 | 0 | 1 | 0 | 0 | 0 | 0 | 0 | 0 | 0 | 0 | 0 | 0 | 0 | 0 | 0 | 0 | 0 | **1** |
| | Ho | 4 | 0 | 2 | 0 | 0 | 0 | 3 | 0 | 0 | 0 | 0 | 0 | 0 | 0 | 0 | 1 | 3 | 0 | 0 | 0 | [illegible] | 0 | [illegible] | 0 | 2 | 0 | 0 | **15** |
| | Hohoe | 0 | 0 | 3 | 1 | 4 | 0 | 0 | 0 | 0 | 0 | 0 | 1 | 0 | 0 | 0 | 0 | 0 | 0 | 0 | 0 | 1 | 0 | 0 | 0 | 0 | 0 | 0 | **10** |
| | Jasikan | 1 | 0 | 6 | 0 | 4 | 0 | 1 | 0 | 1 | 1 | 0 | 2 | 0 | 0 | 0 | 0 | 0 | 0 | [illegible] | 0 | 3 | 0 | 0 | 0 | 0 | 0 | 0 | **19** |
| | Kadjebi | 0 | 0 | 1 | 0 | 0 | 0 | 0 | 0 | 0 | 1 | 0 | 0 | 0 | 0 | 1 | 0 | 0 | 0 | [illegible] | 0 | 2 | 0 | 0 | 0 | 0 | 0 | 0 | **5** |
| | Keta | 0 | 0 | 0 | 0 | 0 | 0 | 0 | 0 | 0 | 0 | 0 | 0 | 0 | 0 | 0 | 1 | 0 | 0 | 0 | 0 | 0 | 0 | 0 | 0 | 0 | 0 | 0 | **1** |
| | Kpandu | 0 | 0 | 0 | 0 | 0 | 0 | 0 | 0 | 0 | 1 | 0 | 0 | 0 | 0 | [illegible] | 1 | 0 | 0 | 0 | 0 | 0 | 0 | 0 | 0 | 1 | 0 | 0 | **3** |
| | Krachi | 0 | 0 | 0 | 0 | 0 | 0 | 0 | 0 | 0 | 0 | 0 | 1 | 0 | [illegible] | 0 | 0 | 0 | 0 | 0 | 0 | 0 | 0 | 0 | 0 | 0 | 0 | 0 | **1** |
| | Krachi East | 0 | 0 | 0 | 0 | 0 | 0 | 0 | 0 | 0 | 0 | 0 | 1 | [illegible] | 0 | 0 | 0 | 0 | 0 | 0 | 0 | 0 | 0 | 0 | 0 | 0 | 0 | 0 | **1** |
| | Nkwanta | 0 | 0 | 9 | 0 | 7 | 1 | 0 | 0 | 1 | 2 | 0 | 2 | 0 | 1 | 0 | 0 | 0 | 0 | 2 | 0 | 3 | 0 | 0 | 0 | 3 | 0 | 0 | **31** |
| | North Tongu | 1 | 0 | 0 | 0 | 1 | 0 | 0 | 0 | 0 | 0 | 0 | 0 | 0 | 0 | 0 | 0 | 0 | 0 | 0 | 0 | 0 | 0 | 0 | 0 | 0 | 0 | 0 | **2** |
| | South Dayi | 0 | 0 | 0 | 0 | 0 | 0 | 0 | 0 | 0 | 0 | 0 | 0 | 0 | 0 | 0 | 0 | 0 | 0 | 0 | 0 | 3 | 0 | 0 | 0 | 0 | 0 | 0 | **3** |
| | **Volta Total** | 6 | 0 | 22 | 1 | 17 | 1 | 4 | 0 | 2 | 8 | 0 | 8 | 0 | 1 | 1 | 3 | 3 | 0 | 2 | 0 | 12 | 0 | 0 | 0 | 8 | 0 | 0 | **99** |
| **Western** | Ahanta West | 0 | 0 | 2 | 0 | 0 | [illegible] | 0 | 0 | 0 | 0 | 0 | 0 | 0 | 0 | 0 | 0 | 0 | 0 | 0 | 0 | 0 | 0 | 0 | 0 | 0 | 0 | 0 | **2** |
| | Aowin-Suaman | 0 | 0 | [illegible] | 1 | 1 | 0 | 0 | 0 | 0 | 0 | 0 | 3 | 0 | 0 | 0 | 0 | 0 | 0 | 0 | 0 | 0 | 0 | 1 | 0 | 0 | 0 | 0 | **12** |
| | Bia | 0 | 0 | 2 | [illegible] | [illegible] | 0 | 0 | 0 | 0 | 0 | 0 | 0 | 0 | 0 | 0 | 0 | 0 | 0 | 0 | 0 | 0 | 0 | 0 | 0 | 0 | 0 | 0 | **5** |
| | Bibiani Anhwiaso | 1 | 0 | 3 | 0 | 0 | 0 | 0 | 1 | 0 | 1 | 0 | 0 | 0 | 0 | 0 | 0 | 0 | 0 | 0 | 0 | 0 | 0 | 0 | 0 | 0 | 0 | 0 | **6** |
| | Jomoro | 0 | 0 | 3 | 0 | 0 | 0 | 0 | 0 | 0 | 1 | 0 | 0 | 0 | 0 | 0 | 2 | 0 | 0 | 5 | 0 | 0 | 0 | 1 | 0 | 1 | 0 | 0 | **13** |
| | Juabeso | 1 | 0 | 2 | 0 | 2 | 0 | 0 | 0 | 0 | 2 | 0 | 0 | 0 | 0 | 0 | 0 | 0 | 0 | 0 | 0 | 0 | 0 | 1 | 0 | 0 | 0 | 0 | **8** |

EORIC - UENR copy

*Daily Fire Detection count > 50 are highlighted in orange and Fire detection >=100 are highlighted in red.*

| Mar-2016 | | 01/03/16 | 02/03/16 | 03/03/16 | 04/03/16 | 05/03/16 | 06/03/16 | 07/03/16 | 08/03/16 | 09/03/16 | 10/03/16 | 11/03/16 | 12/03/16 | 13/03/16 | 15/03/16 | 16/03/16 | 17/03/16 | 19/03/16 | 20/03/16 | 21/03/16 | 22/03/16 | 23/03/16 | 24/03/16 | 26/03/16 | 27/03/16 | 8/03/16 | 29/03/16 | 31/03/16 | Monthly Total |
|---|---|---|---|---|---|---|---|---|---|---|---|---|---|---|---|---|---|---|---|---|---|---|---|---|---|---|---|---|---|
| **Western** | Mpohor Wassa East | 0 | 0 | 4 | 0 | 0 | 0 | 4 | 0 | 0 | 2 | 2 | 0 | 0 | 0 | 0 | 0 | 0 | 0 | 0 | 0 | 1 | 0 | 0 | 0 | 0 | 0 | 0 | **13** |
| | Nzema East | 0 | 0 | 3 | 0 | 2 | 0 | 0 | 0 | 0 | 3 | 0 | 2 | 0 | 0 | 0 | 1 | 0 | 0 | 7 | 0 | 0 | 0 | 1 | 0 | 2 | 0 | 0 | **21** |
| | Sefwi Wiawso | 2 | 0 | 2 | 0 | 0 | 0 | 0 | 0 | 0 | 2 | 0 | 0 | 0 | 0 | 0 | 1 | 0 | 0 | 0 | 0 | | 0 | | 0 | 3 | 0 | 0 | **10** |
| | Shama Ahanta East | 0 | 0 | 1 | 0 | 1 | 0 | 0 | 0 | 0 | 0 | 0 | 0 | 0 | 0 | 0 | 0 | 0 | 0 | 5 | 0 | 0 | 0 | 0 | 0 | 1 | 0 | 0 | **8** |
| | Wasa Amenfi East | 0 | 0 | 7 | 0 | 0 | 0 | 0 | 0 | 0 | 1 | 0 | 0 | 0 | 0 | 0 | 0 | 0 | 0 | | 0 | 0 | 0 | 0 | 0 | 0 | 0 | 0 | **10** |
| | Wasa Amenfi West | 2 | 0 | 21 | 0 | 0 | 2 | 0 | 0 | 0 | 2 | 0 | 0 | 0 | 0 | 0 | 1 | 0 | 0 | | 0 | 0 | 0 | 2 | 0 | 0 | 0 | 0 | **31** |
| | Wassa West | 2 | 0 | 7 | 0 | 4 | 0 | 3 | 2 | 0 | 5 | 0 | 4 | 0 | 0 | 0 | 1 | 0 | 0 | 4 | 1 | 2 | 0 | 2 | 0 | 0 | 0 | 0 | **37** |
| | **Western Total** | 8 | 0 | 63 | 1 | 13 | 2 | 7 | 3 | 0 | 19 | 2 | 9 | 0 | 0 | 0 | 6 | 0 | 0 | 24 | 1 | 3 | 0 | 8 | 0 | 7 | 0 | 0 | **176** |
| **Provincial Total** | | **31** | **4** | **337** | **14** | **125** | **10** | **29** | **31** | **11** | **115** | **3** | **2** | **5** | **12** | **3** | **9** | **20** | **3** | **44** | **10** | **20** | **1** | **24** | **2** | **85** | **4** | **2** | **1006** |

EORIC - UENR COPY

*Daily Fire Detection count > 50 are highlighted in orange and Fire detection >=100 are highlighted in red.*

| Apr-2016 | | 02/04/16 | 04/04/16 | 06/04/16 | 07/04/16 | 08/04/16 | 11/04/16 | 13/04/16 | 14/04/16 | 16/04/16 | 20/04/16 | 21/04/16 | 22/04/16 | 23/04/16 | 24/04/16 | 25/04/16 | 26/04/16 | 27/04/16 | 29/04/16 | Monthly Total |
|---|---|---|---|---|---|---|---|---|---|---|---|---|---|---|---|---|---|---|---|---|
| **Ashanti** | Ahafo Ano North | 0 | 0 | 2 | 0 | 0 | 0 | 0 | 0 | 0 | 0 | 0 | 0 | 0 | 0 | 0 | 0 | 0 | 0 | **2** |
| | Ahafo Ano South | 0 | 0 | 0 | 0 | 0 | 0 | 0 | 0 | 0 | 0 | 0 | 0 | 0 | 0 | 1 | 0 | 0 | 1 | **2** |
| | Amansie Central | 0 | 0 | 0 | 0 | 1 | 0 | 0 | 0 | 0 | 0 | 0 | 0 | 0 | 0 | 0 | 0 | 0 | 0 | **1** |
| | Amansie West | 0 | 0 | 0 | 0 | 0 | 0 | 0 | 0 | 0 | 1 | 0 | 3 | 0 | 0 | 0 | 0 | 0 | 0 | **4** |
| | Atwima Mponua | 16 | 0 | 2 | 0 | 2 | 0 | 0 | 0 | 0 | 0 | 0 | 0 | 0 | 0 | 0 | 0 | 1 | | **21** |
| | Bosomtwe-Kwanwoma | 0 | 0 | 0 | 0 | 0 | 0 | 0 | 0 | 0 | 0 | 0 | 1 | 0 | 0 | 0 | 0 | 0 | 0 | **1** |
| | **Ashanti Total** | 16 | 0 | 4 | 0 | 3 | 0 | 0 | 0 | 0 | 1 | 0 | 4 | 0 | 0 | 1 | 0 | 1 | 1 | **31** |
| **Brong Ahafo** | Asunafo South | 0 | 2 | 0 | 0 | 0 | 0 | 0 | 0 | 0 | 0 | 0 | 0 | 0 | 0 | 0 | 0 | 0 | 0 | **2** |
| | Asutifi | 0 | 0 | 0 | 0 | 0 | 1 | 0 | 0 | 0 | 0 | 0 | 0 | 0 | 0 | 0 | 0 | 0 | 0 | **1** |
| | Berekum | 1 | 0 | 0 | 0 | 0 | 3 | 0 | 0 | 0 | 0 | 0 | 0 | 0 | | 0 | 0 | 0 | 2 | **6** |
| | Dormaa | 0 | 1 | 0 | 0 | 0 | 2 | 0 | 0 | 0 | 0 | 0 | 0 | 0 | 0 | 0 | 0 | 0 | 1 | **4** |
| | Jaman North | 0 | 1 | 0 | 0 | 0 | 1 | 0 | | 0 | 0 | 0 | 0 | 0 | 0 | 0 | 0 | 0 | 0 | **2** |
| | Jaman South | 1 | 0 | 1 | 0 | 0 | 7 | | 0 | 0 | | 0 | 0 | 0 | 0 | 0 | 0 | 0 | 0 | **9** |
| | Kintampo North | 0 | 0 | 0 | 0 | 1 | 16 | 0 | 0 | 0 | 0 | 0 | 0 | 0 | 0 | 0 | 0 | 0 | 0 | **17** |
| | Kintampo South | 0 | 0 | 0 | 0 | 0 | 0 | 0 | 0 | 0 | 1 | 0 | 0 | 0 | 0 | 1 | 0 | 0 | 0 | **2** |
| | Nkoranza | 0 | 0 | 0 | 0 | 0 | 0 | 0 | 0 | 0 | 1 | 0 | 0 | 0 | 0 | 0 | 0 | 0 | 0 | **1** |
| | Sene | 0 | 0 | 0 | 0 | 0 | 0 | 0 | 1 | 0 | 0 | 0 | 0 | 0 | 1 | 0 | 0 | 0 | 0 | **2** |
| | Sunyani | 0 | | 0 | 0 | 0 | 1 | 1 | 0 | 0 | 0 | 0 | 4 | 0 | 0 | 0 | 0 | 0 | 0 | **6** |
| | Tain | 0 | 0 | 2 | 0 | 0 | 1 | 0 | 0 | 0 | 0 | 0 | 0 | 0 | 0 | 0 | 0 | 0 | 0 | **3** |

EORIC - UENR copy

*Daily Fire Detection count > 50 are highlighted in orange and Fire detection >=100 are highlighted in red.*

Apr-2016

| Region | District | 02/04/16 | 04/04/16 | 06/04/16 | 07/04/16 | 08/04/16 | 11/04/16 | 13/04/16 | 14/04/16 | 16/04/16 | 20/04/16 | 21/04/16 | 22/04/16 | 23/04/16 | 24/04/16 | 25/04/16 | 26/04/16 | 27/04/16 | 29/04/16 | Monthly Total |
|---|---|---|---|---|---|---|---|---|---|---|---|---|---|---|---|---|---|---|---|---|
| **Brong Ahafo** | Tano North | 0 | 0 | 1 | 0 | 0 | 0 | 0 | 0 | 0 | 0 | 0 | 0 | 0 | 0 | 0 | 0 | 0 | 0 | **1** |
| | **Brong Ahafo Total** | 2 | 4 | 4 | 0 | 1 | 32 | 1 | 1 | 0 | 2 | 0 | 4 | 0 | 1 | 1 | 0 | 0 | 3 | **56** |
| **Central** | Abura-Asebu-Kwamankese | 0 | 0 | 0 | 0 | 0 | 0 | 0 | 0 | 0 | 0 | 0 | 1 | 0 | 0 | 0 | 0 | 0 | 0 | **1** |
| | Ajumako-Enyan-Esiam | 0 | 0 | 0 | 0 | 0 | 0 | 0 | 0 | 0 | 1 | 0 | 0 | 0 | 0 | 0 | 0 | 0 | 0 | **1** |
| | Assin North | 0 | 0 | 0 | 0 | 0 | 0 | 0 | 0 | 0 | 1 | 0 | 0 | 0 | 0 | 0 | 0 | 0 | 0 | **1** |
| | Assin South | 0 | 0 | 0 | 0 | 0 | 0 | 0 | 0 | 0 | 3 | 0 | 0 | 0 | 0 | 0 | 0 | 0 | 0 | **3** |
| | Awutu Efutu Senya | 0 | 0 | 0 | 0 | 0 | 0 | 0 | 0 | 0 | 0 | 0 | 0 | 0 | 0 | 0 | 1 | 0 | 0 | **1** |
| | Cape Coast | 0 | 0 | 0 | 0 | 0 | 0 | 0 | 0 | 0 | 1 | 0 | 0 | 0 | 0 | [illegible] | 0 | 0 | 0 | **1** |
| | Gomoa | 0 | 0 | 0 | 0 | 0 | 0 | 0 | 0 | 0 | 0 | 0 | 2 | 0 | [illegible] | 0 | 1 | 0 | 0 | **3** |
| | Komenda-Edina-Eguafo- | 0 | 0 | 0 | 0 | 0 | 0 | 0 | 0 | 0 | 0 | 0 | 3 | [illegible] | 0 | 0 | 0 | 0 | 0 | **3** |
| | Lower Denkyira | 0 | 0 | 0 | 0 | 0 | 0 | 1 | 0 | 0 | 2 | 0 | 1 | 0 | 0 | 0 | 0 | 0 | 0 | **4** |
| | Mfantsiman | 0 | 3 | 0 | 0 | 0 | 0 | 0 | 0 | 0 | 0 | 0 | 3 | 1 | 0 | 0 | 17 | 19 | 0 | **43** |
| | Upper Denkyira | 0 | 0 | 1 | 0 | 0 | 0 | 0 | 0 | 0 | 1 | 0 | 0 | 1 | 0 | 0 | 0 | 0 | 0 | **3** |
| | **Central Total** | 0 | 3 | 1 | 0 | 0 | 0 | 1 | 0 | 0 | 9 | 0 | 10 | 2 | 0 | 0 | 19 | 19 | 0 | **64** |
| **Eastern** | Afram Plains | 0 | 0 | 0 | 1 | 1 | [illegible] | 0 | 0 | 0 | 0 | 0 | 0 | 0 | 0 | 0 | 0 | 0 | 0 | **2** |
| | Akwapim North | 0 | 0 | 0 | 0 | 0 | 0 | 0 | 0 | 0 | 0 | 0 | 1 | 0 | 0 | 0 | 0 | 0 | 0 | **1** |
| | Atiwa | 0 | 0 | 0 | [illegible] | [illegible] | 0 | 0 | 0 | 0 | 0 | 0 | 1 | 0 | 0 | 0 | 0 | 0 | 0 | **1** |
| | Birim South | 0 | 0 | 0 | 0 | 0 | 0 | 0 | 0 | 0 | 1 | 0 | 0 | 0 | 0 | 0 | 0 | 0 | 0 | **1** |
| | Kwahu West | 0 | 0 | 0 | 0 | 0 | 0 | 0 | 0 | 0 | 1 | 0 | 0 | 0 | 0 | 0 | 0 | 0 | 0 | **1** |
| | **Eastern Total** | 0 | 0 | 0 | 1 | 1 | 0 | 0 | 0 | 0 | 2 | 0 | 2 | 0 | 0 | 0 | 0 | 0 | 0 | **6** |

EORIC - UENR copy

*Daily Fire Detection count > 50 are highlighted in orange and Fire detection >=100 are highlighted in red.*

| Apr-2016 | | 02/04/16 | 04/04/16 | 06/04/16 | 07/04/16 | 08/04/16 | 11/04/16 | 13/04/16 | 14/04/16 | 16/04/16 | 20/04/16 | 21/04/16 | 22/04/16 | 23/04/16 | 24/04/16 | 25/04/16 | 26/04/16 | 27/04/16 | 29/04/16 | Monthly Total |
|---|---|---|---|---|---|---|---|---|---|---|---|---|---|---|---|---|---|---|---|---|
| **Greater Accra** | Dangbe East | 0 | 0 | 0 | 0 | 0 | 0 | 1 | 0 | 0 | 1 | 0 | 0 | 0 | 0 | 0 | 0 | 0 | 0 | **2** |
| | Tema | 0 | 0 | 0 | 0 | 0 | 0 | 1 | 0 | 0 | 0 | 0 | 0 | 0 | 0 | 0 | 0 | 0 | 0 | **1** |
| | **Greater Accra Total** | 0 | 0 | 0 | 0 | 0 | 0 | 2 | 0 | 0 | 1 | 0 | 0 | 0 | 0 | 0 | 0 | 0 | 0 | **3** |
| **Northern** | East Gonja | 0 | 1 | 0 | 0 | 0 | 0 | 0 | 0 | 0 | 0 | 0 | 0 | 0 | 0 | 0 | 0 | 0 | 0 | **1** |
| | East Mamprusi | 0 | 0 | 0 | 0 | 0 | 0 | 0 | 0 | 0 | 1 | 0 | 0 | 0 | 0 | 0 | 0 | 0 | 0 | **1** |
| | Gushiegu | 0 | 1 | 0 | 0 | 0 | 0 | 0 | 0 | 0 | 0 | 0 | 0 | 0 | 0 | 0 | 0 | 0 | 0 | **1** |
| | West Mamprusi | 0 | 1 | 0 | 0 | 0 | 0 | 0 | 0 | 0 | 0 | 0 | 0 | 0 | 0 | 0 | 0 | 0 | 0 | **1** |
| | Zabzugu Tatale | 0 | 1 | 0 | 0 | 0 | 18 | 0 | 0 | 0 | 0 | 0 | 0 | 0 | 0 | [illegible] | 0 | 0 | 1 | **20** |
| | **Northern Total** | 0 | 4 | 0 | 0 | 0 | 18 | 0 | 0 | 0 | 1 | 0 | 0 | 0 | 0 | 0 | 0 | 0 | 1 | **24** |
| **Upper West** | Sissala East | 0 | 0 | 0 | 0 | 0 | 0 | 0 | 0 | 0 | 0 | 0 | 1 | [illegible] | 0 | 0 | 0 | 0 | 0 | **1** |
| | Wa East | 0 | 2 | 0 | 0 | 0 | 0 | 0 | 0 | 0 | 0 | 0 | 0 | 0 | 0 | 0 | 0 | 0 | 0 | **2** |
| | **Upper West Total** | 0 | 2 | 0 | 0 | 0 | 0 | 0 | 0 | 0 | 0 | 0 | 1 | 0 | 0 | 0 | 0 | 0 | 0 | **3** |
| **Volta** | Ho | 0 | 0 | 0 | 0 | 0 | 0 | 0 | 0 | 0 | 0 | 0 | 1 | 0 | 0 | 0 | 0 | 0 | 0 | **1** |
| | Hohoe | 0 | 0 | 0 | 0 | 0 | 0 | 1 | [illegible] | 0 | 0 | 0 | 0 | 0 | 0 | 0 | 0 | 0 | 0 | **1** |
| | Jasikan | 0 | 0 | 0 | 0 | 0 | [illegible] | 0 | 18 | 0 | 0 | 0 | 1 | 0 | 0 | 0 | 0 | 0 | 0 | **19** |
| | Keta | 0 | 20 | 0 | 0 | 0 | 0 | 0 | 0 | 0 | 0 | 0 | 0 | 0 | 0 | 0 | 0 | 0 | 0 | **20** |
| | South Dayi | 0 | 0 | 0 | [illegible] | [illegible] | 0 | 0 | 0 | 0 | 1 | 0 | 0 | 0 | 0 | 0 | 0 | 0 | 0 | **1** |
| | **Volta Total** | 0 | 0 | 0 | 0 | 0 | 0 | 1 | 18 | 0 | 1 | 0 | 2 | 0 | 0 | 0 | 0 | 0 | 0 | **42** |
| **Western** | Ahanta West | 0 | 0 | 18 | 0 | 0 | 3 | 0 | 0 | 0 | 0 | 0 | 3 | 0 | 0 | 0 | 0 | 0 | 0 | **24** |
| | Aowin-Suaman | 0 | 1 | 1 | 0 | 0 | 0 | 0 | 0 | 0 | 1 | 0 | 0 | 0 | 0 | 0 | 0 | 0 | 0 | **3** |

EORIC - UENR copy

*Daily Fire Detection count > 50 are highlighted in orange and Fire detection >=100 are highlighted in red.*

| Apr-2016 | | 02/04/16 | 04/04/16 | 06/04/16 | 07/04/16 | 08/04/16 | 11/04/16 | 13/04/16 | 14/04/16 | 16/04/16 | 20/04/16 | 21/04/16 | 22/04/16 | 23/04/16 | 24/04/16 | 25/04/16 | 26/04/16 | 27/04/16 | 29/04/16 | Monthly Total |
|---|---|---|---|---|---|---|---|---|---|---|---|---|---|---|---|---|---|---|---|---|
| **Western** | Bia | 0 | 1 | 0 | 0 | 0 | 0 | 0 | 0 | 0 | 1 | 0 | 0 | 0 | 0 | 0 | 0 | 0 | 0 | **2** |
| | Bibiani Anhwiaso | 0 | 1 | 0 | 0 | 0 | 0 | 0 | 0 | 0 | 0 | 0 | 0 | 0 | 0 | 0 | 0 | 0 | 0 | **1** |
| | Jomoro | 0 | 2 | 0 | 0 | 0 | 0 | 3 | 0 | 1 | 0 | 0 | 0 | 0 | 0 | 0 | 0 | 0 | 0 | **6** |
| | Mpohor Wassa East | 0 | 0 | 0 | 0 | 0 | 0 | 3 | 0 | 0 | 0 | 2 | 20 | 22 | 0 | 0 | 12 | 0 | 0 | **59** |
| | Nzema East | 0 | 5 | 5 | 0 | 0 | 5 | 5 | 0 | 0 | 1 | 0 | 3 | 1 | 0 | 0 | 0 | 0 | 0 | **25** |
| | Sefwi Wiawso | 0 | 3 | 0 | 0 | 0 | 0 | 0 | 0 | 0 | 1 | 0 | 0 | 0 | 0 | 1 | 0 | 0 | 0 | **5** |
| | Shama Ahanta East | 0 | 1 | 0 | 0 | 0 | 0 | 0 | 0 | 0 | 0 | 0 | 1 | 0 | 0 | 0 | 0 | 1 | 0 | **3** |
| | Wasa Amenfi East | 0 | 0 | 0 | 0 | 0 | 0 | 0 | 0 | 0 | 3 | 0 | 0 | 0 | 0 | [illegible] | 0 | 0 | 0 | **3** |
| | Wasa Amenfi West | 0 | 1 | 1 | 0 | 0 | 0 | 1 | 0 | 0 | 1 | 0 | 0 | 0 | [illegible] | 0 | 0 | 0 | 0 | **4** |
| | Wassa West | 0 | 1 | 1 | 0 | 0 | 1 | 1 | 0 | 0 | 1 | 0 | 2 | [illegible] | 0 | 0 | 0 | 0 | 0 | **7** |
| | **Western Total** | 0 | 16 | 26 | 0 | 0 | 9 | 13 | 0 | 1 | 9 | 2 | 29 | 23 | 0 | 1 | 12 | 1 | 0 | **142** |
| **Provincial Total** | | **18** | **49** | **35** | **1** | **5** | **59** | **18** | **19** | **1** | **26** | **2** | **52** | **25** | **1** | **3** | **31** | **21** | **5** | **371** |

EORIC - UENR copy

*Daily Fire Detection count > 50 are highlighted in orange and Fire detection >=100 are highlighted in red.*

| May-2016 | | 01/05/16 | 02/05/16 | 05/05/16 | 06/05/16 | 08/05/16 | 10/05/16 | 11/05/16 | 15/05/16 | Monthly Total |
|---|---|---|---|---|---|---|---|---|---|---|
| **Ashanti** | Adansi North | 0 | 0 | 0 | 0 | 0 | 19 | 0 | 0 | **19** |
| | Atwima Mponua | 0 | 0 | 0 | 0 | 0 | 0 | 0 | 1 | **1** |
| | **Ashanti Total** | 0 | 0 | 0 | 0 | 0 | 19 | 0 | 1 | **20** |
| **Brong Ahafo** | Jaman South | 0 | 0 | 0 | 0 | 0 | 0 | 0 | 1 | **1** |
| | Sene | 0 | 0 | 0 | 20 | 0 | 0 | 0 | 0 | **20** |
| | **Brong Ahafo Total** | 0 | 0 | 0 | 20 | 0 | 0 | 0 | 1 | **21** |
| **Central** | Assin North | 0 | 0 | 0 | 1 | 0 | 0 | 0 | 0 | **1** |
| | Awutu Efutu Senya | 1 | 0 | 0 | 0 | 0 | 0 | 0 | 0 | **1** |
| | Gomoa | 1 | 0 | 0 | 1 | 0 | 0 | 0 | 0 | **2** |
| | Komenda-Edina-Eguafo- | 0 | 0 | 0 | 0 | 0 | 2 | 0 | 0 | **2** |
| | Mfantsiman | 1 | 0 | 0 | 0 | 1 | 0 | 0 | 1 | **3** |
| | **Central Total** | 3 | 0 | 0 | 2 | 1 | 2 | 0 | | **9** |
| **Eastern** | East Akim | 0 | 0 | 0 | 1 | 0 | 0 | | 0 | **1** |
| | **Eastern Total** | 0 | 0 | 0 | 1 | 0 | 0 | 0 | 0 | **1** |
| **Greater Accra** | Tema | 0 | 18 | 0 | 0 | 0 | 0 | 0 | 0 | **18** |
| | **Greater Accra Total** | 0 | 18 | 0 | 0 | 0 | 0 | 0 | 0 | **18** |
| **Volta** | Ho | 1 | 0 | 0 | 0 | 0 | 0 | 0 | 0 | **1** |
| | Kpandu | 0 | | 0 | 1 | 0 | 0 | 0 | 0 | **1** |

EORIC - UENR copy

*Daily Fire Detection count > 50 are highlighted in orange and Fire detection >=100 are highlighted in red.*

| May-2016 | | 01/05/16 | 02/05/16 | 05/05/16 | 06/05/16 | 08/05/16 | 10/05/16 | 11/05/16 | 15/05/16 | Monthly Total |
|---|---|---|---|---|---|---|---|---|---|---|
| **Volta** | North Tongu | 0 | 0 | 1 | 1 | 0 | 0 | 0 | 0 | **2** |
| | **Volta Total** | 1 | 0 | 1 | 2 | 0 | 0 | 0 | 0 | **4** |
| **Western** | Jomoro | 0 | 0 | 0 | 0 | 0 | 0 | 20 | 0 | **20** |
| | Mpohor Wassa East | 0 | 0 | 0 | 0 | 0 | 3 | 0 | 0 | **3** |
| | Wasa Amenfi West | 1 | 0 | 0 | 0 | 0 | 0 | 0 | 0 | **1** |
| | **Western Total** | 1 | 0 | 0 | 0 | 0 | 3 | 20 | 0 | **24** |
| **Provincial Total** | | **5** | **18** | **1** | **25** | **1** | **24** | **20** | **3** | **97** |

EORIC - UENR copy

*Daily Fire Detection count > 50 are highlighted in orange and Fire detection >=100 are highlighted in red.*

| Jun-2016 | | 02/06/16 | 04/06/16 | 05/06/16 | Monthly Total |
|---|---|---|---|---|---|
| **Greater Accra** | Tema | 0 | 2 | 0 | **2** |
| | **Greater Accra Total** | 0 | 2 | 0 | **2** |
| **Volta** | Nkwanta | 1 | 0 | 0 | **1** |
| | South Tongu | 0 | 0 | 18 | **18** |
| | **Volta Total** | 1 | 0 | 18 | **19** |
| **Provincial Total** | | **1** | **2** | **18** | **21** |

EORIC - UENR copy

*Daily Fire Detection count > 50 are highlighted in orange and Fire detection >=100 are highlighted in red.*

| Jul-2016 | | 05/07/16 | 12/07/16 | 16/07/16 | 18/07/16 | Monthly Total |
|---|---|---|---|---|---|---|
| **Ashanti** | Sekyere East | 1 | 0 | 1 | 0 | **2** |
| | **Ashanti Total** | 1 | 0 | 1 | 0 | **2** |
| **Brong Ahafo** | Pru | 0 | 0 | 20 | 0 | **20** |
| | **Brong Ahafo Total** | 0 | 0 | 20 | 0 | **20** |
| **Central** | Mfantsiman | 0 | 0 | 0 | 1 | **1** |
| | **Central Total** | 0 | 0 | 0 | 1 | **1** |
| **Western** | Nzema East | 0 | 15 | 0 | 0 | **15** |
| | **Western Total** | 0 | 15 | 0 | 0 | **15** |
| **Provincial Total** | | **1** | **15** | **21** | **1** | **38** |

EORIC - UENR copy

*Daily Fire Detection count > 50 are highlighted in orange and Fire detection >=100 are highlighted in red.*

| Aug-2016 | | 05/08/16 | 10/08/16 | 12/08/16 | Monthly Total |
|---|---|---|---|---|---|
| **Ashanti** | Ejura Sekyedumas | 0 | 1 | 0 | **1** |
| | **Ashanti Total** | 0 | 1 | 0 | **1** |
| **Brong Ahafo** | Techiman | 1 | 0 | 0 | **1** |
| | **Brong Ahafo Total** | 1 | 0 | 0 | **1** |
| **Eastern** | Afram Plains | 0 | 1 | 0 | **1** |
| | Manya Krobo | 1 | 0 | 0 | **1** |
| | **Eastern Total** | 1 | 1 | 0 | **2** |
| **Volta** | Adaklu Anyigbe | 0 | 0 | 1 | **1** |
| | **Volta Total** | 0 | 0 | 1 | **1** |
| **Provincial Total** | | **2** | **2** | **1** | **5** |

EORIC - UENR copy

*Daily Fire Detection count > 50 are highlighted in orange and Fire detection >=100 are highlighted in red.*

| Aug-2016 | | 05/08/16 | 10/08/16 | 12/08/16 | 29/08/16 | Monthly Total |
|---|---|---|---|---|---|---|
| **Ashanti** | Ejura Sekyedumas | 0 | 1 | 0 | 1 | **2** |
| | **Ashanti Total** | 0 | 1 | 0 | 1 | **2** |
| **Brong Ahafo** | Techiman | 1 | 0 | 0 | 0 | **1** |
| | **Brong Ahafo Total** | 1 | 0 | 0 | 0 | **1** |
| **Eastern** | Afram Plains | 0 | 1 | 0 | 0 | **1** |
| | Manya Krobo | 1 | 0 | 0 | 0 | **1** |
| | **Eastern Total** | 1 | 1 | 0 | 0 | **2** |
| **Volta** | Adaklu Anyigbe | 0 | 0 | 1 | 0 | **1** |
| | **Volta Total** | 0 | 0 | 1 | 0 | **1** |
| **Provincial Total** | | 2 | 2 | 1 | 1 | **6** |

EORIC - UENR copy

*Daily Fire Detection count > 50 are highlighted in orange and Fire detection >=100 are highlighted in red.*

| Sep-2016 | | 06/09/16 | 11/09/16 | 18/09/16 | Monthly Total |
|---|---|---|---|---|---|
| **Upper West** | Wa | 1 | 0 | 0 | **1** |
| | **Upper West Total** | 1 | 0 | 0 | **1** |
| **Volta** | Keta | 0 | 1 | 0 | **1** |
| | **Volta Total** | 0 | 1 | 0 | **1** |
| **Western** | Jomoro | 0 | 0 | 2 | **2** |
| | **Western Total** | 0 | 0 | 2 | **2** |
| **Provincial Total** | | **1** | **1** | **2** | **4** |

EORIC - UENR copy

*Daily Fire Detection count > 50 are highlighted in orange and Fire detection >=100 are highlighted in red.*

| Oct-2016 | | 06/10/16 | 14/10/16 | 15/10/16 | 17/10/16 | 18/10/16 | 22/10/16 | 23/10/16 | 24/10/16 | 25/10/16 | 26/10/16 | 27/10/16 | 29/10/16 | 30/10/16 | Monthly Total |
|---|---|---|---|---|---|---|---|---|---|---|---|---|---|---|---|
| **Ashanti** | Adansi North | 0 | 0 | 0 | 0 | 0 | 0 | 0 | 0 | 1 | 0 | 0 | 0 | 0 | **1** |
| | **Ashanti Total** | 0 | 0 | 0 | 0 | 0 | 0 | 0 | 0 | 1 | 0 | 0 | 0 | 0 | **1** |
| **Brong Ahafo** | Pru | 0 | 0 | 0 | 0 | 0 | 0 | 0 | 0 | 0 | 0 | 0 | 2 | 0 | **2** |
| | **Brong Ahafo Total** | 0 | 0 | 0 | 0 | 0 | 0 | 0 | 0 | 0 | 0 | 0 | 2 | 0 | **2** |
| **Greater Accra** | Dangbe East | 0 | 0 | 0 | 0 | 0 | 0 | 0 | 0 | 1 | 0 | 0 | 0 | 0 | **1** |
| | Dangbe West | 1 | 0 | 0 | 0 | 0 | 0 | 0 | 0 | 0 | 0 | 0 | 0 | 0 | **1** |
| | Tema | 0 | 0 | 0 | 1 | 0 | 0 | 0 | 0 | 0 | 0 | 0 | 0 | 0 | **1** |
| | **Greater Accra Total** | 1 | 0 | 0 | 1 | 0 | 0 | 0 | 0 | 1 | 0 | 0 | 0 | 0 | **3** |
| **Northern** | Bole | 0 | 0 | 0 | 0 | 0 | 0 | 0 | 0 | 0 | 0 | 1 | 0 | 0 | **1** |
| | Bunkpurugu Yunyoo | 0 | 0 | 0 | 0 | 0 | 0 | 0 | 0 | 0 | 0 | 0 | 1 | 0 | **1** |
| | Central Gonja | 0 | 0 | 0 | 0 | 0 | 0 | 0 | [illegible] | 0 | 0 | 1 | 0 | 0 | **1** |
| | East Gonja | 0 | 0 | 0 | 0 | 0 | [illegible] | 0 | 0 | 0 | 0 | 1 | 1 | 0 | **2** |
| | Gushiegu | 0 | 0 | 0 | 0 | 0 | 0 | 0 | 0 | 0 | 0 | 3 | 1 | 0 | **4** |
| | Nanumba North | 0 | 0 | 0 | 0 | 0 | 1 | 0 | 0 | 0 | 0 | 1 | 1 | 0 | **3** |
| | Nanumba South | 0 | [illegible] | 0 | 0 | 0 | 0 | 0 | 0 | 0 | 0 | 0 | 4 | 0 | **4** |
| | Saboba Chereponi | 0 | 0 | 0 | 0 | 0 | 0 | 0 | 0 | 0 | 0 | 0 | 4 | 0 | **4** |

EORIC - UENR copy

*Daily Fire Detection count > 50 are highlighted in orange and Fire detection >=100 are highlighted in red.*

| Oct-2016 | | 06/10/16 | 14/10/16 | 15/10/16 | 17/10/16 | 18/10/16 | 22/10/16 | 23/10/16 | 24/10/16 | 25/10/16 | 26/10/16 | 27/10/16 | 29/10/16 | 30/10/16 | Monthly Total |
|---|---|---|---|---|---|---|---|---|---|---|---|---|---|---|---|
| **Northern** | West Gonja | 0 | 0 | 0 | 0 | 0 | 0 | 0 | 0 | 0 | 0 | 1 | 0 | 0 | **1** |
| | West Mamprusi | 0 | 0 | 1 | 2 | 1 | 0 | 3 | 0 | 0 | 0 | 6 | 2 | 1 | **16** |
| | Yendi | 0 | 0 | 1 | 0 | 0 | 0 | 0 | 0 | 0 | 0 | 1 | 0 | 0 | **2** |
| | Zabzugu Tatale | 0 | 0 | 0 | 0 | 0 | 0 | 0 | 0 | 0 | 0 | 0 | 1 | 0 | **1** |
| | **Northern Total** | 0 | 0 | 2 | 2 | 1 | 1 | 3 | 0 | 0 | 0 | 15 | 15 | 1 | **40** |
| **Upper East** | Bawku West | 0 | 0 | 0 | 0 | 0 | 0 | 0 | 0 | 0 | 0 | 0 | 1 | 0 | **1** |
| | Bolgatanga | 0 | 0 | 0 | 0 | 0 | 0 | 0 | 0 | 0 | 0 | 1 | 0 | 0 | **1** |
| | Bongo | 0 | 0 | 0 | 0 | 0 | 0 | 0 | 0 | 1 | 1 | 1 | 0 | 0 | **3** |
| | Builsa | 0 | 0 | 0 | 0 | 0 | 0 | 0 | 0 | 1 | 2 | 3 | 1 | 0 | **7** |
| | Garu Tempane | 0 | 0 | 0 | 0 | 0 | 0 | 0 | 0 | 0 | 0 | 1 | 2 | | **3** |
| | Kassena Nankana | 0 | 0 | 0 | 0 | 0 | 0 | 0 | 0 | 2 | 0 | 2 | 4 | 0 | **8** |
| | Talensi Nabdam | 0 | 0 | 0 | 0 | 0 | 0 | 0 | 0 | 0 | 0 | 1 | 2 | 0 | **3** |
| | **Upper East Total** | 0 | 0 | 0 | 0 | 0 | 0 | 0 | 0 | 4 | 3 | 9 | 10 | 0 | **26** |
| **Upper West** | Jirapa Lambussie | 0 | 0 | 0 | 0 | 0 | 0 | 0 | | 3 | 1 | 1 | 0 | 0 | **5** |
| | Lawra | 0 | 0 | 0 | 0 | 0 | | 0 | 0 | 5 | 0 | 1 | 0 | 0 | **6** |
| | Sissala East | 0 | 0 | 0 | 0 | 0 | 0 | 0 | 0 | 0 | 0 | 1 | 0 | 0 | **1** |
| | Sissala West | 0 | 0 | 0 | | | 0 | 0 | 1 | 1 | 0 | 0 | 0 | 0 | **2** |
| | Wa | 0 | | 0 | 0 | 0 | 0 | 0 | 0 | 0 | 0 | 0 | 0 | 0 | **1** |
| | Wa West | 0 | 0 | 0 | 0 | 0 | 0 | 0 | 0 | 0 | 0 | 2 | 0 | 0 | **2** |
| | **Upper West Total** | 0 | 1 | 0 | 0 | 0 | 0 | 0 | 1 | 9 | 1 | 5 | 0 | 0 | **17** |

EORIC - UENR copy

*Daily Fire Detection count > 50 are highlighted in orange and Fire detection >=100 are highlighted in red.*

| Oct-2016 | | 06/10/16 | 14/10/16 | 15/10/16 | 17/10/16 | 18/10/16 | 22/10/16 | 23/10/16 | 24/10/16 | 25/10/16 | 26/10/16 | 27/10/16 | 29/10/16 | 30/10/16 | Monthly Total |
|---|---|---|---|---|---|---|---|---|---|---|---|---|---|---|---|
| **Volta** | Hohoe | 0 | 0 | 0 | 0 | 0 | 0 | 0 | 0 | 2 | 0 | 0 | 1 | 0 | **3** |
| | Keta | 0 | 0 | 0 | 0 | 1 | 0 | 0 | 0 | 0 | 0 | 0 | 0 | 0 | **1** |
| | Krachi | 0 | 0 | 0 | 0 | 0 | 0 | 0 | 0 | 0 | 0 | 0 | 1 | 0 | **1** |
| | Nkwanta | 0 | 0 | 0 | 0 | 0 | 0 | 0 | 0 | 0 | 0 | 0 | 3 | 0 | **3** |
| | **Volta Total** | 0 | 0 | 0 | 0 | 1 | 0 | 0 | 0 | 2 | 0 | 0 | 5 | 0 | **8** |
| **Provincial Total** | | **1** | **1** | **2** | **3** | **2** | **1** | **3** | **1** | **17** | **4** | **29** | **32** | **1** | **97** |

EORIC - UENR copy

*Daily Fire Detection count > 50 are highlighted in orange and Fire detection >=100 are highlighted in red.*

| Nov-2016 | | 01/11/16 | 02/11/16 | 03/11/16 | 07/11/16 | 08/11/16 | 09/11/16 | 10/11/16 | 12/11/16 | 13/11/16 | 14/11/16 | 15/11/16 | 16/11/16 | 17/11/16 | 18/11/16 | 19/11/16 | 20/11/16 | 21/11/16 | 22/11/16 | 23/11/16 | 24/11/16 | 25/11/16 | 26/11/16 | 7/11/16 | 28/11/16 | 29/11/16 | 30/11/16 | Monthly Total |
|---|---|---|---|---|---|---|---|---|---|---|---|---|---|---|---|---|---|---|---|---|---|---|---|---|---|---|---|---|
| **Ashanti** | Asante Akim North | 0 | 0 | 0 | 0 | 0 | 0 | 0 | 0 | 0 | 0 | 0 | 1 | 0 | 0 | 2 | 0 | 0 | 0 | 0 | 0 | 0 | 0 | 0 | 0 | 0 | 0 | **3** |
| | Sekyere East | 0 | 0 | 0 | 0 | 0 | 0 | 0 | 0 | 0 | 0 | 0 | 0 | 1 | 0 | 1 | 0 | 0 | 0 | 0 | 0 | 1 | 0 | 0 | 1 | 0 | 0 | **4** |
| | Sekyere West | 0 | 0 | 0 | 2 | 0 | 0 | 0 | 0 | 0 | 0 | 0 | 0 | 0 | 0 | 1 | 0 | 0 | 0 | 0 | 0 | 0 | 0 | 0 | 0 | 0 | 0 | **3** |
| | **Ashanti Total** | 0 | 0 | 0 | 2 | 0 | 0 | 0 | 0 | 0 | 0 | 0 | 1 | 1 | 0 | 4 | 0 | 0 | 0 | 0 | 0 | 1 | 0 | 0 | 1 | 0 | 0 | **10** |
| **Brong Ahafo** | Atebubu-Amantin | 0 | 0 | 0 | 0 | 0 | 0 | 0 | 0 | 0 | 7 | 0 | 0 | 0 | 0 | 1 | | 0 | 0 | 0 | 0 | 0 | 0 | 0 | 0 | 0 | 3 | **11** |
| | Jaman North | 0 | 0 | 0 | 0 | 0 | 0 | 0 | 0 | 0 | 0 | 0 | 0 | 0 | 0 | 0 | 0 | 0 | 0 | 0 | 0 | 0 | 1 | 0 | 0 | 0 | 0 | **1** |
| | Jaman South | 0 | 0 | 0 | 0 | 0 | 0 | 0 | 0 | 0 | 1 | 0 | 0 | 1 | 0 | 0 | 0 | 0 | 0 | 0 | 0 | 0 | 1 | 0 | 0 | 0 | 0 | **3** |
| | Kintampo North | 0 | 0 | 2 | 7 | 0 | 0 | 0 | 0 | 0 | 4 | 0 | 6 | 14 | 1 | 10 | 17 | 4 | 0 | 1 | 0 | 14 | 12 | 0 | 10 | 0 | 4 | **106** |
| | Kintampo South | 0 | 0 | 0 | 0 | 0 | 0 | 0 | 0 | 0 | 1 | 0 | 0 | 0 | 0 | 0 | 0 | 0 | 0 | 0 | 0 | 1 | 0 | 0 | 1 | 0 | 0 | **3** |
| | Nkoranza | 0 | 0 | 0 | 0 | 0 | 0 | 0 | 0 | 0 | 2 | 0 | 0 | 0 | 0 | 0 | 0 | 3 | 0 | 1 | 0 | 0 | 0 | 0 | 1 | 0 | 0 | **7** |
| | Pru | 0 | 0 | 0 | 4 | 0 | 1 | 1 | | 0 | 15 | 1 | 4 | 1 | 0 | 12 | 2 | 4 | 0 | 5 | 0 | 10 | 7 | 0 | 6 | 0 | 0 | **73** |
| | Sene | 0 | 0 | 0 | 2 | 0 | | 0 | 0 | 0 | 7 | 0 | 1 | 0 | 0 | 1 | 1 | 2 | 0 | 7 | 0 | 5 | 3 | 0 | 29 | 0 | 1 | **62** |
| | Tain | 0 | 0 | 0 | 0 | 0 | 0 | 5 | 0 | 0 | 4 | 1 | 4 | 0 | 0 | 17 | 1 | 1 | 0 | 0 | 0 | 2 | 0 | 0 | 3 | 0 | 0 | **38** |
| | Techiman | 0 | 0 | 0 | 0 | 0 | 0 | 0 | 0 | 0 | 2 | 0 | 0 | 0 | 0 | 0 | 0 | 0 | 0 | 0 | 0 | 0 | 0 | 0 | 0 | 0 | 0 | **2** |
| | **Brong Ahafo Total** | 0 | 0 | 2 | 13 | 0 | 4 | 6 | 0 | 0 | 43 | 2 | 15 | 16 | 1 | 41 | 21 | 14 | 0 | 14 | 0 | 32 | 24 | 0 | 50 | 0 | 8 | **306** |

EORIC - UENR COPY

*Daily Fire Detection count > 50 are highlighted in orange and Fire detection >=100 are highlighted in red.*

| Nov-2016 | | 01/11/16 | 02/11/16 | 03/11/16 | 07/11/16 | 08/11/16 | 09/11/16 | 10/11/16 | 12/11/16 | 13/11/16 | 14/11/16 | 15/11/16 | 16/11/16 | 17/11/16 | 18/11/16 | 19/11/16 | 20/11/16 | 21/11/16 | 22/11/16 | 23/11/16 | 24/11/16 | 25/11/16 | 26/11/16 | 27/11/16 | 28/11/16 | 9/11/16 | 30/11/16 | Monthly Total |
|---|---|---|---|---|---|---|---|---|---|---|---|---|---|---|---|---|---|---|---|---|---|---|---|---|---|---|---|---|
| **Central** | Gomoa | 0 | 0 | 0 | 0 | 0 | 0 | 0 | 0 | 0 | 1 | 0 | 0 | 0 | 0 | 0 | 0 | 0 | 0 | 0 | 0 | 2 | 0 | 0 | 0 | 0 | 0 | **3** |
| | **Central Total** | 0 | 0 | 0 | 0 | 0 | 0 | 0 | 0 | 0 | 1 | 0 | 0 | 0 | 0 | 0 | 0 | 0 | 0 | 0 | 0 | 2 | 0 | 0 | 0 | 0 | 0 | **3** |
| **Eastern** | Afram Plains | 0 | 0 | 0 | 0 | 0 | 0 | 0 | 0 | 0 | 0 | 0 | 0 | 0 | 0 | 0 | 0 | 0 | 0 | 6 | 0 | | 0 | | 6 | 0 | 0 | **13** |
| | Asuogyaman | 0 | 0 | 0 | 0 | 0 | 0 | 0 | 0 | 0 | 0 | 0 | 0 | 0 | 0 | 0 | 0 | 0 | 0 | 0 | 0 | 0 | 0 | 0 | 1 | 0 | 0 | **1** |
| | Fanteakwa | 0 | 0 | 0 | 0 | 0 | 0 | 0 | 0 | 0 | 1 | 0 | 1 | 0 | 0 | 2 | 0 | 0 | 0 | | 0 | 0 | 0 | 0 | 0 | 0 | 1 | **5** |
| | Kwahu South | 0 | 0 | 0 | 0 | 0 | 0 | 0 | 0 | 0 | 0 | 0 | 0 | 0 | 0 | 0 | 0 | 0 | 0 | | 0 | 1 | 0 | 0 | 0 | 0 | 0 | **1** |
| | **Eastern Total** | 0 | 0 | 0 | 0 | 0 | 0 | 0 | 0 | 0 | 1 | 0 | 1 | 0 | 0 | 2 | 0 | 0 | 0 | 6 | 0 | 2 | 0 | 0 | 7 | 0 | 1 | **20** |
| **Greater Accra** | Accra | 0 | 1 | 0 | 0 | 0 | 0 | 0 | 0 | 0 | 0 | 0 | 0 | 0 | 0 | | 0 | 0 | 0 | 0 | 0 | 0 | 0 | 0 | 0 | 0 | 0 | **1** |
| | **Greater Accra Total** | 0 | 1 | 0 | 0 | 0 | 0 | 0 | 0 | 0 | 0 | 0 | 0 | 0 | 0 | 0 | 0 | 0 | 0 | 0 | 0 | 0 | 0 | 0 | 0 | 0 | 0 | **1** |
| **Northern** | Bole | 0 | 0 | 0 | 0 | 0 | 0 | 0 | 0 | 0 | 6 | 2 | 0 | 8 | 0 | 5 | 1 | 1 | 0 | 0 | 1 | 2 | 2 | 0 | 7 | 0 | 1 | **46** |
| | Bunkpurugu Yunyoo | 0 | 0 | 1 | 1 | 0 | 0 | 2 | 0 | 0 | 5 | 0 | 8 | 2 | 0 | 3 | 2 | 1 | 0 | 0 | 0 | 0 | 0 | 0 | 1 | 1 | 0 | **27** |
| | Central Gonja | 0 | 0 | 0 | 2 | 2 | 5 | 2 | 0 | 0 | 14 | 0 | 10 | 22 | 3 | 10 | 4 | 0 | 0 | 10 | 5 | 5 | 18 | 0 | 13 | 2 | 6 | **133** |
| | East Gonja | 0 | 0 | 0 | 12 | 0 | 2 | 3 | 1 | 0 | 32 | 0 | 25 | 7 | 4 | 16 | 7 | 15 | 0 | 20 | 1 | 37 | 33 | 0 | 25 | 2 | 6 | **248** |
| | East Mamprusi | 0 | 0 | 0 | 1 | 0 | 0 | 0 | 3 | 0 | 3 | 0 | 8 | 2 | 4 | 9 | 0 | 4 | 0 | 4 | 0 | 1 | 1 | 0 | 0 | 0 | 0 | **40** |
| | Gushiegu | 0 | 0 | 6 | 5 | 2 | 1 | 4 | 0 | 1 | 15 | 0 | 15 | 7 | 3 | 6 | 3 | 1 | 0 | 3 | 0 | 1 | 0 | 0 | 1 | 0 | 0 | **74** |
| | Karaga | 0 | 0 | 0 | 4 | 0 | 3 | 11 | 3 | 0 | 12 | 0 | 12 | 3 | 7 | 1 | 1 | 3 | 0 | 2 | 0 | 1 | 0 | 0 | 2 | 0 | 4 | **69** |
| | Nanumba North | 0 | 0 | 0 | 5 | | 0 | 1 | 0 | 0 | 7 | 0 | 5 | 0 | 0 | 4 | 0 | 1 | 0 | 4 | 0 | 2 | 5 | 0 | 4 | 0 | 0 | **38** |
| | Nanumba South | 0 | 0 | 0 | 8 | 0 | 0 | 1 | 1 | 0 | 11 | 0 | 2 | 0 | 0 | 0 | 0 | 1 | 0 | 1 | 0 | 0 | 1 | 0 | 5 | 1 | 1 | **33** |
| | Saboba Chereponi | 0 | 0 | 1 | 23 | 0 | 2 | 3 | 4 | 0 | 5 | 0 | 2 | 0 | 0 | 0 | 4 | 1 | 0 | 3 | 0 | 3 | 5 | 0 | 1 | 2 | 5 | **64** |
| | Savelugu Nanton | 0 | 0 | 0 | 3 | 3 | 0 | 0 | 0 | 0 | 7 | 0 | 3 | 0 | 0 | 0 | 0 | 1 | 0 | 2 | 0 | 0 | 0 | 0 | 1 | 0 | 0 | **20** |

EORIC - UENR copy

*Daily Fire Detection count > 50 are highlighted in orange and Fire detection >=100 are highlighted in red.*

| Nov-2016 | | 01/11/16 | 02/11/16 | 03/11/16 | 07/11/16 | 08/11/16 | 09/11/16 | 10/11/16 | 12/11/16 | 13/11/16 | 14/11/16 | 15/11/16 | 16/11/16 | 17/11/16 | 18/11/16 | 19/11/16 | 20/11/16 | 21/11/16 | 22/11/16 | 23/11/16 | 24/11/16 | 25/11/16 | 26/11/16 | 27/11/16 | 28/11/16 | 9/11/16 | 30/11/16 | Monthly Total |
|---|---|---|---|---|---|---|---|---|---|---|---|---|---|---|---|---|---|---|---|---|---|---|---|---|---|---|---|---|
| **Northern** | Sawa-Tuna-Kalba | 0 | 0 | 0 | 0 | 1 | 6 | 26 | 2 | 0 | 33 | 8 | 41 | 37 | 19 | 38 | 7 | 17 | 0 | 19 | 1 | 40 | 2 | 0 | 20 | 0 | 0 | **317** |
| | Tamale | 0 | 0 | 0 | 2 | 0 | 0 | 0 | 0 | 0 | 1 | 0 | 0 | 0 | 0 | 0 | 0 | 0 | 0 | 0 | 0 | 0 | 0 | 0 | 0 | 0 | 0 | **3** |
| | Tolon-Kumbungu | 1 | 0 | 0 | 5 | 1 | 12 | 6 | 0 | 0 | 8 | 0 | 13 | 6 | 6 | 13 | 0 | 10 | 0 | 5 | 0 | | 1 | | 7 | 4 | 0 | **100** |
| | West Gonja | 0 | 0 | 0 | 0 | 3 | 4 | 4 | 0 | 0 | 41 | 2 | 23 | 13 | 8 | 21 | 6 | 7 | 0 | 26 | 7 | 33 | 7 | 0 | 14 | 1 | 3 | **223** |
| | West Mamprusi | 3 | 0 | 18 | 13 | 15 | 17 | 38 | 3 | 0 | 42 | 9 | 27 | 14 | 23 | 22 | 5 | 23 | 1 | 1 | 6 | 13 | 14 | 2 | 18 | 4 | 2 | **351** |
| | Yendi | 0 | 0 | 1 | 0 | 0 | 0 | 0 | 0 | 0 | 21 | 0 | 29 | 9 | 4 | 10 | 4 | 0 | 0 | 14 | 0 | 5 | 13 | 0 | 14 | 2 | 13 | **139** |
| | Zabzugu Tatale | 0 | 0 | 2 | 1 | 0 | 0 | 0 | 0 | 0 | 9 | 0 | 3 | 0 | 0 | 0 | 0 | 0 | 0 | 4 | 0 | 2 | 1 | 0 | 3 | 0 | 4 | **29** |
| | **Northern Total** | 4 | 0 | 29 | 85 | 27 | 52 | 101 | 17 | 1 | 272 | 21 | 226 | 140 | 8 | 1 8 | 44 | 86 | 1 | 136 | 21 | 147 | 103 | 2 | 136 | 19 | 45 | **1954** |
| **Upper East** | Bawku Municipal | 0 | 0 | 0 | 0 | 0 | 0 | 0 | 3 | 0 | 0 | 0 | 1 | 0 | | 3 | 0 | 0 | 0 | 3 | 0 | 0 | 0 | 0 | 2 | 0 | 0 | **12** |
| | Bawku West | 0 | 0 | 5 | 3 | 4 | 4 | 11 | 2 | 0 | 22 | 0 | 12 | 2 | 3 | 4 | 2 | 4 | 0 | 10 | 0 | 2 | 1 | 0 | 0 | 2 | 0 | **93** |
| | Bolgatanga | 0 | 0 | 1 | 0 | 0 | 0 | 5 | 0 | 0 | 6 | 0 | 0 | 2 | 0 | 0 | 4 | 3 | 0 | 0 | 0 | 0 | 0 | 0 | 0 | 0 | 2 | **23** |
| | Bongo | 0 | 0 | 0 | 0 | 0 | 0 | 0 | 0 | 0 | 0 | 0 | 0 | 0 | 1 | 0 | 0 | 3 | 0 | 0 | 0 | 0 | 0 | 0 | 0 | 0 | 0 | **4** |
| | Builsa | 4 | 0 | 5 | 3 | 3 | 29 | 6 | | 0 | 37 | 1 | 17 | 12 | 11 | 5 | 1 | 7 | 0 | 8 | 0 | 4 | 3 | 0 | 5 | 0 | 0 | **175** |
| | Garu Tempane | 0 | 0 | 0 | 0 | 0 | 0 | 2 | | 0 | 4 | 0 | 3 | 1 | 0 | 1 | 0 | 3 | 1 | 2 | 0 | 4 | 2 | 0 | 0 | 0 | 0 | **23** |
| | Kassena Nankana | 0 | 0 | 5 | 3 | 2 | 14 | 8 | 1 | 0 | 20 | 4 | 20 | 9 | 5 | 13 | 0 | 8 | 0 | 7 | 7 | 2 | 4 | 1 | 6 | 0 | 1 | **140** |
| | Talensi Nabdam | 0 | 0 | 2 | 0 | 0 | 6 | 3 | 0 | 0 | 11 | 0 | 5 | 7 | 13 | 1 | 1 | 5 | 0 | 1 | 0 | 0 | 0 | 0 | 4 | 1 | 2 | **62** |
| | **Upper East Total** | 4 | 0 | 18 | | | 53 | 45 | 10 | 0 | 100 | 5 | 58 | 33 | 33 | 27 | 8 | 33 | 1 | 31 | 7 | 12 | 10 | 1 | 17 | 3 | 5 | **532** |
| **Upper West** | Jirapa Lambussie | 0 | 0 | 2 | 0 | 3 | 6 | 18 | 14 | 0 | 10 | 0 | 9 | 5 | 2 | 5 | 0 | 3 | 0 | 2 | 1 | 1 | 4 | 0 | 2 | 0 | 0 | **87** |
| | Lawra | 0 | 0 | 3 | 1 | 2 | 5 | 20 | 7 | 0 | 6 | 0 | 2 | 11 | 3 | 1 | 0 | 1 | 0 | 9 | 0 | 0 | 1 | 0 | 0 | 0 | 0 | **72** |
| | Nadowli | 0 | 0 | 5 | 0 | 14 | 22 | 49 | 4 | 0 | 20 | 1 | 27 | 42 | 16 | 28 | 1 | 21 | 0 | 7 | 6 | 12 | 20 | 1 | 12 | 2 | 2 | **312** |

EORIC - UENR COPY

*Daily Fire Detection count > 50 are highlighted in orange and Fire detection >=100 are highlighted in red.*

| Nov-2016 | | 01/11/16 | 02/11/16 | 03/11/16 | 07/11/16 | 08/11/16 | 09/11/16 | 10/11/16 | 12/11/16 | 13/11/16 | 14/11/16 | 15/11/16 | 16/11/16 | 17/11/16 | 18/11/16 | 19/11/16 | 20/11/16 | 21/11/16 | 22/11/16 | 23/11/16 | 24/11/16 | 25/11/16 | 26/11/16 | 27/11/16 | 28/11/16 | 9/11/16 | 30/11/16 | Monthly Total |
|---|---|---|---|---|---|---|---|---|---|---|---|---|---|---|---|---|---|---|---|---|---|---|---|---|---|---|---|---|
| **Upper West** | Sissala East | 0 | 0 | 0 | 1 | 3 | 18 | 39 | 9 | 0 | 19 | 5 | 13 | 33 | 30 | 15 | 7 | 25 | 0 | 24 | 12 | 7 | 4 | 1 | 10 | 0 | 0 | **275** |
| | Sissala West | 0 | 0 | 2 | 1 | 6 | 16 | 32 | 18 | 0 | 43 | 0 | 30 | 23 | 9 | 26 | 7 | 18 | 2 | 17 | 2 | 4 | 9 | 4 | 0 | 4 | 0 | **273** |
| | Wa | 0 | 0 | 0 | 0 | 0 | 2 | 11 | 0 | 0 | 12 | 1 | 15 | 25 | 4 | 17 | 4 | 8 | 0 | 4 | 6 | 11 | 8 | [illegible] | 11 | 2 | 5 | **146** |
| | Wa East | 0 | 0 | 3 | 2 | 1 | 5 | 27 | 5 | 0 | 35 | 0 | 13 | 30 | 8 | 27 | 2 | 8 | 0 | 8 | 0 | 13 | 4 | 0 | 31 | 0 | 3 | **225** |
| | Wa West | 0 | 0 | 1 | 0 | 2 | 12 | 12 | 0 | 0 | 6 | 4 | 6 | 8 | 4 | 11 | 0 | 4 | 0 | [illegible] | 1 | 3 | 10 | 0 | 3 | 0 | 0 | **87** |
| | **Upper West Total** | 0 | 0 | 16 | 5 | 31 | 86 | 208 | 57 | 0 | 151 | 11 | 115 | 177 | 76 | 130 | 21 | 88 | 2 | 71 | 28 | 51 | 60 | 6 | 69 | 8 | 10 | **1477** |
| **Volta** | Adaklu Anyigbe | 0 | 0 | 0 | 0 | 0 | 0 | 0 | 0 | 0 | 2 | 0 | 0 | 0 | 0 | 0 | 0 | 0 | 0 | 0 | 0 | 0 | 0 | 0 | 0 | 0 | 0 | **2** |
| | Hohoe | 0 | 0 | 1 | 1 | 0 | 0 | 0 | 0 | 0 | 0 | 0 | 0 | 0 | 0 | [illegible] | 0 | 0 | 0 | 0 | 0 | 0 | 0 | 0 | 1 | 0 | 0 | **3** |
| | Jasikan | 0 | 0 | 0 | 1 | 0 | 0 | 0 | 0 | 0 | 0 | 0 | 0 | 0 | [illegible] | 0 | 0 | 0 | 0 | 0 | 0 | 0 | 0 | 0 | 0 | 0 | 0 | **1** |
| | Ketu | 0 | 0 | 0 | 1 | 0 | 0 | 0 | 0 | 0 | 0 | 0 | 0 | [illegible] | 0 | 0 | 0 | 0 | 0 | 0 | 0 | 0 | 0 | 0 | 0 | 0 | 0 | **1** |
| | Kpandu | 0 | 0 | 0 | 2 | 0 | 0 | 0 | 0 | 0 | 0 | 0 | 0 | 0 | 0 | 0 | 0 | 0 | 0 | 0 | 0 | 0 | 0 | 0 | 0 | 0 | 0 | **2** |
| | Krachi | 0 | 0 | 0 | 0 | 0 | 0 | 0 | 0 | 0 | 0 | 0 | 2 | 0 | 0 | 1 | 0 | 1 | 0 | 2 | 0 | 0 | 3 | 0 | 2 | 0 | 0 | **11** |
| | Krachi East | 0 | 0 | 0 | 1 | 0 | 0 | 2 | 0 | 0 | 1 | 0 | 3 | 0 | 0 | 0 | 0 | 0 | 0 | 0 | 0 | 0 | 0 | 0 | 1 | 0 | 0 | **8** |
| | Nkwanta | 0 | 0 | 0 | 13 | 0 | 1 | 0 | 2 | 0 | 12 | 0 | 11 | 1 | 0 | 2 | 0 | 0 | 0 | 3 | 0 | 2 | 0 | 0 | 2 | 0 | 1 | **50** |
| | **Volta Total** | 0 | 0 | 1 | 19 | 0 | [illegible] | 2 | 2 | 0 | 15 | 0 | 16 | 1 | 0 | 3 | 0 | 1 | 0 | 5 | 0 | 2 | 3 | 0 | 6 | 0 | 1 | **78** |
| **Western** | Mpohor Wassa East | 0 | 0 | 0 | 0 | 0 | 0 | 0 | 0 | 0 | 0 | 0 | 0 | 0 | 0 | 0 | 0 | 0 | 0 | 1 | 0 | 0 | 0 | 0 | 0 | 0 | 0 | **1** |
| | **Western Total** | 0 | 0 | 0 | 0 | 0 | 0 | 0 | 0 | 0 | 0 | 0 | 0 | 0 | 0 | 0 | 0 | 0 | 0 | 1 | 0 | 0 | 0 | 0 | 0 | 0 | 0 | **1** |
| **Provincial Total** | | **8** | **1** | **66** | **133** | **67** | **196** | **362** | **86** | **1** | **583** | **39** | **432** | **368** | **191** | **365** | **94** | **222** | **4** | **264** | **56** | **249** | **200** | **9** | **286** | **30** | **70** | **4382** |

EORIC - UENR COPY

*Daily Fire Detection count > 50 are highlighted in orange and Fire detection >=100 are highlighted in red.*

| Dec-2016 | | 01/12/16 | 02/12/16 | 03/12/16 | 04/12/16 | 05/12/16 | 06/12/16 | 07/12/16 | 08/12/16 | 09/12/16 | 10/12/16 | 11/12/16 | 12/12/16 | 13/12/16 | 14/12/16 | 15/12/16 | 16/12/16 | 17/12/16 | 18/12/16 | 19/12/16 | Monthly Total |
|---|---|---|---|---|---|---|---|---|---|---|---|---|---|---|---|---|---|---|---|---|---|
| **Ashanti** | Ahafo Ano North | 0 | 0 | 0 | 0 | 0 | 0 | 0 | 0 | 2 | 0 | 0 | 0 | 0 | 0 | 0 | 0 | 0 | 0 | 0 | **2** |
| | Amansie East | 0 | 0 | 0 | 0 | 0 | 0 | 0 | 0 | 0 | 0 | 0 | 0 | 0 | 0 | 0 | 1 | 0 | 0 | 0 | **1** |
| | Asante Akim North | 0 | 0 | 0 | 0 | 0 | 0 | 2 | 0 | 1 | 0 | 1 | 2 | 0 | 0 | 0 | 1 | 0 | 0 | 0 | **7** |
| | Asante Akim South | 0 | 0 | 0 | 0 | 0 | 0 | 0 | 0 | 2 | 0 | 0 | 0 | 0 | 0 | 0 | 0 | 0 | 0 | 0 | **2** |
| | Ejura Sekyedumas | 0 | 0 | 0 | 0 | 0 | 0 | 0 | 0 | 0 | 0 | 0 | 0 | 0 | 2 | 0 | 0 | 0 | 0 | 0 | **2** |
| | Sekyere East | 0 | 0 | 0 | 0 | 0 | 0 | 1 | 0 | 12 | 5 | 4 | 5 | 0 | 3 | 0 | 4 | 0 | 0 | 0 | **34** |
| | Sekyere West | 0 | 0 | 0 | 0 | 0 | 0 | 4 | 0 | 0 | 3 | 9 | 0 | 0 | 2 | 0 | 1 | 0 | 0 | 0 | **19** |
| | **Ashanti Total** | 0 | 0 | 0 | 0 | 0 | 0 | 7 | 0 | 17 | 8 | 14 | 7 | 0 | 7 | 0 | 7 | 0 | 0 | 0 | **67** |
| **Brong Ahafo** | Asutifi | 0 | 0 | 0 | 0 | 0 | 0 | 0 | 0 | 0 | 0 | 1 | 0 | 0 | 0 | 0 | 0 | 0 | 0 | 0 | **1** |
| | Atebubu-Amantin | 0 | 0 | 0 | 0 | 0 | 0 | 0 | 0 | 2 | 1 | 0 | 4 | 0 | | 0 | 2 | 0 | 0 | 0 | **13** |
| | Dormaa | 0 | 0 | 0 | 0 | 0 | 0 | 0 | 0 | 1 | 0 | 0 | 0 | 0 | 0 | 0 | 0 | 0 | 0 | 0 | **1** |
| | Jaman South | 0 | 0 | 0 | 0 | 0 | 0 | 0 | | 0 | 0 | 0 | 0 | 0 | 1 | 0 | 0 | 0 | 0 | 0 | **1** |
| | Kintampo North | 7 | 7 | 1 | 3 | 18 | 6 | 1 | 2 | 39 | 2 | 72 | 24 | 0 | 108 | 0 | 52 | 6 | 0 | 0 | **385** |
| | Kintampo South | 0 | 0 | 0 | 0 | 1 | 0 | 0 | 0 | 12 | 8 | 15 | 1 | 0 | 19 | 0 | 10 | 0 | 0 | 0 | **66** |
| | Nkoranza | 0 | 0 | 0 | 1 | 0 | 0 | 0 | 0 | 3 | 8 | 20 | 0 | 0 | 10 | 0 | 7 | 0 | 0 | 0 | **49** |
| | Pru | 1 | 0 | 0 | 1 | 6 | 2 | 14 | 0 | 29 | 12 | 50 | 11 | 0 | 59 | 0 | 32 | 5 | 0 | 0 | **222** |
| | Sene | 0 | 1 | 0 | 1 | 6 | 13 | 27 | 0 | 56 | 23 | 54 | 19 | 4 | 53 | 0 | 29 | 2 | 1 | 0 | **289** |
| | Tain | 0 | | 0 | 1 | 6 | 0 | 7 | 2 | 11 | 19 | 18 | 2 | 0 | 45 | 0 | 21 | 2 | 0 | 0 | **134** |

EORIC - UENR copy

*Daily Fire Detection count > 50 are highlighted in orange and Fire detection >=100 are highlighted in red.*

| Dec-2016 | | 01/12/16 | 02/12/16 | 03/12/16 | 04/12/16 | 05/12/16 | 06/12/16 | 07/12/16 | 08/12/16 | 09/12/16 | 10/12/16 | 11/12/16 | 12/12/16 | 13/12/16 | 14/12/16 | 15/12/16 | 16/12/16 | 17/12/16 | 18/12/16 | 19/12/16 | Monthly Total |
|---|---|---|---|---|---|---|---|---|---|---|---|---|---|---|---|---|---|---|---|---|---|
| **Brong Ahafo** | Techiman | 0 | 0 | 0 | 0 | 0 | 0 | 0 | 0 | 0 | 0 | 0 | 0 | 0 | 1 | 0 | 0 | 0 | 0 | 0 | **1** |
| | **Brong Ahafo Total** | 8 | 8 | 1 | 7 | 37 | 21 | 59 | 4 | 153 | 100 | 230 | 61 | 4 | 300 | 0 | 153 | 15 | 1 | 0 | **1162** |
| **Central** | Agona | 0 | 0 | 0 | 0 | 0 | 0 | 0 | 0 | 1 | 0 | 0 | 0 | 0 | 0 | 0 | 0 | 0 | 0 | 0 | **1** |
| | **Central Total** | 0 | 0 | 0 | 0 | 0 | 0 | 0 | 0 | 1 | 0 | 0 | 0 | 0 | 0 | 0 | 0 | 0 | 0 | 0 | **1** |
| **Eastern** | Afram Plains | 0 | 2 | 4 | 13 | 0 | 6 | 10 | 0 | 22 | 3 | 15 | 6 | 0 | 4 | 0 | 16 | 0 | 3 | [illegible] | **104** |
| | Fanteakwa | 0 | 0 | 0 | 0 | 0 | 0 | 0 | 0 | 0 | 0 | 1 | 0 | 0 | 0 | 0 | 0 | 0 | 0 | [illegible] | **1** |
| | Kwahu South | 0 | 0 | 0 | 0 | 0 | 0 | 2 | 0 | 2 | 0 | 0 | 0 | 0 | 0 | 0 | 1 | 0 | 0 | 0 | **5** |
| | Manya Krobo | 0 | 0 | 0 | 0 | 0 | 0 | 0 | 0 | 1 | 0 | 0 | 0 | 0 | 0 | [illegible] | 0 | 0 | 0 | 0 | **1** |
| | **Eastern Total** | 0 | 2 | 4 | 13 | 0 | 6 | 12 | 0 | 25 | 3 | 16 | 6 | 0 | [illegible] | 0 | 17 | 0 | 3 | 0 | **111** |
| **Greater Accra** | Dangbe East | 0 | 0 | 0 | 0 | 0 | 0 | 1 | 0 | 0 | 0 | 1 | 0 | [illegible] | 0 | 0 | 1 | 0 | 0 | 0 | **3** |
| | **Greater Accra Total** | 0 | 0 | 0 | 0 | 0 | 0 | 1 | 0 | 0 | 0 | 1 | 0 | 0 | 0 | 0 | 1 | 0 | 0 | 0 | **3** |
| **Northern** | Bole | 4 | 4 | 2 | 21 | 20 | 1 | 19 | 6 | 12 | 72 | 48 | 4 | 0 | 125 | 0 | 46 | 7 | 0 | 9 | **400** |
| | Bunkpurugu Yunyoo | 1 | 5 | 3 | 3 | 1 | 3 | 7 | 0 | 14 | 1 | 19 | 1 | 0 | 1 | 0 | 5 | 0 | 7 | 0 | **71** |
| | Central Gonja | 10 | 3 | 11 | 6 | 42 | 12 | 45 | 1 | 15 | 68 | 129 | 43 | 0 | 74 | 1 | 62 | 19 | 1 | 0 | **542** |
| | East Gonja | 12 | 2 | 6 | 11 | 33 | 7 | 52 | 4 | 76 | 57 | 94 | 32 | 0 | 100 | 0 | 87 | 5 | 0 | 0 | **598** |
| | East Mamprusi | 0 | 4 | 4 | 5 | 0 | 2 | 10 | 1 | 8 | 0 | 1 | 0 | 0 | 1 | 0 | 2 | 1 | 0 | 0 | **39** |
| | Gushiegu | 0 | 10 | 3 | 6 | [illegible] | 2 | 8 | 0 | 11 | 4 | 11 | 2 | 0 | 15 | 3 | 10 | 1 | 1 | 0 | **89** |
| | Karaga | 3 | 3 | 4 | 6 | 9 | 7 | 21 | 5 | 22 | 5 | 20 | 4 | 0 | 11 | 3 | 5 | 3 | 5 | 0 | **136** |
| | Nanumba North | 0 | 0 | 3 | 6 | 1 | 5 | 9 | 0 | 0 | 8 | 14 | 5 | 0 | 6 | 0 | 6 | 0 | 1 | 0 | **64** |
| | Nanumba South | 0 | 0 | 0 | 2 | 0 | 0 | 2 | 0 | 0 | 3 | 6 | 0 | 0 | 4 | 0 | 1 | 0 | 0 | 0 | **18** |

EORIC - UENR copy

*Daily Fire Detection count > 50 are highlighted in orange and Fire detection >=100 are highlighted in red.*

| Dec-2016 | | 01/12/16 | 02/12/16 | 03/12/16 | 04/12/16 | 05/12/16 | 06/12/16 | 07/12/16 | 08/12/16 | 09/12/16 | 10/12/16 | 11/12/16 | 12/12/16 | 13/12/16 | 14/12/16 | 15/12/16 | 16/12/16 | 17/12/16 | 18/12/16 | 19/12/16 | Monthly Total |
|---|---|---|---|---|---|---|---|---|---|---|---|---|---|---|---|---|---|---|---|---|---|
| **Northern** | Saboba Chereponi | 5 | 3 | 6 | 9 | 2 | 2 | 3 | 0 | 10 | 3 | 21 | 2 | 0 | 1 | 3 | 27 | 2 | 0 | 0 | **99** |
| | Savelugu Nanton | 0 | 1 | 1 | 0 | 1 | 0 | 7 | 0 | 8 | 14 | 21 | 2 | 0 | 5 | 0 | 10 | 0 | 0 | 0 | **70** |
| | Sawa-Tuna-Kalba | 52 | 7 | 13 | 27 | 26 | 10 | 35 | 12 | 45 | 72 | 59 | 18 | 2 | 70 | 0 | 56 | 10 | 0 | 18 | **532** |
| | Tamale | 0 | 0 | 2 | 1 | 0 | 0 | 0 | 0 | 0 | 0 | 6 | 0 | 0 | 5 | 0 | 1 | 0 | 0 | 0 | **15** |
| | Tolon-Kumbungu | 3 | 2 | 3 | 5 | 11 | 4 | 14 | 0 | 7 | 8 | 8 | 0 | 0 | 6 | 1 | 20 | 3 | 0 | [illegible] | **95** |
| | West Gonja | 61 | 8 | 22 | 38 | 63 | 36 | 55 | 29 | 51 | 158 | 193 | 68 | 10 | 103 | 7 | 104 | 31 | 0 | [illegible] | **1037** |
| | West Mamprusi | 25 | 28 | 11 | 17 | 31 | 9 | 37 | 1 | 47 | 3 | 21 | 11 | 0 | 15 | 1 | 53 | 6 | 2 | 0 | **318** |
| | Yendi | 0 | 1 | 12 | 6 | 2 | 5 | 21 | 0 | 3 | 27 | 53 | 13 | 0 | 1 | [illegible] | 16 | 0 | 2 | 0 | **172** |
| | Zabzugu Tatale | 0 | 0 | 1 | 6 | 6 | 0 | 13 | 0 | 9 | 6 | 8 | 2 | 0 | 1 | 0 | 2 | 0 | 0 | 0 | **54** |
| | **Northern Total** | 176 | 81 | 107 | 175 | 250 | 125 | 358 | 59 | 338 | 509 | 732 | 207 | 2 | 55 | 19 | 513 | 88 | 19 | 27 | **4349** |
| **Upper East** | Bawku Municipal | 0 | 0 | 0 | 2 | 0 | 0 | 0 | 0 | 0 | 0 | 0 | 0 | 0 | 0 | 0 | 0 | 0 | 0 | 0 | **2** |
| | Bawku West | 2 | 0 | 0 | 0 | 1 | 0 | 0 | 2 | 1 | 0 | 0 | 3 | 0 | 0 | 0 | 0 | 0 | 0 | 0 | **9** |
| | Bolgatanga | 0 | 0 | 0 | 0 | 0 | 0 | 2 | 0 | 0 | 0 | 0 | 0 | 0 | 1 | 0 | 3 | 0 | 0 | 0 | **6** |
| | Bongo | 0 | 0 | 0 | 0 | 0 | 0 | 0 | [illegible] | 3 | 0 | 5 | 0 | 0 | 0 | 0 | 1 | 0 | 0 | 0 | **9** |
| | Builsa | 4 | 3 | 2 | 5 | 7 | 2 | 10 | 2 | 25 | 0 | 2 | 1 | 0 | 4 | 0 | 3 | 4 | 1 | 0 | **75** |
| | Garu Tempane | 0 | 2 | 1 | 0 | 1 | 1 | 0 | 0 | 1 | 0 | 0 | 0 | 0 | 2 | 0 | 1 | 0 | 0 | 0 | **9** |
| | Kassena Nankana | 2 | 1 | 2 | 6 | 12 | 1 | 6 | 7 | 4 | 0 | 1 | 3 | 0 | 3 | 0 | 2 | 0 | 0 | 0 | **50** |
| | Talensi Nabdam | 7 | 5 | 6 | 2 | 3 | 2 | 3 | 0 | 7 | 0 | 0 | 0 | 0 | 1 | 3 | 7 | 0 | 4 | 0 | **50** |
| | **Upper East Total** | 15 | 11 | 11 | 15 | 24 | 6 | 21 | 11 | 41 | 0 | 8 | 7 | 0 | 11 | 3 | 17 | 4 | 5 | 0 | **210** |

EORIC - UENR copy

*Daily Fire Detection count > 50 are highlighted in orange and Fire detection >=100 are highlighted in red.*

| Dec-2016 | | 01/12/16 | 02/12/16 | 03/12/16 | 04/12/16 | 05/12/16 | 06/12/16 | 07/12/16 | 08/12/16 | 09/12/16 | 10/12/16 | 11/12/16 | 12/12/16 | 13/12/16 | 14/12/16 | 15/12/16 | 16/12/16 | 17/12/16 | 18/12/16 | 19/12/16 | Monthly Total |
|---|---|---|---|---|---|---|---|---|---|---|---|---|---|---|---|---|---|---|---|---|---|
| **Upper West** | Jirapa Lambussie | 4 | 3 | 4 | 2 | 7 | 1 | 15 | 2 | 1 | 1 | 4 | 1 | 0 | 0 | 0 | 1 | 0 | 0 | 0 | **46** |
| | Lawra | 2 | 0 | 0 | 0 | 3 | 1 | 1 | 0 | 3 | 0 | 0 | 1 | 0 | 2 | 0 | 1 | 0 | 0 | 1 | **15** |
| | Nadowli | 17 | 7 | 33 | 8 | 16 | 3 | 23 | 6 | 15 | 6 | 0 | 16 | 1 | 5 | 0 | 9 | 8 | 0 | 5 | **178** |
| | Sissala East | 29 | 15 | 12 | 15 | 43 | 4 | 16 | 16 | 54 | 10 | 21 | 13 | 0 | 29 | 0 | 45 | 7 | 0 | 0 | **329** |
| | Sissala West | 40 | 14 | 17 | 18 | 21 | 0 | 35 | 10 | 35 | 3 | 22 | 14 | 1 | 10 | 0 | 23 | 6 | 0 | | **269** |
| | Wa | 15 | 3 | 22 | 11 | 18 | 0 | 15 | 5 | 36 | 3 | 7 | 6 | 0 | 8 | 0 | 21 | 7 | 0 | | **179** |
| | Wa East | 25 | 5 | 19 | 9 | 7 | 4 | 4 | 6 | 45 | 14 | 9 | 0 | 0 | 4 | 0 | 20 | 7 | 0 | 0 | **178** |
| | Wa West | 2 | 0 | 5 | 6 | 6 | 0 | 12 | 0 | 15 | 2 | 4 | 2 | 0 | 9 | | 1 | 3 | 0 | 5 | **72** |
| | **Upper West Total** | 134 | 47 | 112 | 69 | 121 | 13 | 121 | 45 | 204 | 39 | 67 | 53 | 2 | 6 | 0 | 121 | 38 | 0 | 13 | **1266** |
| **Volta** | Adaklu Anyigbe | 0 | 0 | 0 | 0 | 0 | 0 | 1 | 0 | 1 | 0 | 0 | 0 | | 0 | 0 | 2 | 0 | 0 | 0 | **4** |
| | Ho | 0 | 0 | 0 | 0 | 0 | 0 | 0 | 0 | 0 | 1 | 0 | 0 | 0 | 0 | 0 | 0 | 0 | 0 | 0 | **1** |
| | Hohoe | 0 | 0 | 0 | 0 | 0 | 0 | 0 | 0 | 1 | 0 | 0 | 0 | 0 | 0 | 0 | 0 | 0 | 0 | 0 | **1** |
| | Krachi | 0 | 0 | 0 | 0 | 0 | 2 | 2 | 0 | 3 | 0 | 0 | 0 | 0 | 4 | 0 | 5 | 0 | 0 | 0 | **16** |
| | Nkwanta | 0 | 0 | 0 | 2 | 1 | 0 | 5 | | 20 | 1 | 2 | 0 | 0 | 1 | 0 | 5 | 0 | 0 | 0 | **37** |
| | North Tongu | 0 | 0 | 0 | 0 | 0 | | 0 | 0 | 0 | 0 | 0 | 0 | 0 | 0 | 0 | 3 | 0 | 0 | 0 | **3** |
| | **Volta Total** | 0 | 0 | 0 | 2 | 1 | 2 | 8 | 0 | 25 | 2 | 2 | 0 | 0 | 5 | 0 | 15 | 0 | 0 | 0 | **62** |
| **Provincial Total** | | **333** | **149** | **235** | **281** | **433** | **173** | **587** | **119** | **804** | **661** | **1070** | **341** | **18** | **948** | **22** | **844** | **145** | **28** | **40** | 7231 |

EORIC - UENR copy

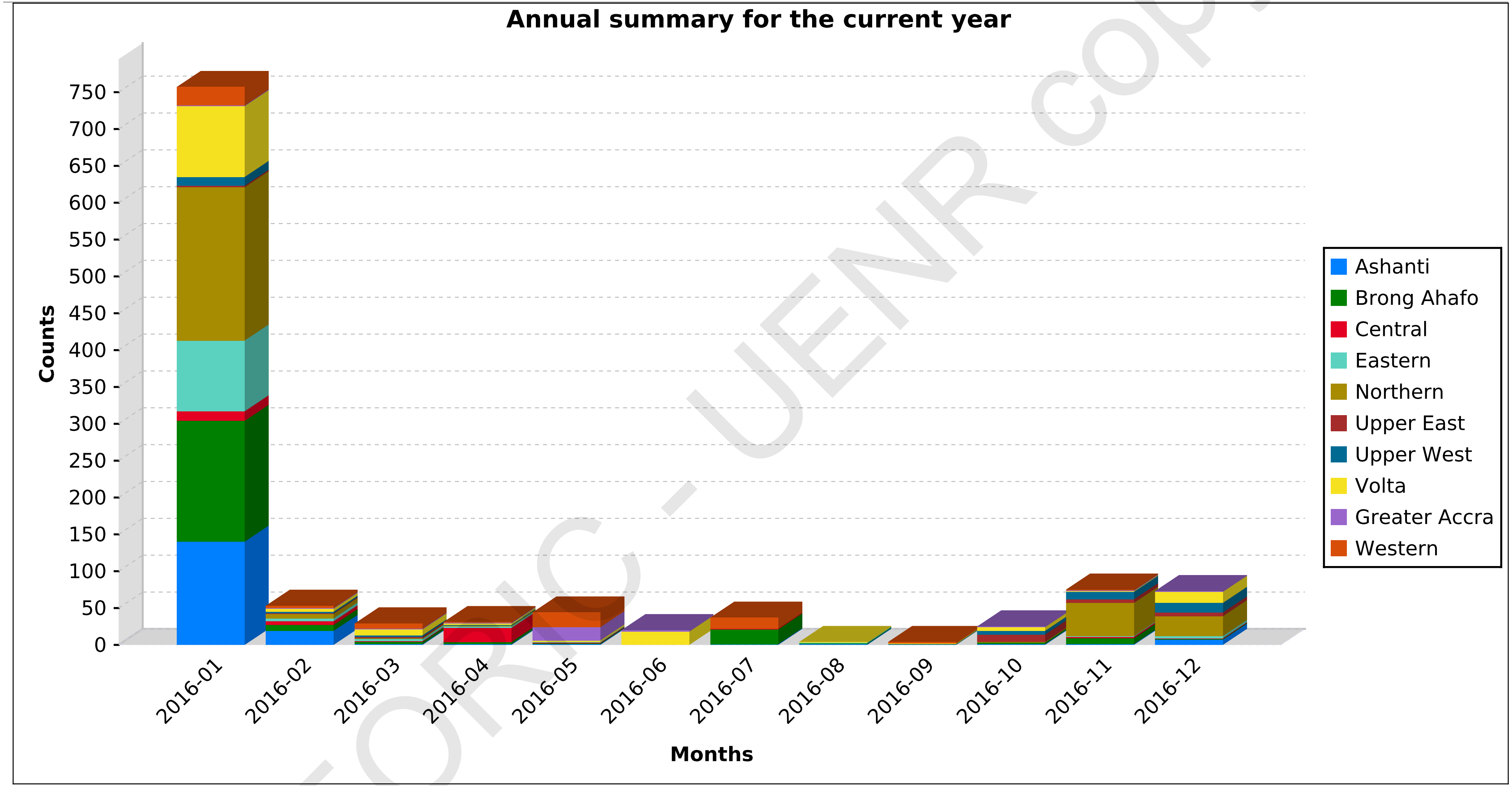
Annual summary for the current year
Counts
750
700
650
600
550
500
450
400
350
300
250
200
150
100
50
0
2016-01
2016-02
2016-03
2016-04
2016-05
2016-06
2016-07
2016-08
2016-09
2016-10
2016-11
2016-12
Months
Ashanti
Brong Ahafo
Central
Eastern
Northern
Upper East
Upper West
Volta
Greater Accra
Western
