# Supplementary material for: Fire use practices, knowledge and perceptions in a West African savanna parkland
Source: PLoS One. 2022 May 19;17(5):e0240271. doi: 10.1371/journal.pone.0240271 (PMC9119518; doi:10.1371/journal.pone.0240271)
Supplement: Attachment — Submitted filename: Response to Editor_june.docx [file pone.0240271.s008.docx]

Dear Editor,

Revision has been by uploading signed content permission from Earth Observation Research and Innovation Centre (EORIC) for the fire count data.

A few text changes have been made to reflect one signatory for the content permission:

Stratified, purposive and simple random sampling techniques were used. Data on daily fire counts (detected by sensors on Earth observation satellite) from 2013 to 2017 on Ghana, were received from EORIC (Earth Observation Research and Innovation Centre - https://eoric.uenr.edu.gh/?bunch_projects=fire-information-for-rural-farmers), Ghana in collaboration with the Advanced Fire Information System (AFIS: http://www.afis.co.za) of the CSIR, Meraka Institute (http://www.csir.co.za), South Africa. All fire count data for 2013 to 2017 were sent to me by the Director of EORIC via email. The fire count data for 2013 to 2017, was used to generate a fire map for the region (Fig 1 A) which is compared to the land use/land cover map of the region (Fig 1 B

The annotation for Figure 1:

A - Fire frequency gradient (colour gradient) of the study area and selected study districts. Source of data for maps – EORIC-AFIS under a CC BY license, with permission from EORIC, original copyright,2017. B. Land use map of the study area for 2017. Republished from [CERSGIS-SERVIR] under a CC BY license, with permission from SERVIR, original copyright, 2017.
