# Supplementary material for: Fire use practices, knowledge and perceptions in a West African savanna parkland
Source: PLoS One. 2022 May 19;17(5):e0240271. doi: 10.1371/journal.pone.0240271 (PMC9119518; doi:10.1371/journal.pone.0240271)
Supplement: Attachment — Submitted filename: Response to Reviewers.docx [file pone.0240271.s009.docx]

Dear Editor,

Response to Reviewers’ Comments

To the best our knowledge, we have revised the manuscript in response to the comments and contribution by the Reviewers and the Academic Editor. The responses to the reviewers and rebuttals are highlighted in blue. I have uploaded a Zip File of all the fire count data used for analysis and added the questionnaire at the end of the manuscript.

The following will be uploaded as requested:

• A rebuttal letter that responds to each point raised by the academic editor and reviewer(s). You should upload this letter as a separate file labeled 'Response to Reviewers'.

• A marked-up copy of your manuscript that highlights changes made to the original version. You should upload this as a separate file labeled 'Revised Manuscript with Track Changes'.

• An unmarked version of your revised paper without tracked changes. You should upload this as a separate file labeled 'Manuscript'.

Responses and rebuttals to reviewers’ comments are presented below:

First up, I don’t understand why you stratified your study sites in the Northern Region by fire hotspot frequency (as an index of fire activity) when a more logical approach would be to stratify firstly by major cultural grouping (e.g., Manprusi, Dagomba, Gonja…), noting that 92% of your responses came from these three groups, in order to explore similarities / differences in cultural fire management practices.

The study did not focus on cultural groupings. The various ethnic groupings in the north of Ghana do not have different fire management strategies. If we were comparing the savanna to the transition ecological zone or the coastal savanna of Ghana then we could do the cultural comparison. The Guinea savanna was chosen because it has recorded more fire occurrences than the south of Ghana. Thus, the import of the study was to ascertain people perception of fire use and fire practices, how the frequent fires affect economic species and soils in the area. Yes, we agree to your suggestion but again cultural groupings or ethnicity, did not make significant prediction to the reason for the use of fire, indicating that all the ethnic groups have similar reasons for the use of fire, especially for land preparation for cropping which is the major reasons for fire use across all the districts. Also, we did find the Dagomba ethnic in almost every district, although they may not be indigenes of the districts. It is difficult to directly link fire use to a particular cultural grouping in this case due to the heterogeneous nature of most of the district.

Are your hotspot frequency classes (high, medium, low) reflective of the cultural fire management activities of such cultural groupings, and/or population density generally, or some other factor(s)?

We considered hotspot because of the high fire occurrence during the dry season in the Guinea savanna. The fire count data showed varying fire counts in the various district hence the grouping into low medium and high fire frequency Zones. It is difficult to make clear distinctions in cultural fire management because they have similar practices. The reason why we find more people use fire for almost the same activities.

Given that the title of your ms purports to describe traditional practices, knowledge(s) and perceptions…, reframing the focus of your analysis would seem to me to make much more sense. Such an approach would also presumably help tease out some intriguing findings in your results; for example, I found it interesting that very little ‘fire control’ is practised in association with hunting, nor the implementation of firebreaks (Table 3)—doesn’t this lead to major wildfire problems and associated significant socio-cultural-economic issues? Does this reflect traditional practice in all cultural groupings?

No, we did not observe any difference in the vein. The differences in location in location or district is that some district have more fauna so they do hunt these animals in the dry season. However, people from other districts within the region move to hunt in these areas. This MS is a Chapter in a thesis which assessed the impact of frequent fires on woody species and soils in these fire hotspots in the Guinea savanna. That is why we wanted to find out what reasons and perceptions do people burn in order to give a justification of fire hotspots in the Guinea savanna.

Taking such an approach would provide more useful cultural contrasts than your current use of data stratified by hotspot fire frequency as presented in Tables 2,3,4—

I don’t know how you will consider this, but again our understanding of and focus on traditional fire use and management is more of using fire use without much regulation like using fire for farming, hunting, burning stubble after cropping with less attribution to cultural groupings. For instance, Huffman’s (2013) review on traditional fire management showed that attributes such as type and pattern of fire are less considered in the use of fire traditional fire management. In contrast fire applied in conservation sites such as the Mole National Park are well planned.

and I confess I don’t know what Table 5 was attempting to convey.

Table has been deleted.

Note also:

• any analysis of hotspot frequency data should be presented in Results, not in Methods as currently

Please it has been summarized in a table and has been provided under results.

You need to provide a copy of the questionnaire either as an appendix or in supplementary materials, including only those questions that you use for analysis of results.

Please find attached

• Figures need to be revisited and better presented, noting that Fig 1 is included again as Fig 1A. Given comments above why not include a map of the 5 Chiefdoms, especially to highlight the three for which you have adequate survey data.

We hope we have been able to explain the reason why we chose the hotspots for the analysis. Again, this paper is a Chapter in a thesis which assessed the impact of fire on woody species and soil in these fire hotspots. We can probably consider the cultural groupings in fire use in future by comparing the cultural/traditional fire knowledge in North to the south of Ghana. These have distinct cultural practices which is determined by factors such rainy season, which is unimodal in the North and bimodal in the South of Ghana.

Reviewer #5: I give some specific suggestions below, but overall, I am concerned that the paper is somewhat rambling and lacking a focus. It appears that the premise of the paper is that there is some objective impact of the fire regimes on the environment and interviews were conducted to see how close the respondents perceptions matched that objective reality. This is implicit in the conclusion about the benefit of education and training (line 564).

However the objective reality is not spelt out clearly and the line up against interview findings is laid out in a rambling hard-to-follow way. I would really like to see this relationship tightened up considerably.

The objective of the research has been clearly spelt out in the Introduction. Before this feedback from PLOS ONE we thought that a Multiple regression analysis would unravel some hidden details such as activities that fire is used for, reasons for fire use and fire use in the dry season and district, ethnic group, gender, age and education etc. I hope that the Multiple regression analysis adds to making a better analysis and presentation of the objectives of the research questions. Thus we changed the title to reflect data analysis and the results.

It is also possible, if not probable that the objective reality is actually a perception by a different group of people and may not be as real as implied. This needs to be addressed.

Line 84: The statement of objectives really doesn’t encapsulate the description of the survey questions described in line 178-192. Please rewrite.

This has been rewritten to encapsulate the purpose of the survey.

Line 105: “a population of nearly 2.5 million people in xxx km2, representing…”

This has been changed

Line 133: Were the hired Fulani interviewed? What proportion of the population are they?

Yes, the few (2%) were interviewed. Their number of Fulani in Ghana are not known, but they are estimated to be more than 14,000. They are in minority and do live in isolated areas round the outskirts of the local communities. so that it difficult to have their exact numbers. This is because they are normally nomadic and gradually becoming sedentary in some areas of the Northern region.

What proportion of the work do they do? They take care of cattle in the community thus communities members com together and hire a Fulani as herdsman. Do they perform the work of burning?

The study did not specifically consider that who performs burning.

Could it be that the landowners’ perceptions about fire are different from those of the people who actually do the burning if they are hired workers?

People who burn for cropping are the same people who prepare the land for planting which normally done by men in the house hold or farm family likewise the Pastoralist. But the fact is that there no specific designated areas for grazing and farming. During the dry season the farms are also used as grazing fields in addition to the areas within the savanna as the natural pastures.

We did not consider who does the burning, we were interested in whether fire used or not, If yes, what time of the year, how many times and reasons for the fire use.

Line 169: “People were asked questions in their respective first languages on…”

Corrected

Line 207: please replace “dialects” with “languages”. The word “dialects” has racist overtones as it is far more frequently used for people of colour than of white people for their native language. (to be clear in popular usage white people speak languages, black people speak dialects - this is a racist word usage even if people don't realise it).

Well, noted and replaced.

Line 213: Full stop required after statistics.

Done

Line 230: 21% is about one fifth. That is not a majority. “of six age groups, the largest was 26-32 years old”

This was deleted and corrected before the comments came in so may not appear in track changes.

Line 238: 70 persons per household to my understanding is a small village. Please explain your definition of a household. I doubt all 70 sleeps under the same roof.

In the north of Ghana, a traditional household refers to a big house with several rooms belonging to the sons of (with the wives and children) one father or grandfather who is normally the head of the household. The house is normally built in circular style with a big shared compound being the centre of the house. The size of the household normally depends of number of sons who live with the father. We did not ask of the number of roofs or “apartments” in the compound but the number of people in the large compound house. The members of the house normally eat from the same kitchen and cooking pots.

A cross sectional view of the inside of a circular traditional household

Back view of a traditional compound household com

Do you use this information on household size? If not maybe delete sentence as irrelevant.

We have not used but we though may be relevant, gives an idea on how many people live a household may have a direct relationship since the number and size of households are dependent determine the size and number of crops farms the family controls. Regression analysis actually did not predict for the household size.

Line 403: Although you are citing a paper with these temperatures, they are really meaningless in terms of describing fire behaviour. There spatial and temporal variations in fire behaviour between early and late season are such that ascribing temperature values is invalid. May be just say that late fires are of higher intensity than early fires.

Yes, we deleted as suggested, but some studies in the Guinea savanna have shown that early burning have higher temperature than late burning. So, we thought it might be fine to be specific here.

Line 414: be good to point out that Striga is a parasitic plant that reduces crop yields.
Line 442 “respectively, that firebreaks were burnt to reduce fire risks…”

This has been rephrased

Line 490: “occur”

Line 540: the statement “could have enormous impacts on the environment” is rather meaningless. The intention of controlled burning is to impact the environment through such processes as removing stubble, weeds, fire hazard around dwellings, and causing game to move. What are these “enormous impacts” and do they arise from a single event (e.g., death of rainforest trees, or decline in air quality) or as a result of the fire regime (e.g., soil degradation, shift in tree/grass balance). If they are the result of a regime, then maybe they are not enormous. Are there positive environmental impacts of fires? This has been rephrased.

THANKS FOR THE COMMENTS
